# Supplementary material for: Long-term risk of death after tuberculosis diagnosis and treatment
Source: Nat Med. 2026 Mar 19;32(5):1927–34. doi: 10.1038/s41591-026-04294-w (PMC13190314; doi:10.1038/s41591-026-04294-w)
Supplement: Supplementary file 1 — Supplementary Tables 1-16, Supplementary figs. 1-5 [file 41591_2026_4294_MOESM1_ESM.pdf]

nature medicine

Supplementary information https://doi.org/10.1038/s41591-026-04294-w

Long-term risk of death after tuberculosis diagnosis and treatment

In the format provided by the authors and unedited

Table of Contents

Supplementary Table 1. Missing data characteristics. 3

Supplementary Table 2: Baseline characteristics of matched participants in the diagnosed and treated cohorts. 3

Supplementary Table 3: Baseline characteristics of matched and unmatched persons in the diagnosed and treated cohorts. 6

Supplementary Table 4. Estimated risk of death by cause-specific comparing diagnosed Tuberculosis cases to non-exposed controls. 7

Supplementary Table 5. Estimated risk of death by cause-specific (defined by ICD-10 chapters) comparing treated Tuberculosis cases to non-exposed controls. 11

Supplementary Table 6. Estimated risk of death by cause-specific (defined by ICD-10 chapters) comparing household Tuberculosis contacts to non-exposed controls. 15

Supplementary Table 7. Estimated risk of death by cause-specific (defined by ICD-10 blocks) comparing diagnosed Tuberculosis cases to non-exposed controls. 19

Supplementary Table 8. Estimated risk of death by cause-specific (defined by ICD-10 blocks) comparing treated Tuberculosis cases to non-exposed controls. 22

Supplementary Table 9. Estimated risk of death by cause-specific (defined by ICD-10 blocks) comparing household Tuberculosis contacts to non-exposed controls. 25

Supplementary Table 10. Estimated risk of death of natural causes (excluding external causes, TB and HIV deaths) comparing diagnosed Tuberculosis cases to non-exposed controls by subgroup. 29

Supplementary Table 11. Estimated risk of death of natural causes (excluding external causes, TB and HIV deaths) comparing treated Tuberculosis cases to non-exposed controls by subgroup. 36

Supplementary Table 12. Estimated risk of death by cause-specific (defined by ICD-10 chapters) comparing diagnosed Tuberculosis cases to non-exposed controls in Females. 43

Supplementary Table 13. Estimated risk of death by cause-specific (defined by ICD-10 chapters) comparing diagnosed Tuberculosis cases to non-exposed controls in Males. 47

Supplementary Table 14. Estimated risk of death by cause-specific (defined by ICD-10 chapters) comparing treated Tuberculosis cases to non-exposed controls in Females. 51

Supplementary Table 15. Estimated risk of death by cause-specific (defined by ICD-10 chapters) comparing treated Tuberculosis cases to non-exposed controls in Males. 55

Supplementary Table 16. Estimated risk of death by cause-specific comparing diagnosed Tuberculosis cases to household contacts. 59

Supplementary Figure 1. Cumulative incidence curve of all-cause mortality in the diagnosed tuberculosis cases, and the unexposed control group. Shaded areas indicate the 95% confidence intervals. 62

Supplementary Figure 2. Cumulative incidence curve of cause-specific mortality in the diagnosed tuberculosis cases and the unexposed control group. a) HIV and TB deaths, b) Cancer, endocrine, respiratory and cardiovascular deaths 63

Supplementary Figure 3. Cumulative incidence curve of all-cause mortality in the treated tuberculosis cases and the unexposed control group. Shaded areas indicate the 95% confidence intervals. 64

Supplementary Figure 4. Cumulative incidence curve of cause-specific mortality in the treated tuberculosis cases and the unexposed control group. a) HIV and TB deaths, b) Cancer, endocrine, respiratory and cardiovascular deaths 65

Supplementary Figure 5. Cumulative incidence curve of all-cause mortality in the household tuberculosis case contacts and the unexposed control group. Shaded areas indicate the 95% confidence intervals. 66

Supplementary Table 1. Missing data characteristics.

Characteristic	Cases with missing N = 41,210	Cases without missing N = 209,598
**Age at baseline**	25 (17 - 38)	26 (16 - 40)
**Sex**		
Female	14474 (35.1)	76252 (36.4)
**Race/Ethnicity**		
White	7512 (27.6)	55591 (26.5)
Black	3299 (12.1)	26257 (12.5)
Mixed	16111 (59.1)	124472 (59.4)
Asian	86 (0.3)	616 (0.3)
Indigenous	251 (0.9)	2662 (1.3)
Missing	13951	
**Geographic region**		
North	4313 (10.5)	24596 (11.7)
Northeast	14281 (34.7)	71265 (34.0)
Southeast	15943 (38.8)	81060 (38.7)
South	4908 (11.9)	23373 (11.2)
Central-west	1663 (4.0)	9304 (4.4)
Missing	102	
**Water system**		
Public system	25129 (79.3)	159284 (76)
Water well	4465 (14.1)	33969 (16.2)
Other	2109 (6.7)	16345 (7.8)
Missing	9507	
**Location of household**		
Urban	30310 (87.2)	179398 (85.6)
Rural	4440 (12.8)	30200 (14.4)
Missing	6460	
**Overcrowd (>2)**	4893 (15.4)	34468 (16.4)
**Diabetes**	2004 (4.9)	11389 (5.4)
**HIV**	3825 (9.3)	17055 (8.1)
**Material of household**		
Masonry/brick	24845 (78.4)	158766 (75.7)
Coated Taipa	614 (1.9)	4889 (2.3)
Uncoated Taipa	519 (1.6)	4731 (2.3)
Wood	4022(12.7)	29384 (14.0)
Other	1705 (5.4)	11828 (5.6)
Missing	9505	
**Tuberculosis classification**		
Pulmonary	34,608 (84%)	178,217 (85%)
Extrapulmonary	5373 (13.0)	25587 (12.2)
Pulmonary+Extrapulmonary	1206 (2.9)	5720 (2.7)
Missing	23 (0.1)	74 (0.1)

Supplementary Table 2: Baseline characteristics of matched participants in the diagnosed and treated cohorts.

	Diagnosed Tuberculosis		Treated Tuberculosis	
**Characteristic**	**Unexposed N = 185,921**	**Exposed N = 185,921**	**Unexposed N = 111,871**	**Exposed N = 111,871**
**Age (years), at CadUnico registration – median (IQR)**	25 (15 - 39)	25 (15 - 39)	25 (15 - 39)	25 (15 - 39)
**Age (years), at index date – median (IQR)**	33 (24 – 47)	33 (24 – 47)	33 (25 – 47)	33 (25 – 47)
**Age group, at index date**				

<18	12,520 (6.7%)	12,520 (6.7%)	6,963 (6.2%)	6,963 (6.2%)
18-59	154,081 (82.9%)	154,081 (82.9%)	92,998 (83.1%)	92,998 (83.1%)
≥60	19,320 (10.4%)	19,320 (10.4%)	11,910 (10.6%)	11,910 (10.6%)
**Sex**				
Female	68,470 (36.8%)	68,470 (36.8%)	42,829 (38.3%)	42,829 (38.3%)
Male	117,451 (63.2%)	117,451 (63.2%)	69,042 (61.7%)	69,042 (61.7%)
**Race/Ethnicity**				
White	49,599 (26.7%)	49,599 (26.7%)	28,695 (25.7%)	28,695 (25.7%)
Black	21,183 (11.4%)	21,183 (11.4%)	12,116 (10.8%)	12,116 (10.8%)
Mixed	113,311 (60.9%)	113,311 (60.9%)	69,769 (62.4%)	69,769 (62.4%)
Asian	264 (0.1%)	264 (0.1%)	156 (0.1%)	156 (0.1%)
Indigenous	1,564 (0.8%)	1,564 (0.8%)	1,135 (1.0%)	1,135 (1.0%)
**Education Level**				
No school	24,436 (13.1%)	24,436 (13.1%)	14,806 (13.2%)	14,806 (13.2%)
Nursery	1,503 (0.8%)	1,503 (0.8%)	951 (0.9%)	951 (0.9%)
Infant School	1,720 (0.9%)	1,720 (0.9%)	1,033 (0.9%)	1,033 (0.9%)
Elementary School	70,454 (37.9%)	70,454 (37.9%)	41,865 (37.4%)	41,865 (37.4%)
Middle school	65,170 (35.1%)	65,170 (35.1%)	38,951 (34.8%)	38,951 (34.8%)
High School	21,962 (11.8%)	21,962 (11.8%)	13,833 (12.4%)	13,833 (12.4%)
Higher education	676 (0.4%)	676 (0.4%)	432 (0.4%)	432 (0.4%)
**Overcrowded***	27,968 (15.0%)	27,968 (15.0%)	16,240 (14.5%)	16,240 (14.5%)
**Water system**				
Public system	147,196 (79.2%)	147,196 (79.2%)	87,296 (78.0%)	87,296 (78.0%)
Water well	26,747 (14.4%)	26,747 (14.4%)	17,381 (15.5%)	17,381 (15.5%)
Other	11,978 (6.4%)	11,978 (6.4%)	7,194 (6.4%)	7,194 (6.4%)
**Location of household**				
Urban	164,096 (88.3%)	164,096 (88.3%)	97,496 (87.2%)	97,496 (87.2%)
Rural	21,825 (11.7%)	21,825 (11.7%)	14,375 (12.8%)	14,375 (12.8%)
**Material of household**				
Masonry/brick	147,173 (79.2%)	147,173 (79.2%)	87,086 (77.8%)	87,086 (77.8%)
Coated Taipa	2,929 (1.6%)	2,929 (1.6%)	1,926 (1.7%)	1,926 (1.7%)
Uncoated Taipa	2,930 (1.6%)	2,930 (1.6%)	2,003 (1.8%)	2,003 (1.8%)
Wood	24,712 (13.3%)	24,712 (13.3%)	15,674 (14.0%)	15,674 (14.0%)
Other	8,177 (4.4%)	8,177 (4.4%)	5,182 (4.6%)	5,182 (4.6%)
**Year of CadUnico registration**				
2004-2006	105,041 (56.5%)	105,041 (56.5%)	63,757 (57.0%)	63,757 (57.0%)
2007-2009	41,269 (22.2%)	41,269 (22.2%)	25,078 (22.4%)	25,078 (22.4%)
2010-2012	23,137 (12.4%)	23,137 (12.4%)	13,764 (12.3%)	13,764 (12.3%)
2013-2015	11,417 (6.1%)	11,417 (6.1%)	7,138 (6.4%)	7,138 (6.4%)
2016-2018	5,057 (2.7%)	5,057 (2.7%)	2,134 (1.9%)	2,134 (1.9%)
**Diabetes**	-	9,768 (5.3%)	-	5,908 (5.3%)

**HIV**	-	15,286 (8.2%)	-	6,031 (5.4%)
**Geographic region**				
North	21,560 (11.6%)	21,560 (11.6%)	14,525 (13.0%)	14,525 (13.0%)
Northeast	61,861 (33.3%)	61,861 (33.3%)	39,603 (35.4%)	39,603 (35.4%)
Southeast	75,008 (40.3%)	75,008 (40.3%)	40,272 (36.0%)	40,272 (36.0%)
South	19,828 (10.7%)	19,828 (10.7%)	12,619 (11.3%)	12,619 (11.3%)
Central west	7,664 (4.1%)	7,664 (4.1%)	4,852 (4.3%)	4,852 (4.3%)
**Tuberculosis classification**				
Pulmonary		157,540 (84.7%)		96,328 (86.1%)
Extrapulmonary		23,134 (12.4%)		13,110 (11.7%)
Both		5,178 (2.8%)		2,433 (2.2%)
Missing		69 (<0.1%)		0 (0%)
**Thorax Radiographic – positive**		140,122 (75.4%)		84,728 (75.7%)
Not performed		34,914 (18.8%)		21,049 (18.8%)
**Sputum culture– positive**		29,000 (15.6%)		16,736 (15.0%)
Not performed		155,321 (83.5%)		94,299 (84.3%)
**Molecular test– positive**		19,129 (10.3%)		10,660 (9.5%)
Not performed		162,252 (87.3%)		98,822 (88.3%)
**Bacilloscopic – positive**		103,898 (55.9%)		64,393 (57.6%)
Not performed		42,366 (22.8%)		23,906 (21.4%)
**Histopathology – positive**		18,479 (9.9%)		11,332 (10.1%)
Not performed		166,113 (89.3%)		99,752 (89.2%)
**Laboratory diagnosis**		127,838 (68.8%)		78,239 (69.9%)
**Radiographic or laboratory diagnosis**		174,484 (93.8%)		105,915 (94.7%)
**Any laboratory diagnosis**		127,838 (69%)		78,239 (70%)
**Radiographic or any laboratory diagnosis**		174,484 (94%)		105,915 (95%)
**Location extrapulmonary TB:**				
Pleura		11,873 (41.9%)		7,136 (45.9%)
Lymph nodes		6,072 (21.4%)		3,540 (22.8%)
Bone and joints		1,060 (3.7%)		557 (3.6%)
Miliary		2,156 (7.6%)		830 (5.3%)
Central nervous system		1,527 (5.4%)		442 (2.8%)
Others		5,624 (19.9%)		3,038 (19.6%)

Supplementary Table 3: Baseline characteristics of matched and unmatched persons in the diagnosed and treated cohorts.

Characteristic	Diagnosed		Treated	
	Matched N = 185,921	Unmatched N = 23,677	Matched N = 111,690	Unmatched N = 14,118
**Age at baseline**	25 (15, 39)	36 (24, 51)	25 (15, 39)	36 (23, 50)
**Sex**				
Female	68,470 (36.8%)	7,781 (32.9%)	42,829 (38.3%)	4,853 (34.4%)
Male	117,451 (63.2%)	15,896 (67.1%)	69,042 (61.7%)	9,265 (65.6%)
**Race/Ethnicity**				
White	49,599 (26.7%)	5,992 (25.3%)	28,695 (25.7%)	3,499 (24.8%)
Black	21,183 (11.4%)	5,075 (21.4%)	12,116 (10.8%)	2,975 (21.1%)
Mixed	113,311 (60.9%)	11,160 (47.1%)	69,769 (62.4%)	6,652 (47.1%)
Asian	264 (0.1%)	352 (1.5%)	156 (0.1%)	212 (1.5%)
Indigenous	1,564 (0.8%)	1,098 (4.6%)	1,135 (1.0%)	780 (5.5%)
**Geographic region**				
North	21,560 (11.6%)	3,036 (12.8%)	14,525 (13.0%)	1,943 (13.8%)
Northeast	61,861 (33.3%)	9,404 (39.7%)	39,603 (35.4%)	5,797 (41.1%)
Southeast	75,008 (40.3%)	6,051 (25.6%)	40,272 (36.0%)	3,136 (22.2%)
South	19,828 (10.7%)	3,546 (15.0%)	12,619 (11.3%)	2,178 (15.4%)
Central west	7,664 (4.1%)	1,640 (6.9%)	4,852 (4.3%)	1,064 (7.5%)
**Education Level**				
No school	24,436 (13.1%)	6,416 (27.1%)	14,806 (13.2%)	3,726 (26.4%)
Nursery	1,503 (0.8%)	879 (3.7%)	951 (0.9%)	519 (3.7%)
Infant School	1,720 (0.9%)	401 (1.7%)	1,033 (0.9%)	257 (1.8%)
Elementary school	70,454 (37.9%)	8,271 (34.9%)	41,865 (37.4%)	4,872 (34.5%)
Middle school	65,170 (35.1%)	5,201 (22.0%)	38,951 (34.8%)	3,201 (22.7%)
High School	21,962 (11.8%)	2,220 (9.4%)	13,833 (12.4%)	1,368 (9.7%)
Higher education	676 (0.4%)	289 (1.2%)	432 (0.4%)	175 (1.2%)
**Water system**				
Public system	147,196 (79.2%)	12,088 (51.1%)	87,296 (78.0%)	7,079 (50.1%)
Water well	26,747 (14.4%)	7,222 (30.5%)	17,381 (15.5%)	4,474 (31.7%)
Other	11,978 (6.4%)	4,367 (18.4%)	7,194 (6.4%)	2,565 (18.2%)
**Location of household**				
Urban	164,096 (88.3%)	15,302 (64.6%)	97,496 (87.2%)	8,856 (62.7%)
Rural	21,825 (11.7%)	8,375 (35.4%)	14,375 (12.8%)	5,262 (37.3%)
**Overcrowd (>2)**	27,968 (15.0%)	6,500 (27.5%)	16,240 (14.5%)	3,841 (27.1%)
**Diabetes**	9,768 (5.3%)	1,621 (6.8%)	5,908 (5.3%)	942 (6.7%)
**HIV**	15,286 (8.2%)	1,770 (7.5%)	6,031 (5.4%)	693 (4.9%)
**Material of household**				
Masonry/brick	147,173 (79.2%)	11,593 (49.0%)	87,086 (77.8%)	6,819 (48.3%)
Coated Taipa	2,929 (1.6%)	1,960 (8.3%)	1,926 (1.7%)	1,248 (8.8%)
Uncoated Taipa	2,930 (1.6%)	1,801 (7.6%)	2,003 (1.8%)	1,091 (7.7%)
Wood	24,712 (13.3%)	4,672 (19.7%)	15,674 (14.0%)	2,721 (19.3%)
Other	8,177 (4.4%)	3,651 (15.4%)	5,182 (4.6%)	2,239 (15.9%)
**Year of CadUnico registration**				
2004-2006	105,041 (56.5%)	11,165 (47.2%)	63,757 (57.0%)	6,880 (48.7%)
2007-2009	41,269 (22.2%)	6,568 (27.7%)	25,078 (22.4%)	4,011 (28.4%)
2010-2012	23,137 (12.4%)	3,425 (14.5%)	13,764 (12.3%)	1,959 (13.9%)
2013-2015	11,417 (6.1%)	1,827 (7.7%)	7,138 (6.4%)	1,016 (7.2%)
2016-2018	5,057 (2.7%)	692 (2.9%)	2,134 (1.9%)	252 (1.8%)
**Tuberculosis classification**				
Pulmonary	157,540 (84.7%)	20,677 (87.3%)	96,328 (86.1%)	12,575 (89.1%)
Extrapulmonary	23,134 (12.4%)	2,453 (10.4%)	13,110 (11.7%)	1,320 (9.3%)
Pulmonary + Extrapulmonary	5,178 (2.8%)	542 (2.3%)	2,433 (2.2%)	223 (1.6%)
Missing	69 (0.0%)	5 (0.0%)		

Supplementary Table 4. Estimated risk of death by cause-specific comparing diagnosed Tuberculosis cases to non-exposed controls.

	Number of events		Risk per 100,000				
Time - Days	Unexposed	Exposed	Unexposed	Exposed	Risk difference per 100,000 - 95% CI	Risk ratio - 95% CI	Incidence rate ratio - 95% CI
**Cause: All-cause mortality**							
30	87	5066	47.0 (37.3 to 57.3)	2732.7 (2661.3 to 2808.5)	2685.7 (2612.4 to 2762.0)	58.10 (47.05 to 73.04)	59.23 (47.98 to 74.43)
90	165	2869	137.8 (121.6 to 153.6)	4306.5 (4213.2 to 4397.9)	4168.7 (4077.9 to 4264.0)	31.25 (27.99 to 35.57)	18.03 (15.59 to 21.18)
180	210	1939	256.9 (235.7 to 278.7)	5403.9 (5296.5 to 5505.8)	5146.9 (5036.2 to 5252.4)	21.03 (19.34 to 22.91)	9.69 (8.51 to 11.35)
365	464	2293	534.9 (503.3 to 568.1)	6773.4 (6645.3 to 6888.8)	6238.5 (6114.1 to 6356.2)	12.66 (11.87 to 13.45)	5.24 (4.77 to 5.79)
730	768	2988	1045.9 (997.1 to 1091.3)	8754.2 (8611.8 to 8892.5)	7708.3 (7563.5 to 7858.3)	8.37 (8.01 to 8.80)	4.19 (3.89 to 4.53)
1095	737	2015	1612.4 (1551.3 to 1673.8)	10303.4 (10149.7 to 10452.0)	8691.0 (8530.8 to 8853.8)	6.39 (6.15 to 6.66)	2.98 (2.75 to 3.24)
1460	672	1652	2216.2 (2135.2 to 2295.2)	11781.0 (11628.9 to 11953.1)	9564.8 (9388.0 to 9733.6)	5.32 (5.10 to 5.52)	2.70 (2.49 to 2.96)
1825	564	1369	2809.3 (2715.1 to 2902.0)	13214.7 (13053.9 to 13399.4)	10405.4 (10218.2 to 10618.0)	4.70 (4.55 to 4.88)	2.69 (2.45 to 3.00)
2190	425	1076	3346.8 (3235.1 to 3455.2)	14566.4 (14374.9 to 14764.9)	11219.5 (11014.3 to 11438.2)	4.35 (4.22 to 4.51)	2.84 (2.55 to 3.17)
2555	357	851	3901.2 (3781.6 to 4034.6)	15876.5 (15667.3 to 16107.0)	11975.3 (11750.5 to 12214.9)	4.07 (3.94 to 4.21)	2.70 (2.36 to 3.03)
2920	281	590	4452.2 (4310.4 to 4598.7)	17021.8 (16795.9 to 17252.2)	12569.6 (12305.6 to 12820.8)	3.82 (3.70 to 3.95)	2.39 (2.10 to 2.77)
3285	221	512	5022.9 (4871.1 to 5200.8)	18325.8 (18076.2 to 18568.1)	13302.9 (13000.8 to 13589.4)	3.65 (3.53 to 3.76)	2.66 (2.28 to 3.07)
3650	173	305	5628.5 (5448.7 to 5837.3)	19393.4 (19103.1 to 19668.8)	13764.8 (13432.6 to 14065.7)	3.45 (3.32 to 3.57)	2.04 (1.70 to 2.47)
4015	107	212	6200.7 (5991.0 to 6407.9)	20524.7 (20194.4 to 20838.3)	14324.0 (13961.1 to 14715.0)	3.31 (3.19 to 3.44)	2.31 (1.84 to 2.98)
4380	61	118	6814.6 (6566.6 to 7098.8)	21693.1 (21322.8 to 22056.1)	14878.5 (14443.6 to 15344.1)	3.18 (3.06 to 3.32)	2.28 (1.70 to 3.01)
4745	26	40	7491.7 (7102.3 to 7895.7)	22727.4 (22209.8 to 23247.2)	15235.7 (14561.0 to 15889.9)	3.03 (2.86 to 3.21)	1.83 (1.16 to 3.15)
5110	7	5	8041.5 (7463.7 to 8583.2)	23209.3 (22543.3 to 23976.3)	15167.8 (14343.2 to 16015.5)	2.89 (2.69 to 3.11)	***
**Cause: Natural - excluding (HIV/TB and external causes)**							
30	54	1524	29.2 (22.2 to 37.3)	822.4 (782.8 to 860.9)	793.2 (753.7 to 832.9)	28.18 (21.68 to 37.04)	28.70 (22.06 to 37.73)
90	116	1119	93.0 (80.4 to 106.1)	1436.3 (1386.8 to 1488.1)	1343.3 (1291.5 to 1394.4)	15.45 (13.35 to 17.86)	10.00 (8.27 to 12.24)
180	166	842	187.2 (168.8 to 205.4)	1912.9 (1847.8 to 1974.1)	1725.7 (1660.8 to 1789.2)	10.22 (9.24 to 11.33)	5.32 (4.61 to 6.45)
365	337	1151	389.2 (360.5 to 418.5)	2600.9 (2526.9 to 2667.0)	2211.7 (2139.2 to 2286.0)	6.68 (6.20 to 7.26)	3.62 (3.25 to 4.06)
730	565	1692	765.6 (721.7 to 803.1)	3724.7 (3637.7 to 3814.5)	2959.2 (2861.1 to 3056.1)	4.87 (4.61 to 5.16)	3.22 (2.94 to 3.54)
1095	558	1237	1194.6 (1142.0 to 1249.7)	4676.7 (4571.1 to 4777.7)	3482.1 (3369.3 to 3597.3)	3.91 (3.73 to 4.12)	2.42 (2.19 to 2.68)
1460	515	1050	1658.2 (1586.3 to 1726.6)	5615.9 (5502.2 to 5733.7)	3957.7 (3816.7 to 4089.4)	3.39 (3.24 to 3.54)	2.24 (2.02 to 2.52)
1825	435	903	2116.3 (2033.0 to 2197.4)	6562.7 (6433.3 to 6699.4)	4446.4 (4277.5 to 4602.6)	3.10 (2.97 to 3.24)	2.30 (2.06 to 2.60)
2190	348	731	2556.2 (2456.2 to 2652.8)	7479.2 (7332.0 to 7626.3)	4923.0 (4740.6 to 5082.9)	2.93 (2.81 to 3.05)	2.35 (2.08 to 2.68)

2555	270	587	2975.2 (2871.2 to 3085.9)	8383.6 (8226.5 to 8538.4)	5408.4 (5221.7 to 5585.2)	2.82 (2.71 to 2.93)	2.46 (2.12 to 2.84)
2920	213	405	3392.7 (3271.1 to 3514.8)	9170.9 (8995.0 to 9338.6)	5778.2 (5572.7 to 5977.1)	2.70 (2.60 to 2.82)	2.17 (1.87 to 2.55)
3285	179	372	3853.5 (3717.2 to 3998.4)	10121.6 (9919.4 to 10339.9)	6268.1 (6019.2 to 6517.4)	2.63 (2.52 to 2.74)	2.39 (2.02 to 2.79)
3650	136	234	4327.3 (4166.6 to 4484.2)	10945.4 (10719.2 to 11176.8)	6618.1 (6344.0 to 6888.7)	2.53 (2.43 to 2.64)	1.99 (1.64 to 2.45)
4015	87	172	4788.7 (4601.5 to 4967.6)	11863.0 (11615.3 to 12125.6)	7074.3 (6776.7 to 7386.9)	2.48 (2.37 to 2.59)	2.30 (1.82 to 3.03)
4380	53	98	5331.4 (5094.2 to 5576.5)	12835.1 (12513.0 to 13150.4)	7503.7 (7139.6 to 7885.1)	2.41 (2.30 to 2.53)	2.18 (1.56 to 2.97)
4745	21	30	5901.9 (5553.1 to 6267.3)	13599.3 (13177.5 to 14053.9)	7697.4 (7101.4 to 8239.1)	2.30 (2.15 to 2.45)	1.70 (0.96 to 3.06)
5110	7	4	6451.7 (5886.5 to 6975.2)	13947.2 (13413.3 to 14553.7)	7495.5 (6652.7 to 8257.8)	2.16 (1.96 to 2.37)	***
**Cause: Cancer**							
30	9	245	4.9 (2.2 to 8.1)	132.3 (117.2 to 149.3)	127.4 (112.3 to 145.5)	27.19 (15.65 to 60.28)	27.69 (15.94 to 61.40)
90	19	298	15.3 (9.8 to 20.2)	295.9 (272.8 to 319.7)	280.6 (257.1 to 306.3)	19.30 (14.39 to 31.09)	16.26 (11.08 to 29.63)
180	24	250	29.0 (21.2 to 36.3)	437.3 (409.0 to 467.2)	408.4 (378.2 to 438.2)	15.10 (12.04 to 20.75)	10.93 (7.80 to 17.16)
365	59	338	64.5 (53.4 to 76.1)	639.2 (602.0 to 678.9)	574.7 (536.8 to 613.9)	9.91 (8.37 to 12.08)	6.08 (4.70 to 8.16)
730	112	378	138.8 (119.8 to 157.1)	889.7 (849.7 to 940.1)	751.0 (706.3 to 805.7)	6.41 (5.62 to 7.56)	3.63 (3.00 to 4.71)
1095	95	233	212.1 (186.5 to 239.6)	1069.1 (1021.2 to 1126.7)	856.9 (798.0 to 921.5)	5.04 (4.46 to 5.80)	2.67 (2.10 to 3.42)
1460	104	164	305.8 (279.8 to 336.2)	1215.7 (1161.7 to 1274.3)	909.9 (846.8 to 976.1)	3.98 (3.55 to 4.43)	1.73 (1.35 to 2.27)
1825	83	169	393.5 (360.1 to 428.3)	1392.8 (1328.0 to 1458.1)	999.3 (927.9 to 1071.3)	3.54 (3.19 to 3.93)	2.26 (1.78 to 2.88)
2190	62	120	472.6 (429.5 to 513.5)	1542.6 (1474.1 to 1617.8)	1070.0 (989.1 to 1149.9)	3.26 (2.96 to 3.60)	2.17 (1.62 to 3.03)
2555	45	97	541.7 (492.2 to 587.5)	1690.6 (1614.3 to 1772.5)	1148.9 (1059.8 to 1237.6)	3.12 (2.86 to 3.47)	2.44 (1.73 to 3.43)
2920	43	73	627.5 (575.2 to 677.1)	1831.2 (1746.5 to 1917.7)	1203.6 (1107.6 to 1299.7)	2.92 (2.66 to 3.19)	1.94 (1.38 to 2.92)
3285	36	63	719.4 (651.7 to 780.2)	1992.3 (1898.2 to 2080.9)	1273.0 (1163.9 to 1377.3)	2.77 (2.53 to 3.04)	2.01 (1.36 to 3.02)
3650	31	46	827.5 (756.0 to 895.6)	2155.8 (2051.2 to 2259.9)	1328.3 (1206.4 to 1446.6)	2.61 (2.37 to 2.84)	1.72 (1.07 to 2.81)
4015	24	24	956.7 (874.0 to 1041.2)	2285.0 (2176.2 to 2404.8)	1328.3 (1197.5 to 1463.1)	2.39 (2.17 to 2.64)	1.17 (0.65 to 1.99)
4380	13	22	1085.5 (976.0 to 1195.4)	2496.7 (2348.2 to 2641.9)	1411.2 (1227.7 to 1576.9)	2.30 (2.05 to 2.58)	1.99 (0.99 to 4.11)
4745	6	5	1243.5 (1086.2 to 1424.0)	2617.6 (2433.4 to 2792.6)	1374.1 (1115.5 to 1607.8)	2.11 (1.81 to 2.45)	***
5110	1	1	1292.2 (1116.2 to 1504.8)	2694.4 (2452.9 to 2927.8)	1402.2 (1088.6 to 1664.4)	2.09 (1.75 to 2.44)	***
**Cause: Cardiovascular**							
30	13	189	7.0 (3.2 to 10.8)	102.0 (87.7 to 117.1)	95.0 (79.9 to 110.1)	14.54 (8.93 to 28.66)	14.79 (9.08 to 29.13)
90	38	158	27.9 (20.2 to 35.3)	188.7 (167.8 to 209.7)	160.8 (137.9 to 181.6)	6.76 (5.11 to 9.38)	4.31 (3.12 to 6.25)
180	53	146	58.0 (47.2 to 68.0)	271.4 (246.1 to 296.4)	213.4 (183.9 to 239.7)	4.68 (3.84 to 5.84)	2.89 (2.16 to 4.02)
365	121	202	130.4 (113.1 to 148.1)	391.9 (360.7 to 421.8)	261.5 (225.2 to 295.9)	3.00 (2.58 to 3.49)	1.77 (1.41 to 2.27)
730	175	315	247.1 (223.1 to 270.9)	600.8 (560.3 to 639.1)	353.8 (306.2 to 397.2)	2.43 (2.16 to 2.76)	1.94 (1.61 to 2.35)
1095	205	263	404.7 (372.8 to 438.5)	803.7 (755.1 to 845.6)	399.0 (338.9 to 453.7)	1.99 (1.79 to 2.20)	1.40 (1.17 to 1.69)
1460	166	259	553.9 (513.2 to 594.9)	1036.0 (981.8 to 1084.1)	482.2 (414.6 to 546.5)	1.87 (1.71 to 2.05)	1.72 (1.40 to 2.06)
1825	148	194	709.2 (658.0 to 759.4)	1240.6 (1177.8 to 1297.5)	531.4 (455.0 to 605.7)	1.75 (1.61 to 1.91)	1.46 (1.20 to 1.79)

2190	122	160	863.2 (806.8 to 920.0)	1441.6 (1374.2 to 1503.0)	578.4 (493.8 to 654.9)	1.67 (1.54 to 1.80)	1.47 (1.15 to 1.82)
2555	100	122	1019.7 (960.8 to 1084.4)	1631.1 (1554.8 to 1701.1)	611.4 (525.5 to 691.6)	1.60 (1.49 to 1.71)	1.38 (1.07 to 1.78)
2920	72	98	1159.9 (1097.7 to 1232.3)	1821.9 (1739.1 to 1900.9)	662.0 (555.8 to 757.0)	1.57 (1.46 to 1.68)	1.55 (1.16 to 2.14)
3285	61	72	1319.2 (1243.8 to 1404.6)	2009.4 (1919.5 to 2100.8)	690.1 (569.0 to 810.2)	1.52 (1.41 to 1.64)	1.36 (0.96 to 1.92)
3650	57	57	1518.3 (1427.6 to 1612.1)	2210.4 (2103.5 to 2313.0)	692.2 (563.8 to 826.2)	1.46 (1.35 to 1.57)	1.16 (0.81 to 1.71)
4015	24	35	1638.2 (1536.3 to 1736.3)	2392.8 (2272.9 to 2502.5)	754.5 (607.3 to 895.3)	1.46 (1.35 to 1.57)	1.70 (1.03 to 2.95)
4380	20	28	1832.4 (1701.2 to 1961.4)	2676.5 (2527.8 to 2826.9)	844.1 (658.1 to 1037.4)	1.46 (1.34 to 1.60)	1.65 (1.02 to 3.01)
4745	7	5	2023.0 (1833.5 to 2234.9)	2791.8 (2610.5 to 2975.7)	768.8 (494.9 to 1038.0)	1.38 (1.22 to 1.56)	***
5110	1	1	2102.5 (1870.7 to 2412.8)	2902.2 (2673.3 to 3198.8)	799.6 (404.6 to 1205.2)	1.38 (1.17 to 1.62)	***
**Cause: Endocrine**							
30	3	85	1.6 (0.0 to 3.8)	45.9 (36.7 to 56.1)	44.3 (34.5 to 54.5)	***	***
90	11	64	7.7 (3.8 to 12.1)	81.0 (67.9 to 94.7)	73.3 (59.8 to 88.1)	10.56 (6.64 to 21.58)	6.03 (3.46 to 14.74)
180	12	46	14.5 (8.9 to 20.0)	107.0 (91.8 to 122.9)	92.6 (76.8 to 110.1)	7.40 (5.21 to 12.10)	4.02 (2.33 to 9.09)
365	27	68	30.7 (22.6 to 39.0)	147.8 (130.1 to 166.6)	117.1 (97.2 to 137.6)	4.82 (3.68 to 6.72)	2.67 (1.78 to 4.19)
730	56	103	68.0 (55.8 to 80.9)	216.5 (195.2 to 238.9)	148.5 (121.8 to 173.4)	3.19 (2.58 to 3.99)	1.98 (1.47 to 2.78)
1095	35	90	94.9 (79.9 to 111.5)	285.3 (259.9 to 311.7)	190.4 (160.6 to 222.6)	3.01 (2.46 to 3.65)	2.80 (1.90 to 4.20)
1460	41	80	132.1 (114.2 to 153.4)	356.9 (325.7 to 387.3)	224.7 (188.7 to 262.5)	2.70 (2.26 to 3.21)	2.15 (1.50 to 3.12)
1825	29	60	162.6 (140.9 to 186.1)	419.9 (385.3 to 457.2)	257.2 (216.6 to 300.5)	2.58 (2.22 to 3.03)	2.30 (1.47 to 3.74)
2190	26	66	195.3 (170.2 to 221.8)	502.5 (461.4 to 541.4)	307.2 (258.0 to 356.9)	2.57 (2.20 to 3.03)	2.84 (1.80 to 4.73)
2555	21	45	227.9 (199.9 to 257.9)	571.3 (525.7 to 615.3)	343.4 (289.1 to 398.1)	2.51 (2.15 to 2.95)	2.42 (1.48 to 4.17)
2920	9	30	245.2 (215.1 to 278.4)	629.2 (579.5 to 679.5)	384.0 (323.2 to 446.9)	2.57 (2.20 to 3.02)	3.80 (1.97 to 11.41)
3285	9	36	268.5 (234.5 to 305.4)	722.5 (666.8 to 781.8)	454.0 (387.6 to 525.4)	2.69 (2.32 to 3.18)	4.59 (2.30 to 11.80)
3650	8	21	296.3 (255.2 to 337.0)	797.7 (727.9 to 862.4)	501.3 (428.3 to 580.2)	2.69 (2.30 to 3.20)	3.04 (1.42 to 9.54)
4015	3	15	312.0 (267.2 to 357.2)	878.7 (802.4 to 956.5)	566.7 (480.8 to 659.7)	2.82 (2.41 to 3.39)	***
4380	4	5	357.6 (299.4 to 429.8)	929.8 (841.0 to 1022.9)	572.2 (463.2 to 679.9)	2.60 (2.13 to 3.21)	***
4745	1	4	375.2 (311.5 to 456.0)	1020.0 (900.7 to 1148.8)	644.8 (506.3 to 792.3)	2.72 (2.18 to 3.47)	***
5110	1	0	432.6 (323.8 to 588.2)	1020.0 (900.7 to 1148.8)	587.4 (396.5 to 760.4)	2.36 (1.68 to 3.21)	***
**Cause: Respiratory**							
30	10	444	5.4 (2.2 to 9.2)	239.5 (218.7 to 260.0)	234.1 (213.1 to 257.0)	44.30 (26.43 to 106.00)	45.16 (26.95 to 108.00)
90	9	187	10.3 (6.5 to 15.2)	342.0 (315.8 to 365.8)	331.7 (303.5 to 355.6)	33.11 (22.21 to 53.95)	21.54 (12.08 to 52.12)
180	23	149	23.4 (15.9 to 31.8)	426.4 (396.2 to 453.0)	403.0 (370.6 to 431.3)	18.19 (13.44 to 26.95)	6.80 (4.51 to 11.73)
365	34	219	43.8 (33.2 to 54.0)	557.5 (521.8 to 590.2)	513.6 (476.9 to 550.1)	12.72 (10.21 to 16.93)	6.83 (4.96 to 10.34)
730	59	353	83.3 (70.2 to 98.7)	792.0 (749.6 to 836.3)	708.7 (663.7 to 754.7)	9.51 (7.95 to 11.37)	6.44 (4.99 to 8.38)
1095	75	229	140.7 (121.3 to 159.0)	967.8 (921.6 to 1014.7)	827.1 (777.6 to 877.2)	6.88 (5.99 to 8.03)	3.33 (2.60 to 4.37)
1460	58	215	193.2 (170.0 to 217.5)	1159.3 (1110.4 to 1213.9)	966.1 (909.5 to 1026.9)	6.00 (5.29 to 6.83)	4.08 (3.13 to 5.53)

1825	52	194	248.0 (218.9 to 275.3)	1362.2 (1303.2 to 1424.9)	1114.2 (1050.7 to 1187.4)	5.49 (4.90 to 6.28)	4.14 (3.20 to 5.82)
2190	44	130	303.8 (271.4 to 335.1)	1524.6 (1459.5 to 1599.0)	1220.8 (1150.5 to 1298.0)	5.02 (4.52 to 5.64)	3.31 (2.48 to 4.87)
2555	30	112	350.1 (314.2 to 387.2)	1697.5 (1625.6 to 1778.5)	1347.4 (1267.0 to 1433.9)	4.85 (4.33 to 5.42)	4.22 (2.92 to 6.89)
2920	25	79	399.2 (355.5 to 439.4)	1852.1 (1771.1 to 1938.9)	1452.8 (1367.6 to 1548.9)	4.64 (4.13 to 5.23)	3.60 (2.26 to 5.83)
3285	19	74	448.8 (400.0 to 495.3)	2037.8 (1948.8 to 2136.8)	1589.0 (1487.7 to 1688.8)	4.54 (4.06 to 5.13)	4.47 (2.87 to 7.97)
3650	12	43	490.3 (438.2 to 538.7)	2184.1 (2086.0 to 2295.8)	1693.8 (1577.8 to 1820.0)	4.45 (3.99 to 5.04)	4.15 (2.37 to 8.65)
4015	8	43	534.6 (471.4 to 592.9)	2413.2 (2300.6 to 2539.1)	1878.5 (1741.2 to 2023.7)	4.51 (4.03 to 5.15)	6.26 (3.25 to 18.25)
4380	6	16	597.6 (513.2 to 678.3)	2569.1 (2430.0 to 2731.3)	1971.6 (1810.1 to 2140.8)	4.30 (3.77 to 4.98)	3.14 (1.31 to 12.32)
4745	4	6	743.0 (586.2 to 936.2)	2755.0 (2572.7 to 2985.7)	2011.9 (1742.7 to 2268.3)	3.71 (2.93 to 4.68)	***
5110	1	1	806.4 (619.9 to 1031.9)	2830.0 (2599.3 to 3096.0)	2023.6 (1723.9 to 2367.3)	3.51 (2.71 to 4.65)	***
**Cause: External**							
30	31	59	16.8 (11.4 to 22.2)	31.9 (24.3 to 40.5)	15.1 (5.9 to 24.6)	1.90 (1.29 to 3.04)	1.94 (1.31 to 3.10)
90	45	72	41.5 (33.4 to 50.8)	71.4 (58.3 to 83.3)	29.9 (14.2 to 44.5)	1.72 (1.29 to 2.27)	1.66 (1.17 to 2.47)
180	43	151	65.8 (55.4 to 77.7)	157.0 (137.5 to 174.9)	91.1 (69.7 to 112.0)	2.38 (1.95 to 2.94)	3.69 (2.67 to 5.23)
365	117	286	135.9 (119.2 to 152.6)	328.3 (299.8 to 355.7)	192.4 (159.4 to 221.6)	2.42 (2.10 to 2.80)	2.59 (2.08 to 3.16)
730	191	481	262.6 (239.6 to 285.7)	648.2 (608.3 to 683.2)	385.6 (339.2 to 426.2)	2.47 (2.22 to 2.74)	2.71 (2.31 to 3.21)
1095	169	349	392.5 (363.9 to 425.2)	915.8 (866.5 to 964.5)	523.3 (463.4 to 578.2)	2.33 (2.13 to 2.55)	2.25 (1.85 to 2.72)
1460	142	266	519.3 (487.3 to 559.2)	1154.5 (1104.0 to 1213.8)	635.2 (571.8 to 697.9)	2.22 (2.04 to 2.39)	2.06 (1.69 to 2.52)
1825	115	226	639.7 (599.2 to 685.4)	1391.4 (1332.9 to 1452.5)	751.8 (681.7 to 829.4)	2.18 (2.02 to 2.35)	2.18 (1.75 to 2.77)
2190	72	177	730.7 (687.0 to 780.9)	1614.2 (1543.4 to 1683.5)	883.4 (805.4 to 966.3)	2.21 (2.05 to 2.37)	2.75 (2.09 to 3.65)
2555	77	130	850.4 (798.9 to 912.2)	1815.6 (1740.2 to 1886.7)	965.2 (870.8 to 1049.8)	2.14 (1.97 to 2.30)	1.91 (1.36 to 2.50)
2920	62	97	972.2 (911.8 to 1042.3)	2003.7 (1918.9 to 2090.4)	1031.4 (924.5 to 1130.0)	2.06 (1.90 to 2.21)	1.78 (1.35 to 2.50)
3285	38	74	1071.0 (1004.8 to 1144.7)	2189.7 (2085.0 to 2282.8)	1118.7 (991.7 to 1222.8)	2.04 (1.88 to 2.20)	2.24 (1.54 to 3.43)
3650	30	37	1176.9 (1099.2 to 1267.5)	2316.0 (2204.1 to 2413.9)	1139.2 (995.6 to 1264.9)	1.97 (1.80 to 2.13)	1.43 (0.87 to 2.31)
4015	16	25	1264.9 (1175.1 to 1364.5)	2451.4 (2322.6 to 2563.6)	1186.5 (1019.2 to 1328.2)	1.94 (1.75 to 2.11)	1.82 (0.97 to 3.70)
4380	6	13	1318.4 (1219.5 to 1437.2)	2581.9 (2444.3 to 2724.0)	1263.6 (1088.0 to 1444.4)	1.96 (1.78 to 2.17)	***
4745	5	6	1425.0 (1292.9 to 1587.6)	2747.8 (2537.3 to 2967.5)	1322.8 (1074.7 to 1565.5)	1.93 (1.71 to 2.18)	***
5110	0	1	1425.0 (1292.9 to 1587.6)	2881.8 (2585.8 to 3239.2)	1456.9 (1128.8 to 1814.1)	2.02 (1.76 to 2.34)	***

*** IRR/RR was not estimated when there were fewer than 20 events in the risk period or fewer than 5 events in one group. Confidence intervals obtained through percentile bootstrap.

Supplementary Table 5. Estimated risk of death by cause-specific (defined by ICD-10 chapters) comparing treated Tuberculosis cases to non-exposed controls.

	Number of events		Risk per 100,000 persons				
Time - Days	Unexposed	Exposed	Unexposed	Exposed	Risk difference per 100,000 - 95% CI	Risk ratio - 95% CI	Incidence rate ratio - 95% CI
				**Cause: All-cause**			
30	49	158	44.1 (32.8 to 56.7)	142.0 (121.3 to 161.8)	98.0 (74.1 to 120.3)	3.22 (2.41 to 4.31)	3.23 (2.41 to 4.32)
90	92	359	128.2 (108.3 to 150.4)	470.9 (427.7 to 511.8)	342.7 (294.5 to 386.2)	3.67 (3.07 to 4.43)	3.91 (3.11 to 4.91)
180	138	469	258.3 (229.1 to 290.2)	913.7 (854.6 to 967.4)	655.3 (597.9 to 716.0)	3.54 (3.13 to 4.06)	3.42 (2.90 to 4.12)
365	245	843	503.6 (460.2 to 546.1)	1756.2 (1668.9 to 1842.7)	1252.6 (1158.8 to 1347.0)	3.49 (3.17 to 3.87)	3.47 (3.04 to 4.06)
730	464	1345	1025.6 (956.9 to 1086.8)	3267.4 (3151.0 to 3379.0)	2241.8 (2105.9 to 2375.9)	3.19 (2.97 to 3.42)	2.95 (2.65 to 3.28)
1095	418	1068	1582.4 (1495.5 to 1663.5)	4686.3 (4548.7 to 4818.9)	3103.9 (2957.6 to 3264.4)	2.96 (2.80 to 3.14)	2.62 (2.35 to 2.90)
1460	370	928	2171.2 (2078.3 to 2275.3)	6169.0 (6005.2 to 6331.0)	3997.8 (3808.9 to 4182.0)	2.84 (2.69 to 2.97)	2.60 (2.32 to 2.90)
1825	305	656	2761.7 (2645.9 to 2883.9)	7440.4 (7247.8 to 7633.6)	4678.7 (4450.2 to 4899.8)	2.69 (2.56 to 2.83)	2.24 (1.97 to 2.58)
2190	233	561	3303.3 (3169.0 to 3432.1)	8747.2 (8554.4 to 8949.2)	5443.9 (5192.1 to 5684.4)	2.65 (2.53 to 2.77)	2.53 (2.21 to 2.92)
2555	187	403	3834.6 (3679.7 to 3993.0)	9898.0 (9659.9 to 10131.3)	6063.4 (5797.0 to 6358.7)	2.58 (2.46 to 2.70)	2.29 (1.96 to 2.72)
2920	166	330	4445.2 (4270.5 to 4628.4)	11107.0 (10821.8 to 11378.1)	6661.9 (6330.5 to 6986.4)	2.50 (2.38 to 2.62)	2.12 (1.75 to 2.52)
3285	122	252	5044.4 (4832.8 to 5269.4)	12325.4 (12002.8 to 12650.1)	7281.1 (6911.8 to 7650.4)	2.44 (2.33 to 2.55)	2.22 (1.77 to 2.76)
3650	75	153	5565.2 (5339.6 to 5810.5)	13399.1 (13044.5 to 13774.2)	7834.0 (7391.9 to 8265.8)	2.41 (2.29 to 2.52)	2.20 (1.71 to 2.94)
4015	53	99	6223.0 (5966.3 to 6519.9)	14560.7 (14139.1 to 14972.5)	8337.7 (7868.0 to 8784.3)	2.34 (2.22 to 2.45)	2.03 (1.50 to 2.81)
4380	23	46	6740.7 (6404.0 to 7126.0)	15629.6 (15115.7 to 16183.8)	8888.9 (8263.3 to 9508.4)	2.32 (2.17 to 2.47)	2.19 (1.33 to 3.89)
4745	12	6	7647.8 (7022.3 to 8337.2)	16073.9 (15424.5 to 16759.3)	8426.1 (7387.4 to 9407.7)	2.10 (1.91 to 2.33)	***
5110	2	1	8115.6 (7263.9 to 9035.6)	16322.2 (15575.6 to 17125.4)	8206.6 (7131.3 to 9453.2)	2.01 (1.81 to 2.28)	***
				**Cause: Natural - excluding (HIV/TB and external causes)**			
30	39	106	35.1 (24.3 to 46.8)	95.3 (78.2 to 112.3)	60.2 (39.1 to 80.0)	2.72 (1.94 to 3.92)	2.72 (1.94 to 3.93)
90	65	240	94.5 (76.3 to 113.5)	315.1 (279.4 to 346.6)	220.6 (181.1 to 258.9)	3.33 (2.69 to 4.27)	3.70 (2.88 to 4.91)
180	104	299	192.6 (167.0 to 220.3)	597.3 (547.6 to 639.5)	404.7 (353.1 to 453.4)	3.10 (2.70 to 3.63)	2.89 (2.37 to 3.59)
365	172	561	364.7 (327.1 to 400.4)	1158.2 (1090.1 to 1225.0)	793.5 (719.7 to 870.4)	3.18 (2.83 to 3.55)	3.29 (2.79 to 3.95)
730	341	880	748.7 (687.0 to 801.3)	2149.1 (2055.1 to 2234.0)	1400.4 (1296.8 to 1500.8)	2.87 (2.64 to 3.11)	2.63 (2.33 to 2.99)
1095	320	739	1174.2 (1099.4 to 1245.3)	3130.3 (3013.3 to 3241.7)	1956.1 (1840.5 to 2077.5)	2.67 (2.50 to 2.86)	2.37 (2.08 to 2.69)
1460	294	641	1641.7 (1560.6 to 1729.1)	4154.7 (4019.1 to 4294.0)	2513.0 (2358.5 to 2662.9)	2.53 (2.38 to 2.68)	2.26 (2.00 to 2.57)
1825	243	484	2111.8 (2005.8 to 2213.4)	5093.1 (4943.8 to 5257.1)	2981.3 (2800.1 to 3177.8)	2.41 (2.29 to 2.55)	2.08 (1.81 to 2.46)

2190	174	401	2515.5 (2392.7 to 2641.7)	6028.7 (5862.6 to 6198.2)	3513.2 (3301.6 to 3722.4)	2.40 (2.28 to 2.52)	2.42 (2.05 to 2.94)
2555	144	300	2924.5 (2792.8 to 3050.9)	6884.3 (6682.8 to 7094.9)	3959.8 (3729.5 to 4201.2)	2.35 (2.23 to 2.49)	2.21 (1.84 to 2.71)
2920	129	246	3399.0 (3237.5 to 3555.3)	7786.7 (7554.2 to 8016.9)	4387.7 (4111.8 to 4662.4)	2.29 (2.17 to 2.42)	2.04 (1.65 to 2.45)
3285	96	191	3868.7 (3689.3 to 4060.5)	8711.1 (8449.3 to 8984.4)	4842.3 (4541.5 to 5156.1)	2.25 (2.13 to 2.38)	2.14 (1.63 to 2.80)
3650	60	127	4283.7 (4080.3 to 4510.5)	9607.3 (9290.8 to 9930.4)	5323.6 (4961.2 to 5662.3)	2.24 (2.12 to 2.37)	2.29 (1.74 to 3.04)
4015	42	78	4805.9 (4583.8 to 5068.6)	10531.5 (10156.2 to 10890.4)	5725.5 (5322.3 to 6096.3)	2.19 (2.07 to 2.32)	2.02 (1.44 to 2.82)
4380	21	38	5280.5 (4946.7 to 5651.8)	11378.7 (10947.0 to 11845.7)	6098.2 (5565.6 to 6600.4)	2.15 (2.01 to 2.31)	1.98 (1.22 to 3.55)
4745	12	5	6187.6 (5585.3 to 6828.4)	11756.4 (11220.3 to 12389.8)	5568.8 (4687.4 to 6410.8)	1.90 (1.70 to 2.15)	***
5110	2	0	6655.3 (5826.3 to 7501.2)	11756.4 (11220.3 to 12389.8)	5101.0 (4105.4 to 6128.4)	1.77 (1.55 to 2.03)	***
**Cause: Cancer**							
30	7	29	6.3 (2.7 to 11.7)	26.1 (17.1 to 36.0)	19.8 (9.8 to 31.9)	4.13 (2.09 to 12.00)	4.14 (2.09 to 12.01)
90	8	71	13.6 (6.4 to 20.0)	91.1 (72.9 to 108.6)	77.5 (58.4 to 97.2)	6.69 (4.31 to 13.58)	8.89 (4.96 to 24.06)
180	18	73	30.6 (19.6 to 41.3)	159.9 (136.1 to 184.5)	129.4 (104.8 to 155.8)	5.23 (3.79 to 7.96)	4.08 (2.61 to 7.68)
365	38	123	68.6 (52.8 to 84.5)	283.0 (249.3 to 315.6)	214.5 (177.3 to 250.8)	4.13 (3.21 to 5.53)	3.27 (2.38 to 5.03)
730	60	185	136.4 (113.8 to 158.1)	490.9 (447.5 to 534.2)	354.5 (302.7 to 404.8)	3.60 (3.00 to 4.44)	3.14 (2.36 to 4.47)
1095	65	135	222.5 (189.4 to 252.1)	669.3 (613.6 to 722.9)	446.8 (389.4 to 507.3)	3.01 (2.63 to 3.63)	2.13 (1.66 to 2.82)
1460	64	120	325.1 (283.5 to 362.9)	861.8 (796.1 to 925.0)	536.7 (468.8 to 608.5)	2.65 (2.33 to 3.07)	1.94 (1.46 to 2.63)
1825	42	93	405.3 (359.5 to 452.8)	1040.6 (971.6 to 1111.0)	635.3 (554.4 to 715.7)	2.57 (2.27 to 2.94)	2.31 (1.67 to 3.25)
2190	28	70	469.4 (417.4 to 522.6)	1203.3 (1126.1 to 1285.3)	733.8 (641.0 to 830.0)	2.56 (2.27 to 2.91)	2.63 (1.81 to 4.26)
2555	21	54	529.9 (471.5 to 589.4)	1357.9 (1270.2 to 1449.7)	828.0 (719.3 to 938.9)	2.56 (2.26 to 2.91)	2.73 (1.74 to 4.91)
2920	30	43	639.3 (571.9 to 709.9)	1516.1 (1421.6 to 1615.6)	876.7 (753.5 to 998.8)	2.37 (2.09 to 2.69)	1.53 (0.96 to 2.62)
3285	20	35	736.0 (654.6 to 814.1)	1685.3 (1572.2 to 1807.3)	949.3 (796.1 to 1099.4)	2.29 (2.00 to 2.65)	1.88 (1.08 to 3.58)
3650	17	27	853.2 (752.8 to 955.2)	1872.8 (1735.8 to 1999.2)	1019.6 (855.2 to 1182.5)	2.20 (1.91 to 2.53)	1.72 (0.95 to 3.30)
4015	12	10	994.5 (869.9 to 1122.1)	1991.8 (1842.7 to 2146.2)	997.2 (798.4 to 1191.9)	2.00 (1.73 to 2.33)	0.91 (0.36 to 2.37)
4380	3	9	1057.1 (915.5 to 1207.1)	2185.7 (1989.6 to 2402.7)	1128.6 (877.1 to 1395.0)	2.07 (1.76 to 2.48)	***
4745	3	1	1298.8 (1020.8 to 1581.2)	2247.4 (2014.6 to 2491.0)	948.6 (571.5 to 1347.0)	1.73 (1.38 to 2.24)	***
5110	0	0	1298.8 (1020.8 to 1581.2)	2247.4 (2014.6 to 2491.0)	948.6 (571.5 to 1347.0)	1.73 (1.38 to 2.24)	***
**Cause: Cardiovascular**							
30	11	19	9.9 (4.5 to 16.2)	17.1 (9.9 to 25.6)	7.2 (-2.7 to 17.6)	1.73 (0.81 to 4.25)	1.73 (0.81 to 4.26)
90	28	44	35.5 (24.5 to 47.2)	57.3 (44.6 to 72.7)	21.9 (5.1 to 40.9)	1.62 (1.12 to 2.53)	1.57 (1.03 to 2.58)
180	41	54	74.1 (59.2 to 88.7)	108.3 (88.7 to 128.8)	34.1 (12.8 to 59.9)	1.46 (1.16 to 1.96)	1.32 (0.91 to 1.98)
365	45	115	119.1 (100.0 to 140.7)	223.3 (192.0 to 255.1)	104.2 (73.0 to 139.4)	1.87 (1.56 to 2.32)	2.58 (1.90 to 3.65)
730	115	191	248.2 (216.1 to 280.6)	439.1 (395.3 to 482.7)	190.8 (136.6 to 246.9)	1.77 (1.51 to 2.12)	1.69 (1.33 to 2.11)
1095	108	162	392.8 (349.7 to 433.0)	655.4 (607.0 to 710.9)	262.6 (199.4 to 331.4)	1.67 (1.47 to 1.92)	1.54 (1.26 to 1.94)
1460	95	146	543.9 (495.9 to 597.5)	888.8 (823.8 to 957.8)	344.9 (261.7 to 417.4)	1.63 (1.45 to 1.83)	1.59 (1.27 to 2.06)

1825	74	98	686.5 (627.3 to 744.6)	1079.6 (1006.7 to 1150.0)	393.1 (306.5 to 481.8)	1.57 (1.42 to 1.75)	1.38 (1.04 to 1.93)
2190	61	98	828.0 (758.4 to 893.8)	1307.6 (1231.6 to 1393.2)	479.6 (383.2 to 590.5)	1.58 (1.43 to 1.76)	1.69 (1.26 to 2.30)
2555	55	71	983.8 (903.7 to 1070.2)	1509.1 (1416.0 to 1609.6)	525.3 (410.1 to 652.9)	1.53 (1.39 to 1.71)	1.37 (0.97 to 1.94)
2920	32	47	1102.6 (1009.9 to 1196.4)	1680.2 (1569.9 to 1798.3)	577.6 (435.4 to 719.8)	1.52 (1.37 to 1.70)	1.57 (0.99 to 2.63)
3285	44	45	1320.8 (1210.7 to 1440.9)	1899.2 (1781.8 to 2027.1)	578.5 (407.0 to 734.9)	1.44 (1.29 to 1.59)	1.10 (0.71 to 1.68)
3650	19	24	1451.7 (1321.0 to 1581.8)	2068.2 (1929.7 to 2217.6)	616.4 (425.4 to 809.9)	1.42 (1.27 to 1.60)	1.37 (0.74 to 2.91)
4015	17	23	1675.1 (1502.8 to 1856.8)	2348.5 (2156.9 to 2528.7)	673.4 (437.0 to 907.3)	1.40 (1.24 to 1.59)	1.47 (0.78 to 2.74)
4380	7	9	1851.5 (1656.6 to 2087.1)	2547.1 (2347.5 to 2776.2)	695.6 (407.3 to 992.3)	1.38 (1.20 to 1.59)	***
4745	4	1	2165.0 (1833.4 to 2553.2)	2621.3 (2393.1 to 2881.6)	456.2 (4.9 to 920.3)	1.21 (1.00 to 1.49)	***
5110	0	0	2165.0 (1833.4 to 2553.2)	2621.3 (2393.1 to 2881.6)	456.2 (4.9 to 920.3)	1.21 (1.00 to 1.49)	***
				**Cause: Endocrine**			
30	3	8	2.7 (0.0 to 6.3)	7.2 (2.7 to 12.6)	4.5 (-1.8 to 10.8)	***	***
90	5	11	7.3 (2.7 to 12.8)	17.3 (10.0 to 25.4)	10.0 (0.9 to 19.2)	2.37 (1.10 to 6.39)	***
180	10	24	16.8 (9.3 to 24.3)	40.0 (28.8 to 53.1)	23.2 (8.3 to 38.0)	2.38 (1.36 to 4.47)	2.41 (1.21 to 6.28)
365	16	37	32.5 (22.0 to 43.5)	77.0 (60.9 to 94.4)	44.4 (24.0 to 64.3)	2.37 (1.61 to 3.66)	2.33 (1.37 to 4.49)
730	28	68	64.1 (49.0 to 80.0)	154.0 (128.8 to 180.2)	89.9 (61.8 to 121.6)	2.40 (1.81 to 3.25)	2.47 (1.67 to 3.97)
1095	24	63	95.9 (76.7 to 114.3)	237.8 (207.0 to 273.0)	141.8 (105.2 to 180.9)	2.48 (1.96 to 3.19)	2.70 (1.78 to 4.38)
1460	22	49	130.5 (105.0 to 154.7)	316.1 (277.7 to 356.6)	185.5 (142.4 to 231.3)	2.42 (1.95 to 3.12)	2.31 (1.42 to 3.93)
1825	20	46	169.8 (139.9 to 200.7)	406.7 (362.4 to 456.3)	236.8 (182.8 to 293.9)	2.39 (1.96 to 3.02)	2.40 (1.49 to 4.41)
2190	12	38	197.6 (165.2 to 231.8)	496.5 (444.4 to 552.7)	298.9 (237.3 to 364.2)	2.51 (2.07 to 3.11)	3.33 (1.75 to 6.83)
2555	11	23	228.0 (192.1 to 268.5)	562.9 (502.7 to 625.3)	334.9 (263.6 to 407.5)	2.47 (2.05 to 2.99)	2.22 (1.06 to 5.08)
2920	8	20	257.3 (215.6 to 304.4)	637.2 (562.8 to 706.7)	379.9 (292.4 to 460.4)	2.48 (2.01 to 3.03)	2.67 (1.22 to 7.26)
3285	5	21	281.4 (235.3 to 331.7)	737.0 (657.7 to 820.8)	455.6 (357.7 to 551.6)	2.62 (2.12 to 3.24)	4.51 (2.03 to 20.50)
3650	3	9	301.7 (251.3 to 354.2)	797.9 (703.2 to 890.7)	496.2 (386.8 to 597.0)	2.64 (2.16 to 3.26)	***
4015	1	6	314.3 (257.4 to 371.8)	861.2 (757.0 to 966.1)	547.0 (423.4 to 654.4)	2.74 (2.21 to 3.40)	***
4380	1	5	344.3 (269.0 to 436.9)	972.1 (828.5 to 1115.1)	627.8 (461.3 to 792.1)	2.82 (2.10 to 3.74)	***
4745	1	0	450.3 (290.6 to 699.6)	972.1 (828.5 to 1115.1)	521.8 (244.6 to 737.3)	2.16 (1.36 to 3.42)	***
5110	0	0	450.3 (290.6 to 699.6)	972.1 (828.5 to 1115.1)	521.8 (244.6 to 737.3)	2.16 (1.36 to 3.42)	***
				**Cause: Respiratory**			
30	4	23	3.6 (0.0 to 7.2)	20.7 (11.7 to 29.2)	17.1 (8.5 to 26.5)	***	***
90	5	44	8.2 (3.6 to 13.6)	61.0 (46.4 to 75.2)	52.9 (37.0 to 67.9)	7.48 (4.25 to 17.87)	8.82 (4.12 to 44.10)
180	14	66	21.4 (13.8 to 30.3)	123.4 (103.8 to 141.4)	102.0 (79.5 to 121.9)	5.77 (3.81 to 8.70)	4.74 (2.72 to 9.31)
365	17	113	38.4 (26.9 to 49.6)	236.2 (205.4 to 261.1)	197.8 (166.9 to 223.7)	6.15 (4.71 to 8.78)	6.71 (4.37 to 11.98)
730	39	181	82.9 (65.3 to 100.3)	440.5 (400.5 to 481.3)	357.6 (316.0 to 400.3)	5.31 (4.25 to 6.72)	4.72 (3.46 to 6.80)
1095	37	148	131.8 (108.1 to 157.1)	637.9 (588.2 to 685.4)	506.1 (451.3 to 557.6)	4.84 (3.98 to 5.87)	4.11 (2.89 to 5.99)

1460	25	134	171.6 (145.0 to 202.1)	852.4 (789.1 to 912.0)	680.7 (610.4 to 747.1)	4.97 (4.12 to 6.08)	5.55 (3.70 to 9.00)
1825	35	86	239.3 (204.9 to 278.7)	1020.1 (950.0 to 1091.5)	780.8 (705.7 to 858.2)	4.26 (3.64 to 5.11)	2.56 (1.84 to 3.88)
2190	21	65	289.0 (248.0 to 335.3)	1171.2 (1092.2 to 1252.7)	882.2 (798.0 to 976.4)	4.05 (3.48 to 4.80)	3.26 (2.06 to 6.00)
2555	21	55	349.6 (297.1 to 407.5)	1328.5 (1249.0 to 1422.9)	979.0 (887.0 to 1082.8)	3.80 (3.27 to 4.51)	2.78 (1.73 to 4.96)
2920	16	53	407.9 (347.7 to 472.6)	1523.7 (1436.6 to 1638.4)	1115.7 (1008.7 to 1239.7)	3.73 (3.22 to 4.41)	3.54 (2.14 to 6.76)
3285	6	35	437.5 (375.8 to 504.5)	1693.9 (1580.1 to 1821.0)	1256.4 (1130.0 to 1389.2)	3.87 (3.30 to 4.52)	6.27 (2.96 to 21.03)
3650	4	25	469.6 (401.8 to 544.3)	1876.8 (1720.1 to 2023.5)	1407.2 (1241.7 to 1561.3)	4.00 (3.36 to 4.68)	***
4015	4	14	520.9 (445.0 to 612.7)	2039.6 (1872.0 to 2215.4)	1518.7 (1306.8 to 1701.8)	3.92 (3.21 to 4.64)	***
4380	2	7	559.0 (471.2 to 673.3)	2205.1 (2012.0 to 2416.6)	1646.1 (1398.7 to 1864.8)	3.94 (3.18 to 4.78)	***
4745	1	2	612.3 (487.7 to 762.6)	2388.5 (2061.0 to 2821.0)	1776.3 (1411.0 to 2163.4)	3.90 (2.88 to 5.07)	***
5110	0	0	612.3 (487.7 to 762.6)	2388.5 (2061.0 to 2821.0)	1776.3 (1411.0 to 2163.4)	3.90 (2.88 to 5.07)	***
				**Cause: External**			
30	10	21	9.0 (4.0 to 15.3)	18.9 (10.8 to 27.8)	9.9 (-0.5 to 18.9)	2.10 (0.97 to 4.89)	2.10 (0.97 to 4.91)
90	25	49	31.8 (22.7 to 42.8)	63.8 (48.7 to 78.9)	31.9 (14.8 to 51.2)	2.00 (1.37 to 3.06)	1.96 (1.24 to 3.23)
180	30	74	60.1 (46.2 to 76.0)	133.8 (112.9 to 157.6)	73.7 (47.4 to 101.1)	2.23 (1.68 to 3.02)	2.48 (1.67 to 3.95)
365	68	128	128.4 (107.4 to 151.2)	261.9 (232.4 to 295.5)	133.5 (97.7 to 172.4)	2.04 (1.66 to 2.53)	1.90 (1.44 to 2.70)
730	115	240	257.4 (227.1 to 288.0)	531.2 (484.9 to 577.2)	273.7 (218.6 to 331.5)	2.06 (1.79 to 2.40)	2.12 (1.71 to 2.67)
1095	95	168	384.6 (345.0 to 426.8)	754.9 (697.5 to 816.4)	370.4 (301.3 to 444.5)	1.96 (1.73 to 2.24)	1.82 (1.42 to 2.36)
1460	67	152	491.6 (449.1 to 539.8)	998.3 (931.7 to 1063.0)	506.6 (425.7 to 587.5)	2.03 (1.81 to 2.27)	2.35 (1.76 to 3.09)
1825	60	110	608.1 (559.6 to 666.8)	1211.6 (1137.9 to 1295.9)	603.4 (516.5 to 692.1)	1.99 (1.79 to 2.22)	1.91 (1.36 to 2.64)
2190	48	93	720.2 (658.2 to 792.1)	1428.1 (1347.7 to 1527.2)	707.8 (607.0 to 809.3)	1.98 (1.79 to 2.20)	2.04 (1.44 to 2.83)
2555	40	62	834.6 (758.4 to 911.7)	1605.7 (1512.3 to 1708.4)	771.1 (666.3 to 891.0)	1.92 (1.74 to 2.15)	1.64 (1.19 to 2.61)
2920	36	49	966.5 (890.3 to 1053.7)	1783.5 (1684.4 to 1895.8)	817.0 (692.9 to 952.5)	1.85 (1.67 to 2.05)	1.45 (0.99 to 2.22)
3285	22	33	1075.8 (985.7 to 1177.8)	1943.7 (1830.9 to 2067.9)	867.9 (727.0 to 1033.8)	1.81 (1.63 to 2.02)	1.61 (0.96 to 2.72)
3650	13	13	1168.6 (1067.8 to 1282.1)	2032.7 (1911.3 to 2171.5)	864.1 (707.5 to 1031.9)	1.74 (1.57 to 1.95)	1.08 (0.47 to 2.52)
4015	8	15	1268.1 (1133.6 to 1401.8)	2199.0 (2047.7 to 2355.6)	930.8 (726.3 to 1121.9)	1.73 (1.53 to 1.98)	***
4380	1	6	1294.4 (1156.0 to 1438.1)	2365.7 (2156.5 to 2560.4)	1071.3 (811.1 to 1313.6)	1.83 (1.58 to 2.13)	***
4745	0	0	1294.4 (1156.0 to 1438.1)	2365.7 (2156.5 to 2560.4)	1071.3 (811.1 to 1313.6)	1.83 (1.58 to 2.13)	***
5110	0	1	1294.4 (1156.0 to 1438.1)	2614.0 (2228.2 to 3204.4)	1319.6 (887.7 to 1926.2)	2.02 (1.62 to 2.48)	***

*** IRR/RR was not estimated when there were fewer than 20 events in the risk period or fewer than 5 events in one group. Confidence intervals obtained through percentile bootstrap.

Supplementary Table 6. Estimated risk of death by cause-specific (defined by ICD-10 chapters) comparing household Tuberculosis contacts to non-exposed controls.

	Number of events		Risk per 100,000				
Time - Days	Unexposed	Exposed	Unexposed	Exposed	Risk difference per 100,000 - 95% CI	Risk ratio - 95% CI	Incidence rate ratio - 95% CI
**Cause: All-cause**							
30	100	135	22.4 (18.1 to 27.3)	30.2 (24.9 to 35.2)	7.8 (1.4 to 14.1)	1.35 (1.06 to 1.76)	1.35 (1.06 to 1.76)
90	187	255	65.0 (57.4 to 72.6)	88.3 (80.3 to 97.2)	23.3 (11.5 to 34.6)	1.36 (1.16 to 1.58)	1.36 (1.13 to 1.64)
180	272	348	129.0 (117.8 to 140.3)	170.0 (157.1 to 182.6)	41.0 (23.8 to 56.5)	1.32 (1.17 to 1.47)	1.28 (1.08 to 1.52)
365	573	760	270.9 (254.8 to 286.2)	358.0 (339.6 to 374.7)	87.2 (63.8 to 109.7)	1.32 (1.23 to 1.43)	1.33 (1.19 to 1.48)
730	1056	1268	561.3 (537.8 to 586.2)	706.1 (680.8 to 730.5)	144.8 (112.3 to 180.9)	1.26 (1.20 to 1.33)	1.20 (1.11 to 1.32)
1095	891	1094	844.4 (814.9 to 875.4)	1052.8 (1019.9 to 1084.5)	208.5 (164.0 to 248.4)	1.25 (1.19 to 1.30)	1.23 (1.13 to 1.33)
1460	810	905	1143.2 (1107.6 to 1180.9)	1385.9 (1347.8 to 1425.0)	242.8 (189.6 to 294.9)	1.21 (1.16 to 1.26)	1.12 (1.03 to 1.22)
1825	713	793	1452.3 (1414.0 to 1498.3)	1728.3 (1676.8 to 1777.4)	276.0 (209.9 to 332.2)	1.19 (1.14 to 1.23)	1.12 (1.01 to 1.25)
2190	568	690	1746.4 (1700.6 to 1799.4)	2083.7 (2026.6 to 2138.3)	337.3 (266.7 to 408.4)	1.19 (1.15 to 1.24)	1.22 (1.10 to 1.36)
2555	502	557	2061.7 (2005.5 to 2122.1)	2436.4 (2372.0 to 2502.2)	374.7 (296.8 to 459.0)	1.18 (1.14 to 1.22)	1.11 (0.99 to 1.25)
2920	426	449	2400.5 (2337.7 to 2463.1)	2793.9 (2722.4 to 2865.8)	393.3 (305.0 to 485.5)	1.16 (1.12 to 1.21)	1.06 (0.93 to 1.20)
3285	315	365	2724.1 (2651.7 to 2796.5)	3171.0 (3095.3 to 3245.7)	446.9 (343.9 to 553.2)	1.16 (1.12 to 1.21)	1.16 (1.00 to 1.33)
3650	276	278	3119.4 (3029.4 to 3204.1)	3562.0 (3472.7 to 3653.5)	442.6 (325.3 to 566.0)	1.14 (1.10 to 1.19)	1.01 (0.84 to 1.21)
4015	168	192	3478.5 (3371.0 to 3578.6)	3970.6 (3866.9 to 4079.0)	492.1 (345.3 to 643.0)	1.14 (1.10 to 1.19)	1.15 (0.93 to 1.42)
4380	97	116	3853.2 (3703.2 to 3978.6)	4437.3 (4304.6 to 4571.3)	584.1 (399.4 to 769.6)	1.15 (1.10 to 1.21)	1.20 (0.91 to 1.63)
4745	35	31	4203.3 (4015.2 to 4402.6)	4728.3 (4554.1 to 4918.2)	525.0 (270.2 to 784.6)	1.12 (1.06 to 1.19)	0.89 (0.52 to 1.47)
5110	15	14	4662.0 (4375.8 to 4986.3)	5116.8 (4817.1 to 5410.1)	454.8 (2.3 to 862.5)	1.10 (1.00 to 1.19)	0.94 (0.37 to 1.92)
**Cause: Natural - excluding (HIV/TB and external causes)**							
30	70	88	15.7 (12.3 to 19.9)	19.7 (15.6 to 23.9)	4.0 (-2.0 to 9.2)	1.26 (0.89 to 1.67)	1.26 (0.89 to 1.67)
90	119	159	42.8 (36.7 to 49.4)	55.9 (49.2 to 62.6)	13.1 (3.9 to 22.1)	1.31 (1.08 to 1.59)	1.34 (1.04 to 1.72)
180	201	233	90.1 (80.9 to 99.4)	110.6 (100.6 to 120.0)	20.6 (6.8 to 33.5)	1.23 (1.07 to 1.41)	1.16 (0.95 to 1.42)
365	396	488	188.2 (174.1 to 201.1)	231.4 (217.6 to 245.9)	43.2 (25.3 to 61.7)	1.23 (1.13 to 1.35)	1.23 (1.09 to 1.41)
730	738	830	391.2 (371.3 to 411.2)	459.0 (438.8 to 479.9)	67.9 (39.1 to 96.0)	1.17 (1.10 to 1.26)	1.13 (1.01 to 1.25)
1095	630	699	591.2 (567.2 to 615.6)	680.8 (654.4 to 708.7)	89.6 (53.2 to 124.3)	1.15 (1.09 to 1.22)	1.11 (0.99 to 1.23)
1460	543	574	791.6 (762.8 to 821.7)	892.2 (859.6 to 923.2)	100.6 (56.3 to 140.4)	1.13 (1.07 to 1.18)	1.06 (0.95 to 1.18)
1825	487	506	1002.9 (970.3 to 1037.0)	1110.5 (1074.2 to 1147.2)	107.6 (57.6 to 153.4)	1.11 (1.06 to 1.16)	1.04 (0.93 to 1.19)
2190	390	432	1204.5 (1166.9 to 1244.1)	1332.9 (1289.3 to 1376.7)	128.4 (72.8 to 183.3)	1.11 (1.06 to 1.15)	1.11 (0.98 to 1.26)

2555	321	361	1406.0 (1360.2 to 1454.2)	1561.0 (1513.3 to 1613.0)	154.9 (90.3 to 218.3)	1.11 (1.06 to 1.16)	1.13 (0.98 to 1.32)
2920	285	293	1632.7 (1581.7 to 1679.5)	1793.2 (1735.8 to 1853.8)	160.5 (85.9 to 231.7)	1.10 (1.05 to 1.14)	1.03 (0.88 to 1.21)
3285	195	241	1832.5 (1776.0 to 1893.1)	2042.1 (1976.7 to 2102.2)	209.6 (126.1 to 286.3)	1.11 (1.07 to 1.16)	1.24 (1.01 to 1.48)
3650	185	164	2099.7 (2035.9 to 2170.8)	2272.7 (2198.6 to 2337.7)	173.1 (75.1 to 263.4)	1.08 (1.03 to 1.13)	0.89 (0.71 to 1.09)
4015	107	120	2326.6 (2247.9 to 2408.0)	2533.5 (2442.2 to 2613.1)	206.9 (86.5 to 325.9)	1.09 (1.04 to 1.14)	1.13 (0.87 to 1.47)
4380	56	82	2542.5 (2444.0 to 2642.1)	2859.8 (2747.0 to 2966.9)	317.2 (170.2 to 472.0)	1.12 (1.07 to 1.19)	1.47 (1.05 to 2.23)
4745	24	17	2800.9 (2668.4 to 2971.1)	3026.7 (2882.2 to 3178.6)	225.8 (16.2 to 414.0)	1.08 (1.01 to 1.15)	0.71 (0.38 to 1.27)
5110	10	7	3113.7 (2878.6 to 3382.7)	3245.0 (3017.8 to 3486.0)	131.3 (-227.8 to 449.0)	1.04 (0.93 to 1.15)	***
**Cause: Cancer**							
30	11	16	2.5 (1.1 to 4.0)	3.6 (1.8 to 5.4)	1.1 (-1.1 to 3.4)	1.45 (0.68 to 3.41)	1.45 (0.68 to 3.42)
90	25	27	8.2 (5.6 to 11.1)	9.7 (7.0 to 12.7)	1.6 (-1.9 to 5.4)	1.19 (0.81 to 1.87)	1.08 (0.63 to 1.84)
180	39	49	17.3 (13.7 to 21.8)	21.2 (17.5 to 25.3)	3.9 (-2.0 to 9.7)	1.22 (0.90 to 1.62)	1.26 (0.83 to 2.02)
365	58	86	31.7 (26.5 to 37.5)	42.5 (35.8 to 48.0)	10.8 (2.8 to 18.0)	1.34 (1.08 to 1.64)	1.48 (1.06 to 2.08)
730	159	147	75.5 (67.2 to 84.6)	82.7 (73.4 to 91.3)	7.2 (-5.3 to 19.9)	1.10 (0.94 to 1.28)	0.93 (0.72 to 1.18)
1095	133	122	117.8 (107.4 to 130.6)	121.3 (109.5 to 132.4)	3.5 (-12.5 to 19.3)	1.03 (0.90 to 1.18)	0.92 (0.71 to 1.14)
1460	117	109	161.0 (149.0 to 176.1)	161.6 (148.5 to 174.4)	0.6 (-17.5 to 19.7)	1.00 (0.89 to 1.13)	0.93 (0.73 to 1.19)
1825	87	100	198.6 (183.9 to 215.7)	204.5 (188.3 to 220.9)	5.9 (-16.6 to 30.3)	1.03 (0.92 to 1.16)	1.15 (0.89 to 1.58)
2190	86	77	243.0 (225.3 to 262.6)	244.6 (226.5 to 264.0)	1.6 (-25.7 to 27.7)	1.01 (0.90 to 1.12)	0.90 (0.68 to 1.23)
2555	60	71	280.8 (261.3 to 301.2)	289.8 (266.9 to 313.7)	9.1 (-20.0 to 42.1)	1.03 (0.93 to 1.16)	1.19 (0.89 to 1.64)
2920	48	48	318.9 (297.5 to 342.4)	327.6 (303.0 to 352.7)	8.7 (-27.2 to 43.1)	1.03 (0.92 to 1.14)	1.00 (0.66 to 1.47)
3285	33	41	352.9 (327.7 to 378.9)	370.0 (341.9 to 398.7)	17.2 (-21.6 to 58.1)	1.05 (0.94 to 1.17)	1.25 (0.79 to 2.03)
3650	27	26	391.2 (364.5 to 421.5)	406.8 (376.2 to 437.3)	15.6 (-29.5 to 60.1)	1.04 (0.93 to 1.16)	0.97 (0.55 to 1.59)
4015	20	27	432.3 (398.3 to 466.0)	464.6 (427.2 to 501.6)	32.3 (-20.2 to 82.3)	1.07 (0.96 to 1.20)	1.36 (0.79 to 2.53)
4380	15	17	488.5 (443.8 to 531.6)	530.5 (484.5 to 583.6)	42.0 (-21.1 to 116.6)	1.09 (0.96 to 1.25)	1.14 (0.56 to 2.80)
4745	3	2	508.9 (459.6 to 559.3)	547.2 (495.3 to 603.6)	38.2 (-36.8 to 118.5)	1.08 (0.93 to 1.24)	***
5110	3	2	604.4 (503.9 to 746.3)	623.3 (521.5 to 752.5)	18.9 (-142.9 to 178.3)	1.03 (0.80 to 1.34)	***
**Cause: Cardiovascular**							
30	17	29	3.8 (2.2 to 5.8)	6.5 (3.9 to 9.2)	2.7 (-0.2 to 5.6)	1.70 (0.95 to 3.30)	1.71 (0.95 to 3.31)
90	33	48	11.3 (8.4 to 15.0)	17.4 (13.4 to 21.3)	6.1 (1.1 to 11.0)	1.54 (1.08 to 2.22)	1.45 (0.91 to 2.26)
180	69	67	27.6 (22.9 to 32.7)	33.2 (27.2 to 38.2)	5.6 (-1.8 to 12.3)	1.20 (0.94 to 1.50)	0.97 (0.69 to 1.37)
365	137	162	61.5 (53.9 to 69.1)	73.2 (65.4 to 81.2)	11.7 (1.1 to 22.3)	1.19 (1.02 to 1.40)	1.18 (0.96 to 1.48)
730	246	260	129.2 (118.9 to 141.3)	144.4 (133.2 to 157.7)	15.2 (-1.5 to 31.9)	1.12 (0.99 to 1.26)	1.06 (0.89 to 1.25)
1095	195	235	191.4 (178.3 to 206.3)	219.4 (204.7 to 234.4)	28.0 (7.3 to 49.9)	1.15 (1.04 to 1.28)	1.21 (1.00 to 1.44)
1460	181	158	258.2 (241.4 to 277.0)	277.8 (259.3 to 296.6)	19.5 (-5.4 to 43.9)	1.08 (0.98 to 1.18)	0.88 (0.70 to 1.09)
1825	160	151	327.7 (308.8 to 348.8)	342.9 (321.2 to 364.8)	15.2 (-14.0 to 43.2)	1.05 (0.96 to 1.14)	0.95 (0.76 to 1.16)

2190	118	132	388.7 (368.5 to 410.4)	411.1 (387.4 to 437.7)	22.4 (-10.1 to 52.7)	1.06 (0.98 to 1.14)	1.12 (0.89 to 1.44)
2555	101	122	452.2 (429.6 to 479.0)	488.3 (462.4 to 518.3)	36.1 (1.6 to 72.6)	1.08 (1.00 to 1.17)	1.21 (0.92 to 1.57)
2920	98	99	529.9 (503.0 to 561.1)	566.6 (538.6 to 598.9)	36.7 (-3.9 to 75.3)	1.07 (0.99 to 1.15)	1.01 (0.79 to 1.36)
3285	74	82	606.0 (574.5 to 639.2)	651.0 (617.6 to 688.2)	45.0 (-4.3 to 90.1)	1.07 (0.99 to 1.15)	1.11 (0.82 to 1.53)
3650	63	57	698.7 (660.2 to 739.4)	729.6 (688.5 to 772.1)	30.9 (-28.5 to 85.8)	1.04 (0.96 to 1.13)	0.91 (0.62 to 1.27)
4015	41	32	786.6 (742.9 to 835.3)	798.9 (753.1 to 842.9)	12.3 (-53.9 to 74.1)	1.02 (0.93 to 1.10)	0.79 (0.44 to 1.30)
4380	20	26	864.2 (809.8 to 920.6)	900.7 (835.8 to 965.0)	36.4 (-42.6 to 117.6)	1.04 (0.95 to 1.14)	1.31 (0.75 to 2.62)
4745	10	4	982.6 (887.6 to 1084.4)	940.6 (868.6 to 1027.7)	-42.0 (-166.4 to 81.8)	0.96 (0.85 to 1.09)	***
5110	2	1	1027.2 (917.5 to 1143.3)	968.3 (876.2 to 1078.1)	-58.8 (-199.8 to 86.3)	0.94 (0.82 to 1.10)	***
**Cause: Endocrine**							
30	4	6	0.9 (0.2 to 1.8)	1.3 (0.4 to 2.5)	0.4 (-0.9 to 1.8)	***	***
90	10	4	3.2 (1.6 to 5.0)	2.3 (0.9 to 3.8)	-0.9 (-3.2 to 1.1)	***	***
180	12	16	6.0 (3.7 to 8.3)	6.0 (3.7 to 8.3)	0.0 (-3.2 to 3.3)	1.00 (0.55 to 1.83)	1.33 (0.59 to 3.00)
365	30	30	13.4 (10.1 to 17.0)	13.5 (10.1 to 17.2)	0.1 (-5.0 to 5.3)	1.00 (0.68 to 1.48)	1.00 (0.58 to 1.65)
730	44	49	25.5 (20.5 to 30.7)	26.8 (21.8 to 31.8)	1.3 (-6.4 to 8.0)	1.05 (0.78 to 1.36)	1.11 (0.73 to 1.69)
1095	44	41	39.4 (33.0 to 46.3)	39.9 (33.3 to 47.1)	0.4 (-8.7 to 9.9)	1.01 (0.79 to 1.28)	0.93 (0.60 to 1.49)
1460	32	40	51.2 (44.4 to 59.1)	54.6 (46.7 to 62.8)	3.4 (-7.6 to 14.2)	1.07 (0.87 to 1.31)	1.25 (0.79 to 2.00)
1825	40	31	68.5 (60.0 to 77.9)	68.0 (59.4 to 77.5)	-0.5 (-13.2 to 12.5)	0.99 (0.82 to 1.20)	0.78 (0.47 to 1.22)
2190	31	39	84.5 (73.6 to 95.7)	87.7 (76.8 to 99.6)	3.1 (-11.7 to 19.1)	1.04 (0.87 to 1.25)	1.26 (0.79 to 2.07)
2555	26	21	100.9 (88.3 to 113.4)	100.9 (88.9 to 113.3)	0.0 (-17.3 to 18.4)	1.00 (0.84 to 1.20)	0.81 (0.45 to 1.48)
2920	23	21	119.4 (104.3 to 134.7)	117.4 (103.9 to 132.9)	-2.0 (-21.9 to 19.4)	0.98 (0.83 to 1.18)	0.92 (0.47 to 1.70)
3285	20	10	139.9 (123.0 to 158.5)	127.5 (111.3 to 144.1)	-12.4 (-36.6 to 10.1)	0.91 (0.76 to 1.08)	0.50 (0.17 to 1.06)
3650	11	11	155.1 (135.5 to 175.3)	143.2 (125.0 to 161.8)	-11.9 (-38.8 to 13.5)	0.92 (0.77 to 1.10)	1.01 (0.39 to 2.35)
4015	4	11	163.6 (142.9 to 187.3)	166.0 (141.9 to 188.2)	2.3 (-30.9 to 32.1)	1.01 (0.82 to 1.21)	***
4380	3	5	176.9 (151.2 to 204.1)	187.5 (158.7 to 217.5)	10.6 (-27.3 to 48.0)	1.06 (0.86 to 1.31)	***
4745	1	0	186.6 (156.0 to 222.2)	187.5 (158.7 to 217.5)	0.8 (-43.2 to 43.5)	1.00 (0.80 to 1.27)	***
5110	4	0	332.4 (204.2 to 529.5)	187.5 (158.7 to 217.5)	-144.9 (-343.9 to -11.4)	0.56 (0.35 to 0.95)	***
**Cause: Respiratory**							
30	11	11	2.5 (1.1 to 3.9)	2.5 (1.1 to 3.9)	0.0 (-2.0 to 2.0)	1.00 (0.40 to 2.34)	1.00 (0.40 to 2.33)
90	19	23	6.8 (4.4 to 9.3)	7.7 (5.4 to 10.1)	0.9 (-2.7 to 4.4)	1.13 (0.71 to 1.89)	1.21 (0.65 to 2.52)
180	27	26	13.1 (9.7 to 16.8)	13.8 (10.6 to 17.5)	0.7 (-3.9 to 5.8)	1.05 (0.74 to 1.55)	0.96 (0.57 to 1.72)
365	52	60	26.0 (20.8 to 31.0)	28.7 (24.2 to 33.4)	2.7 (-4.4 to 9.6)	1.10 (0.85 to 1.47)	1.15 (0.78 to 1.72)
730	81	110	48.3 (41.6 to 55.0)	58.9 (51.7 to 65.7)	10.6 (0.3 to 22.3)	1.22 (1.01 to 1.51)	1.36 (1.01 to 1.84)
1095	73	80	71.3 (62.8 to 80.9)	84.1 (76.2 to 93.2)	12.8 (-0.0 to 26.7)	1.18 (1.00 to 1.41)	1.10 (0.84 to 1.49)
1460	52	89	90.5 (81.1 to 100.8)	116.7 (106.3 to 128.2)	26.2 (11.8 to 41.2)	1.29 (1.12 to 1.49)	1.72 (1.24 to 2.41)

1825	59	63	116.3 (104.5 to 128.6)	143.7 (132.0 to 157.2)	27.4 (11.4 to 44.0)	1.24 (1.09 to 1.42)	1.07 (0.75 to 1.54)
2190	46	55	140.1 (128.2 to 154.6)	171.9 (157.1 to 188.7)	31.8 (11.5 to 50.9)	1.23 (1.08 to 1.38)	1.20 (0.83 to 1.82)
2555	33	44	160.6 (146.5 to 176.2)	199.6 (183.9 to 216.8)	39.1 (17.0 to 60.7)	1.24 (1.10 to 1.41)	1.34 (0.87 to 2.10)
2920	22	39	178.2 (161.4 to 195.8)	230.6 (212.1 to 250.8)	52.4 (29.8 to 77.5)	1.29 (1.15 to 1.46)	1.78 (1.07 to 3.29)
3285	19	43	197.4 (177.1 to 216.9)	275.0 (252.2 to 298.2)	77.6 (48.7 to 108.2)	1.39 (1.24 to 1.59)	2.27 (1.38 to 3.99)
3650	29	26	239.4 (216.7 to 264.4)	311.5 (285.5 to 337.8)	72.1 (35.7 to 106.8)	1.30 (1.14 to 1.48)	0.90 (0.52 to 1.47)
4015	10	12	260.4 (233.9 to 288.3)	338.2 (308.5 to 367.3)	77.8 (37.5 to 115.1)	1.30 (1.13 to 1.48)	1.21 (0.55 to 3.58)
4380	3	7	272.1 (242.8 to 303.5)	366.5 (332.5 to 400.8)	94.4 (48.8 to 139.8)	1.35 (1.16 to 1.55)	***
4745	2	6	291.4 (255.9 to 336.7)	426.2 (368.3 to 491.4)	134.9 (65.3 to 211.6)	1.46 (1.21 to 1.79)	***
5110	0	0	291.4 (255.9 to 336.7)	426.2 (368.3 to 491.4)	134.9 (65.3 to 211.6)	1.46 (1.21 to 1.79)	***
				**Cause: External**			
30	26	34	5.8 (3.8 to 8.3)	7.6 (5.0 to 10.4)	1.8 (-1.7 to 5.3)	1.31 (0.78 to 2.29)	1.31 (0.78 to 2.29)
90	62	81	20.0 (16.3 to 24.5)	26.1 (21.2 to 31.5)	6.1 (-0.4 to 12.0)	1.31 (0.98 to 1.70)	1.31 (0.93 to 1.87)
180	66	99	35.5 (30.3 to 42.0)	49.3 (42.7 to 55.9)	13.8 (4.6 to 22.4)	1.39 (1.11 to 1.70)	1.50 (1.11 to 2.10)
365	162	248	75.5 (67.2 to 84.9)	110.7 (100.8 to 121.1)	35.1 (20.7 to 47.7)	1.47 (1.26 to 1.69)	1.53 (1.28 to 1.88)
730	297	378	157.2 (144.1 to 169.3)	214.6 (200.1 to 229.5)	57.4 (37.7 to 76.6)	1.37 (1.22 to 1.51)	1.27 (1.09 to 1.50)
1095	244	343	234.8 (218.9 to 250.9)	323.1 (305.1 to 341.3)	88.4 (62.6 to 112.7)	1.38 (1.26 to 1.50)	1.41 (1.21 to 1.65)
1460	254	300	328.5 (309.8 to 347.2)	433.3 (411.9 to 457.4)	104.8 (75.6 to 138.8)	1.32 (1.22 to 1.44)	1.18 (1.00 to 1.40)
1825	212	253	420.2 (398.1 to 445.9)	542.8 (515.1 to 571.4)	122.6 (88.0 to 157.2)	1.29 (1.20 to 1.39)	1.20 (0.99 to 1.42)
2190	172	227	509.7 (484.0 to 536.0)	659.8 (630.4 to 693.2)	150.2 (112.5 to 188.7)	1.29 (1.21 to 1.38)	1.32 (1.10 to 1.62)
2555	173	180	618.6 (588.9 to 649.4)	774.3 (738.4 to 810.9)	155.6 (106.6 to 201.7)	1.25 (1.17 to 1.34)	1.04 (0.85 to 1.28)
2920	136	138	726.7 (691.6 to 762.0)	884.5 (846.3 to 928.6)	157.7 (104.2 to 211.1)	1.22 (1.14 to 1.30)	1.02 (0.82 to 1.25)
3285	118	119	848.5 (807.3 to 888.2)	1007.5 (957.2 to 1053.8)	159.0 (96.9 to 221.0)	1.19 (1.11 to 1.27)	1.01 (0.79 to 1.29)
3650	89	105	973.9 (926.1 to 1026.4)	1154.8 (1098.9 to 1209.2)	180.9 (104.6 to 251.1)	1.19 (1.10 to 1.27)	1.19 (0.89 to 1.59)
4015	56	67	1096.0 (1038.0 to 1157.0)	1293.5 (1222.2 to 1354.5)	197.5 (101.6 to 285.0)	1.18 (1.09 to 1.27)	1.20 (0.88 to 1.73)
4380	38	32	1241.3 (1159.1 to 1312.6)	1426.7 (1344.0 to 1508.5)	185.5 (78.1 to 299.4)	1.15 (1.06 to 1.25)	0.85 (0.54 to 1.40)
4745	10	13	1325.6 (1231.4 to 1421.3)	1544.1 (1435.5 to 1664.5)	218.5 (77.4 to 377.6)	1.16 (1.06 to 1.29)	1.31 (0.54 to 3.35)
5110	5	7	1471.5 (1322.6 to 1632.2)	1714.3 (1549.6 to 1891.4)	242.8 (-4.7 to 475.4)	1.16 (1.00 to 1.34)	***

*** IRR/RR was not estimated when there were fewer than 20 events in the risk period or fewer than 5 events in one group. Confidence intervals obtained through percentile bootstrap.

Supplementary Table 7. Estimated risk of death by cause-specific (defined by ICD-10 blocks) comparing diagnosed Tuberculosis cases to non-exposed controls.

Time - Days	Number of events		Risk per 100,000		Risk difference per 100,000 - 95% CI	Risk ratio - 95% CI	Incidence rate ratio - 95% CI
	Unexposed	Exposed	Unexposed	Exposed			
**Cause: Cancer Respiratory**							
30	1	96	0.5 (0.0 to 1.6)	51.8 (41.8 to 63.4)	51.3 (41.3 to 63.4)	***	***
90	2	141	1.6 (0.0 to 3.8)	129.3 (113.8 to 145.8)	127.6 (111.8 to 143.6)	***	***
180	4	92	3.9 (1.1 to 6.7)	181.3 (163.9 to 201.3)	177.4 (159.0 to 196.9)	46.35 (26.96 to 156.92)	***
365	5	140	6.9 (3.4 to 10.6)	265.0 (244.6 to 291.7)	258.1 (237.5 to 285.2)	38.36 (25.02 to 80.07)	29.71 (15.74 to 145.83)
730	13	131	15.6 (9.5 to 21.6)	351.2 (326.6 to 380.4)	335.7 (310.1 to 363.9)	22.56 (16.05 to 37.23)	10.84 (6.47 to 23.01)
1095	14	61	26.6 (18.6 to 35.0)	397.9 (371.7 to 429.1)	371.3 (342.8 to 406.1)	14.97 (11.19 to 21.87)	4.75 (2.73 to 9.13)
1460	13	39	37.9 (27.5 to 48.9)	432.8 (404.8 to 468.7)	394.9 (364.2 to 434.0)	11.41 (8.95 to 16.14)	3.30 (1.90 to 7.54)
1825	13	37	51.6 (39.0 to 64.0)	471.6 (441.1 to 507.9)	420.0 (384.6 to 459.4)	9.14 (7.26 to 12.24)	3.16 (1.73 to 6.97)
2190	11	36	65.3 (51.3 to 79.8)	516.4 (483.6 to 553.9)	451.1 (412.3 to 491.3)	7.91 (6.34 to 10.25)	3.67 (1.96 to 8.13)
2555	9	18	79.0 (62.2 to 97.5)	543.7 (507.0 to 586.9)	464.7 (419.8 to 511.5)	6.88 (5.42 to 8.68)	2.26 (0.98 to 5.94)
2920	8	14	95.1 (74.7 to 117.6)	570.5 (532.1 to 617.5)	475.4 (427.0 to 524.0)	6.00 (4.79 to 7.60)	1.99 (0.88 to 5.28)
3285	5	14	108.2 (85.5 to 133.3)	606.3 (559.8 to 656.2)	498.1 (444.5 to 550.9)	5.60 (4.47 to 7.22)	***
3650	6	12	128.7 (101.5 to 158.3)	649.2 (599.5 to 701.4)	520.6 (461.5 to 584.7)	5.05 (3.99 to 6.53)	***
4015	3	5	143.7 (112.6 to 177.8)	676.7 (622.3 to 738.6)	533.0 (465.1 to 603.0)	4.71 (3.72 to 6.06)	***
4380	3	6	178.2 (131.7 to 229.7)	739.2 (672.1 to 825.2)	561.0 (474.1 to 657.4)	4.15 (3.18 to 5.78)	***
4745	0	2	178.2 (131.7 to 229.7)	780.6 (694.5 to 885.7)	602.4 (489.8 to 712.6)	4.38 (3.27 to 6.03)	***
5110	0	0	178.2 (131.7 to 229.7)	780.6 (694.5 to 885.7)	602.4 (489.8 to 712.6)	4.38 (3.27 to 6.03)	***
**Cause: Cancer Digestive Organs**							
30	4	33	2.2 (0.5 to 4.3)	17.8 (11.9 to 23.8)	15.7 (9.2 to 21.6)	***	***
90	7	43	6.0 (2.7 to 9.3)	41.4 (31.5 to 51.0)	35.4 (24.5 to 44.8)	6.88 (4.05 to 15.96)	6.37 (3.27 to 22.56)
180	8	46	10.6 (6.6 to 15.1)	67.5 (55.4 to 79.1)	56.9 (43.7 to 68.9)	6.38 (4.18 to 10.70)	6.03 (3.21 to 16.16)
365	19	70	22.0 (15.5 to 29.0)	109.3 (93.2 to 123.7)	87.3 (68.9 to 102.6)	4.97 (3.60 to 6.96)	3.91 (2.45 to 6.50)
730	38	85	47.3 (37.5 to 58.5)	165.7 (147.9 to 184.1)	118.4 (96.7 to 139.7)	3.50 (2.71 to 4.59)	2.41 (1.73 to 3.70)
1095	28	62	69.0 (56.6 to 81.8)	214.1 (189.7 to 236.6)	145.1 (118.9 to 171.3)	3.10 (2.50 to 4.00)	2.41 (1.58 to 3.93)
1460	44	47	108.8 (91.9 to 127.0)	256.3 (229.0 to 283.7)	147.5 (112.1 to 180.2)	2.36 (1.92 to 2.89)	1.17 (0.78 to 1.78)
1825	23	59	133.2 (113.8 to 155.9)	317.9 (287.2 to 348.8)	184.7 (144.9 to 222.5)	2.39 (1.97 to 2.85)	2.85 (1.77 to 4.80)
2190	19	38	156.8 (133.9 to 182.8)	365.1 (329.2 to 400.9)	208.3 (161.8 to 252.5)	2.33 (1.94 to 2.83)	2.24 (1.33 to 4.26)
2555	15	26	180.0 (153.3 to 204.3)	404.8 (365.2 to 443.1)	224.8 (176.5 to 272.7)	2.25 (1.90 to 2.71)	1.96 (1.06 to 3.93)

2920	15	26	209.3 (178.4 to 238.8)	455.1 (411.4 to 495.8)	245.8 (191.2 to 299.0)	2.17 (1.85 to 2.63)	1.98 (1.14 to 4.08)
3285	7	21	227.3 (192.7 to 260.6)	508.2 (457.9 to 556.1)	280.9 (218.8 to 337.3)	2.24 (1.90 to 2.67)	3.45 (1.54 to 11.47)
3650	8	14	255.8 (217.0 to 292.6)	557.5 (504.2 to 616.5)	301.7 (237.2 to 368.1)	2.18 (1.83 to 2.63)	2.02 (0.91 to 6.80)
4015	7	8	294.0 (249.6 to 342.6)	599.1 (535.6 to 658.0)	305.1 (234.5 to 383.5)	2.04 (1.69 to 2.48)	***
4380	5	8	340.7 (280.4 to 404.2)	667.0 (587.7 to 738.7)	326.3 (231.0 to 417.5)	1.96 (1.61 to 2.48)	***
4745	1	1	357.1 (286.2 to 426.0)	704.1 (600.0 to 810.6)	347.0 (225.8 to 470.9)	1.97 (1.55 to 2.57)	***
5110	0	0	357.1 (286.2 to 426.0)	704.1 (600.0 to 810.6)	347.0 (225.8 to 470.9)	1.97 (1.55 to 2.57)	***
**Cause: Ischemic heart diseases**							
30	3	39	1.6 (0.0 to 3.8)	21.0 (14.6 to 28.6)	19.4 (13.0 to 27.3)	***	***
90	12	34	8.2 (4.4 to 12.6)	39.7 (31.5 to 48.7)	31.5 (21.7 to 41.8)	4.83 (2.91 to 9.41)	2.94 (1.59 to 5.70)
180	18	44	18.4 (12.3 to 25.1)	64.7 (53.1 to 77.4)	46.2 (33.0 to 59.4)	3.51 (2.43 to 5.27)	2.57 (1.51 to 4.79)
365	33	41	38.2 (29.0 to 48.1)	89.2 (74.7 to 103.7)	50.9 (34.0 to 68.2)	2.33 (1.77 to 3.20)	1.32 (0.81 to 2.04)
730	62	112	79.4 (65.9 to 92.4)	163.2 (144.6 to 184.1)	83.8 (59.8 to 106.8)	2.06 (1.66 to 2.52)	1.94 (1.44 to 2.70)
1095	68	86	131.7 (114.1 to 149.0)	229.2 (204.4 to 256.3)	97.6 (68.0 to 127.5)	1.74 (1.48 to 2.08)	1.38 (1.04 to 1.94)
1460	56	75	182.1 (159.2 to 203.4)	296.8 (267.6 to 327.4)	114.6 (78.6 to 151.9)	1.63 (1.40 to 1.91)	1.47 (1.05 to 2.14)
1825	43	64	227.4 (200.9 to 254.3)	364.4 (332.0 to 397.6)	137.0 (97.3 to 181.6)	1.60 (1.39 to 1.87)	1.65 (1.07 to 2.44)
2190	39	48	276.2 (247.1 to 305.6)	424.7 (386.8 to 460.2)	148.5 (99.2 to 195.8)	1.54 (1.34 to 1.77)	1.38 (0.94 to 2.08)
2555	44	38	344.4 (307.2 to 379.7)	483.6 (443.0 to 525.5)	139.2 (83.3 to 194.1)	1.40 (1.23 to 1.61)	0.98 (0.64 to 1.54)
2920	19	38	382.6 (340.9 to 420.8)	558.5 (512.2 to 605.8)	175.9 (114.7 to 237.4)	1.46 (1.28 to 1.68)	2.28 (1.44 to 4.26)
3285	16	23	424.3 (377.8 to 465.9)	617.6 (563.9 to 672.7)	193.3 (123.2 to 267.8)	1.46 (1.27 to 1.67)	1.65 (0.81 to 3.35)
3650	15	16	477.3 (426.2 to 524.1)	673.4 (616.5 to 731.3)	196.1 (116.9 to 273.6)	1.41 (1.23 to 1.63)	1.23 (0.57 to 2.81)
4015	6	11	504.2 (448.5 to 557.1)	733.8 (666.1 to 804.6)	229.6 (143.6 to 311.0)	1.46 (1.26 to 1.67)	***
4380	8	11	583.1 (505.9 to 666.4)	846.8 (755.6 to 941.4)	263.7 (134.9 to 395.2)	1.45 (1.22 to 1.75)	***
4745	4	2	675.7 (563.6 to 802.3)	881.8 (777.5 to 994.5)	206.2 (43.0 to 365.5)	1.31 (1.06 to 1.62)	***
5110	0	1	675.7 (563.6 to 802.3)	992.2 (800.4 to 1256.6)	316.5 (68.3 to 618.1)	1.47 (1.09 to 1.98)	***
**Cause: Cerebrovascular diseases**							
30	5	42	2.7 (0.5 to 5.4)	22.7 (16.2 to 29.7)	20.0 (12.9 to 27.5)	8.39 (3.67 to 39.52)	8.54 (3.73 to 40.20)
90	11	36	8.7 (4.9 to 13.1)	42.4 (33.2 to 52.0)	33.7 (23.9 to 44.5)	4.85 (2.93 to 9.77)	3.39 (1.93 to 8.08)
180	18	30	18.9 (13.3 to 25.1)	59.4 (48.4 to 70.7)	40.5 (27.5 to 53.0)	3.14 (2.21 to 4.58)	1.75 (1.05 to 3.32)
365	42	51	44.0 (35.6 to 54.0)	89.6 (75.3 to 102.4)	45.6 (29.3 to 62.5)	2.04 (1.57 to 2.71)	1.29 (0.86 to 1.96)
730	51	67	78.0 (64.4 to 91.7)	134.3 (117.2 to 152.3)	56.3 (35.2 to 78.1)	1.72 (1.39 to 2.17)	1.41 (1.00 to 2.12)
1095	56	65	120.7 (103.0 to 138.4)	184.4 (163.1 to 206.2)	63.7 (36.0 to 93.9)	1.53 (1.28 to 1.89)	1.26 (0.89 to 1.79)
1460	57	54	172.0 (148.8 to 195.1)	232.5 (207.9 to 256.3)	60.4 (29.3 to 94.0)	1.35 (1.15 to 1.61)	1.04 (0.68 to 1.45)
1825	39	37	212.6 (185.6 to 238.2)	271.0 (243.4 to 301.1)	58.4 (21.4 to 95.1)	1.27 (1.09 to 1.50)	1.05 (0.71 to 1.57)
2190	41	40	264.7 (233.5 to 294.8)	321.5 (290.3 to 358.3)	56.9 (15.9 to 101.8)	1.21 (1.06 to 1.41)	1.09 (0.73 to 1.70)

2555	25	34	304.5 (267.3 to 339.5)	374.3 (338.3 to 413.5)	69.8 (21.8 to 119.2)	1.23 (1.07 to 1.43)	1.54 (0.95 to 2.67)
2920	25	22	353.1 (314.1 to 393.9)	417.2 (378.6 to 459.2)	64.1 (10.9 to 120.0)	1.18 (1.03 to 1.37)	1.00 (0.55 to 1.81)
3285	21	14	408.5 (362.6 to 454.9)	453.1 (414.2 to 502.6)	44.6 (-18.0 to 108.9)	1.11 (0.96 to 1.28)	0.77 (0.37 to 1.41)
3650	20	13	477.1 (425.1 to 530.4)	500.5 (450.8 to 551.5)	23.4 (-42.7 to 95.7)	1.05 (0.92 to 1.22)	0.75 (0.39 to 1.52)
4015	9	10	524.0 (462.3 to 583.4)	552.2 (492.4 to 609.0)	28.2 (-51.6 to 114.3)	1.05 (0.91 to 1.24)	***
4380	6	10	586.1 (510.6 to 672.7)	647.6 (564.3 to 734.3)	61.6 (-46.7 to 183.5)	1.11 (0.93 to 1.34)	***
4745	1	1	603.2 (528.0 to 688.9)	690.7 (587.5 to 819.3)	87.5 (-51.4 to 245.1)	1.14 (0.92 to 1.45)	***
5110	0	0	603.2 (528.0 to 688.9)	690.7 (587.5 to 819.3)	87.5 (-51.4 to 245.1)	1.14 (0.92 to 1.45)	***
**Cause: Accidents**							
30	12	20	6.5 (3.2 to 10.3)	10.8 (6.5 to 15.7)	4.3 (-2.2 to 10.2)	1.66 (0.80 to 3.93)	1.70 (0.81 to 4.01)
90	16	18	15.3 (9.9 to 20.8)	20.7 (13.6 to 27.8)	5.4 (-2.7 to 14.9)	1.35 (0.86 to 2.36)	1.17 (0.57 to 2.38)
180	12	16	22.1 (16.0 to 29.2)	29.8 (22.6 to 38.5)	7.7 (-2.1 to 18.8)	1.35 (0.93 to 2.10)	1.40 (0.64 to 3.22)
365	37	53	44.2 (34.9 to 53.6)	61.6 (49.5 to 74.3)	17.3 (2.0 to 32.5)	1.39 (1.04 to 1.89)	1.52 (1.01 to 2.38)
730	59	96	83.5 (70.6 to 96.6)	125.4 (106.3 to 141.9)	41.8 (19.2 to 63.3)	1.50 (1.22 to 1.85)	1.75 (1.27 to 2.38)
1095	52	79	123.3 (105.9 to 140.9)	186.5 (164.5 to 207.7)	63.1 (36.5 to 90.9)	1.51 (1.27 to 1.81)	1.66 (1.14 to 2.55)
1460	39	58	158.2 (139.5 to 179.2)	237.8 (211.2 to 263.1)	79.7 (47.2 to 113.6)	1.50 (1.27 to 1.78)	1.64 (1.10 to 2.54)
1825	51	48	211.1 (188.2 to 238.8)	288.0 (258.3 to 319.4)	76.9 (35.9 to 117.9)	1.36 (1.16 to 1.61)	1.04 (0.69 to 1.53)
2190	21	49	237.6 (210.5 to 268.9)	349.6 (313.3 to 386.8)	112.0 (63.9 to 156.2)	1.47 (1.25 to 1.73)	2.61 (1.59 to 4.56)
2555	33	35	288.9 (256.3 to 326.3)	404.3 (364.7 to 446.4)	115.4 (61.8 to 164.7)	1.40 (1.19 to 1.62)	1.20 (0.71 to 2.01)
2920	21	26	331.0 (293.8 to 371.8)	455.8 (411.4 to 501.7)	124.8 (62.7 to 182.3)	1.38 (1.17 to 1.59)	1.41 (0.78 to 2.54)
3285	14	15	367.9 (327.4 to 414.3)	492.3 (443.9 to 541.9)	124.4 (58.5 to 190.6)	1.34 (1.15 to 1.56)	1.23 (0.57 to 2.77)
3650	7	10	392.7 (348.4 to 439.5)	527.0 (472.6 to 578.0)	134.4 (63.4 to 203.5)	1.34 (1.14 to 1.56)	***
4015	5	3	421.6 (369.5 to 477.6)	542.8 (489.2 to 599.9)	121.3 (43.7 to 201.1)	1.29 (1.09 to 1.52)	***
4380	2	4	438.0 (381.4 to 501.5)	574.8 (513.6 to 645.6)	136.8 (56.6 to 224.8)	1.31 (1.11 to 1.57)	***
4745	2	3	473.1 (401.3 to 547.8)	629.4 (550.5 to 726.1)	156.3 (44.1 to 276.8)	1.33 (1.08 to 1.65)	***
5110	0	1	473.1 (401.3 to 547.8)	763.5 (563.7 to 1043.4)	290.3 (83.1 to 583.1)	1.61 (1.17 to 2.38)	***
**Cause: Assault**							
30	13	23	7.0 (3.2 to 10.8)	12.4 (8.1 to 17.6)	5.4 (-0.6 to 12.4)	1.77 (0.94 to 4.09)	1.80 (0.96 to 4.16)
90	24	39	20.2 (13.9 to 26.8)	33.8 (25.6 to 42.0)	13.6 (3.3 to 23.7)	1.67 (1.13 to 2.54)	1.68 (1.05 to 2.89)
180	26	109	35.0 (26.3 to 43.4)	95.6 (81.6 to 108.7)	60.6 (45.2 to 76.5)	2.73 (2.08 to 3.74)	4.40 (2.93 to 7.09)
365	66	190	74.4 (62.3 to 86.5)	209.4 (188.2 to 231.2)	135.0 (109.1 to 159.8)	2.81 (2.30 to 3.45)	3.05 (2.30 to 4.06)
730	96	324	138.1 (120.8 to 155.5)	424.7 (394.6 to 457.1)	286.6 (248.4 to 323.0)	3.07 (2.64 to 3.59)	3.63 (2.87 to 4.69)
1095	91	211	208.1 (186.4 to 230.4)	586.4 (551.3 to 625.9)	378.4 (330.8 to 420.5)	2.82 (2.46 to 3.20)	2.53 (1.95 to 3.23)
1460	81	167	280.4 (253.6 to 308.1)	736.7 (696.6 to 781.8)	456.3 (408.5 to 507.3)	2.63 (2.35 to 2.94)	2.27 (1.78 to 2.99)
1825	48	144	331.0 (300.9 to 364.1)	887.9 (837.8 to 937.5)	557.0 (494.0 to 619.4)	2.68 (2.39 to 3.01)	3.33 (2.45 to 4.79)

2190	37	99	377.4 (344.7 to 412.8)	1012.6 (961.4 to 1062.8)	635.2 (569.6 to 697.3)	2.68 (2.41 to 2.97)	3.00 (2.10 to 4.55)
2555	31	72	425.1 (388.4 to 463.5)	1123.7 (1065.7 to 1185.0)	698.6 (627.8 to 766.8)	2.64 (2.40 to 2.95)	2.63 (1.76 to 4.27)
2920	30	56	484.1 (441.8 to 530.7)	1230.9 (1163.2 to 1297.8)	746.9 (663.8 to 818.5)	2.54 (2.27 to 2.83)	2.13 (1.36 to 3.43)
3285	19	46	533.3 (482.5 to 588.4)	1347.4 (1273.4 to 1422.6)	814.2 (715.1 to 897.6)	2.53 (2.24 to 2.82)	2.78 (1.70 to 4.77)
3650	16	21	589.8 (536.9 to 655.2)	1417.5 (1333.0 to 1496.7)	827.7 (715.7 to 921.0)	2.40 (2.10 to 2.67)	1.52 (0.73 to 3.21)
4015	8	14	634.2 (569.5 to 701.6)	1494.1 (1403.9 to 1583.0)	859.9 (744.4 to 969.8)	2.36 (2.07 to 2.66)	2.04 (0.91 to 6.59)
4380	4	4	671.3 (598.1 to 747.2)	1531.9 (1434.0 to 1628.0)	860.6 (737.5 to 983.5)	2.28 (2.02 to 2.60)	***
4745	2	2	709.8 (623.6 to 808.4)	1614.3 (1470.6 to 1791.2)	904.4 (732.9 to 1085.2)	2.27 (1.95 to 2.66)	***
5110	0	0	709.8 (623.6 to 808.4)	1614.3 (1470.6 to 1791.2)	904.4 (732.9 to 1085.2)	2.27 (1.95 to 2.66)	***

*** IRR/RR was not estimated when there were fewer than 20 events in the risk period or fewer than 5 events in one group. Confidence intervals obtained through percentile bootstrap.

Supplementary Table 8. Estimated risk of death by cause-specific (defined by ICD-10 blocks) comparing treated Tuberculosis cases to non-exposed controls.

	Number of events		Risk per 100,000				
**Time - Days**	**Unexposed**	**Exposed**	**Unexposed**	**Exposed**	**Risk difference per 100,000 - 95% CI**	**Risk ratio - 95% CI**	**Incidence rate ratio - 95% CI**
				**Cause: Cancer Respiratory**			
30	1	12	0.9 (0.0 to 3.6)	10.8 (4.5 to 17.1)	9.9 (3.6 to 16.2)	***	***
90	0	27	0.9 (0.0 to 3.6)	35.5 (23.8 to 47.4)	34.6 (22.8 to 46.5)	***	***
180	3	30	3.7 (0.9 to 7.9)	63.8 (49.5 to 79.6)	60.1 (45.5 to 76.5)	***	***
365	3	44	6.8 (1.9 to 12.4)	107.7 (87.8 to 127.9)	100.9 (80.5 to 120.6)	15.90 (8.21 to 55.80)	***
730	8	57	15.8 (8.5 to 24.1)	171.5 (146.8 to 197.4)	155.8 (129.4 to 183.7)	10.89 (6.98 to 20.99)	7.25 (3.74 to 20.53)
1095	15	30	35.4 (22.9 to 49.0)	211.0 (181.3 to 239.5)	175.6 (144.6 to 207.3)	5.96 (4.21 to 9.42)	2.05 (1.16 to 4.06)
1460	9	28	50.0 (34.2 to 63.9)	256.8 (222.3 to 293.6)	206.8 (170.3 to 246.3)	5.14 (3.85 to 7.71)	3.22 (1.67 to 8.59)
1825	8	23	65.4 (45.0 to 84.3)	300.6 (264.0 to 337.3)	235.1 (193.4 to 278.2)	4.59 (3.48 to 6.61)	3.00 (1.45 to 8.09)
2190	4	18	74.6 (52.5 to 95.0)	341.9 (297.4 to 382.4)	267.2 (222.3 to 314.9)	4.58 (3.48 to 6.61)	***
2555	4	10	86.0 (59.7 to 110.0)	370.4 (325.3 to 415.9)	284.4 (233.3 to 335.8)	4.31 (3.20 to 6.08)	***
2920	6	11	107.4 (76.2 to 140.4)	410.8 (358.7 to 462.7)	303.3 (244.5 to 363.8)	3.82 (2.83 to 5.54)	***
3285	6	9	136.6 (97.6 to 173.7)	453.1 (400.2 to 510.2)	316.5 (254.9 to 393.4)	3.32 (2.49 to 4.78)	***
3650	3	8	158.3 (114.1 to 204.4)	508.1 (443.1 to 585.1)	349.8 (266.8 to 439.7)	3.21 (2.40 to 4.57)	***
4015	1	2	173.5 (120.7 to 231.1)	529.5 (461.2 to 617.7)	356.0 (263.4 to 462.5)	3.05 (2.24 to 4.60)	***

4380	1	3	190.3 (133.5 to 257.3)	595.7 (492.4 to 714.0)	405.3 (283.8 to 543.5)	3.13 (2.13 to 4.66)	***
4745	0	0	190.3 (133.5 to 257.3)	595.7 (492.4 to 714.0)	405.3 (283.8 to 543.5)	3.13 (2.13 to 4.66)	***
5110	0	0	190.3 (133.5 to 257.3)	595.7 (492.4 to 714.0)	405.3 (283.8 to 543.5)	3.13 (2.13 to 4.66)	***
**Cause: Cancer Digestive Organs**							
30	2	7	1.8 (0.0 to 4.5)	6.3 (2.2 to 11.7)	4.5 (-0.9 to 9.9)	***	***
90	2	20	3.6 (0.9 to 8.1)	24.6 (16.4 to 34.6)	21.0 (11.8 to 31.4)	***	***
180	4	12	7.4 (2.7 to 13.0)	35.9 (24.9 to 47.0)	28.5 (16.4 to 41.2)	4.86 (2.45 to 14.96)	***
365	16	29	23.4 (14.6 to 33.1)	64.9 (50.2 to 81.1)	41.4 (23.3 to 59.5)	2.77 (1.73 to 4.80)	1.83 (1.01 to 3.56)
730	17	37	42.6 (29.7 to 56.0)	106.6 (86.2 to 126.9)	63.9 (36.9 to 87.6)	2.50 (1.73 to 3.66)	2.21 (1.30 to 3.97)
1095	20	42	69.0 (52.4 to 87.8)	161.7 (134.8 to 187.5)	92.7 (57.2 to 122.7)	2.34 (1.71 to 3.28)	2.16 (1.32 to 3.94)
1460	26	56	110.7 (88.8 to 135.3)	251.1 (213.4 to 284.4)	140.4 (92.7 to 179.6)	2.27 (1.73 to 2.94)	2.23 (1.41 to 3.58)
1825	12	29	133.9 (108.7 to 160.5)	308.3 (264.4 to 344.3)	174.4 (123.4 to 219.2)	2.30 (1.82 to 2.95)	2.52 (1.37 to 5.39)
2190	7	16	150.0 (121.1 to 179.7)	345.1 (297.7 to 383.5)	195.1 (137.0 to 240.6)	2.30 (1.82 to 2.94)	2.40 (1.05 to 6.49)
2555	9	20	176.6 (143.1 to 209.6)	402.5 (346.9 to 447.7)	225.9 (157.6 to 284.5)	2.28 (1.79 to 2.99)	2.36 (1.06 to 6.24)
2920	6	14	199.0 (161.6 to 236.4)	453.0 (391.8 to 508.0)	253.9 (176.6 to 322.6)	2.28 (1.77 to 2.89)	2.49 (0.92 to 10.20)
3285	4	10	219.2 (176.1 to 262.7)	503.3 (434.4 to 569.4)	284.1 (196.0 to 362.7)	2.30 (1.75 to 2.92)	***
3650	5	7	253.3 (203.7 to 303.9)	550.6 (474.7 to 621.2)	297.3 (190.2 to 385.4)	2.17 (1.65 to 2.78)	***
4015	5	4	306.9 (238.6 to 384.4)	598.7 (508.4 to 678.7)	291.8 (174.0 to 395.9)	1.95 (1.46 to 2.54)	***
4380	1	0	332.8 (254.4 to 424.0)	598.7 (508.4 to 678.7)	265.9 (122.5 to 382.4)	1.80 (1.31 to 2.43)	***
4745	0	1	332.8 (254.4 to 424.0)	660.4 (521.9 to 831.5)	327.5 (154.6 to 541.9)	1.98 (1.40 to 2.89)	***
5110	0	0	332.8 (254.4 to 424.0)	660.4 (521.9 to 831.5)	327.5 (154.6 to 541.9)	1.98 (1.40 to 2.89)	***
**Cause: Ischaemic heart diseases**							
30	3	3	2.7 (0.0 to 6.3)	2.7 (0.0 to 6.3)	0.0 (-4.5 to 4.5)	***	***
90	8	11	10.0 (4.5 to 16.4)	12.8 (6.4 to 20.1)	2.8 (-6.3 to 11.5)	1.28 (0.52 to 2.80)	***
180	13	16	22.3 (14.8 to 31.6)	28.0 (18.6 to 38.4)	5.7 (-7.3 to 17.8)	1.26 (0.74 to 2.12)	1.24 (0.60 to 2.86)
365	21	39	43.3 (31.3 to 56.9)	67.0 (51.3 to 84.4)	23.7 (3.1 to 44.4)	1.55 (1.06 to 2.28)	1.88 (1.12 to 3.39)
730	46	69	95.2 (77.1 to 115.8)	145.0 (121.2 to 171.6)	49.8 (17.0 to 80.1)	1.52 (1.16 to 1.96)	1.53 (1.02 to 2.19)
1095	32	41	138.4 (116.6 to 162.6)	199.5 (169.5 to 231.3)	61.1 (24.2 to 99.4)	1.44 (1.15 to 1.79)	1.32 (0.86 to 2.10)
1460	34	49	191.7 (165.1 to 221.6)	278.2 (240.5 to 315.9)	86.5 (39.7 to 130.2)	1.45 (1.18 to 1.74)	1.49 (1.00 to 2.32)
1825	26	30	242.1 (207.0 to 282.2)	335.8 (292.6 to 376.6)	93.6 (35.4 to 145.7)	1.39 (1.13 to 1.66)	1.20 (0.71 to 2.24)
2190	25	30	301.0 (258.9 to 346.7)	405.1 (354.9 to 452.8)	104.1 (36.8 to 163.9)	1.35 (1.11 to 1.62)	1.26 (0.75 to 2.23)
2555	15	26	342.3 (293.3 to 396.4)	479.0 (421.6 to 538.7)	136.6 (61.3 to 209.0)	1.40 (1.16 to 1.66)	1.84 (0.91 to 3.70)
2920	9	18	374.8 (321.8 to 432.0)	545.0 (477.2 to 609.8)	170.2 (82.5 to 250.9)	1.45 (1.21 to 1.73)	2.13 (1.06 to 6.05)
3285	12	18	434.6 (378.7 to 499.6)	633.4 (557.8 to 714.9)	198.8 (94.9 to 301.3)	1.46 (1.20 to 1.76)	1.61 (0.76 to 3.53)
3650	7	8	481.5 (414.1 to 556.5)	691.3 (610.8 to 783.3)	209.8 (105.1 to 317.2)	1.44 (1.20 to 1.74)	1.24 (0.60 to 2.86)

4015	5	10	546.8 (458.9 to 643.3)	816.5 (706.2 to 935.5)	269.7 (126.2 to 404.1)	1.49 (1.21 to 1.85)	1.88 (1.12 to 3.39)
4380	5	4	685.3 (550.5 to 857.8)	908.7 (783.6 to 1057.4)	223.4 (7.1 to 430.8)	1.33 (1.01 to 1.72)	1.53 (1.02 to 2.19)
4745	1	0	738.9 (562.8 to 929.0)	908.7 (783.6 to 1057.4)	169.8 (-62.2 to 393.6)	1.23 (0.93 to 1.66)	1.32 (0.86 to 2.10)
5110	0	0	738.9 (562.8 to 929.0)	908.7 (783.6 to 1057.4)	169.8 (-62.2 to 393.6)	1.23 (0.93 to 1.66)	1.49 (1.00 to 2.32)
**Cause: Cerebrovascular diseases**							
30	5	5	4.5 (0.9 to 8.1)	4.5 (0.9 to 8.6)	-0.0 (-5.4 to 6.3)	***	***
90	8	12	11.8 (5.5 to 19.1)	15.5 (8.6 to 23.2)	3.6 (-6.3 to 13.6)	1.31 (0.65 to 3.16)	1.50 (0.70 to 4.68)
180	15	6	26.0 (16.7 to 36.1)	21.1 (12.8 to 30.3)	-4.9 (-16.9 to 7.2)	0.81 (0.48 to 1.41)	0.40 (0.11 to 1.00)
365	10	25	36.0 (24.5 to 47.6)	46.1 (34.5 to 61.0)	10.1 (-5.7 to 27.8)	1.28 (0.87 to 2.01)	2.52 (1.24 to 5.82)
730	31	42	70.4 (54.3 to 86.7)	93.3 (74.5 to 114.1)	22.9 (0.2 to 50.3)	1.32 (1.00 to 1.83)	1.38 (0.90 to 2.22)
1095	30	47	110.3 (88.9 to 131.0)	155.9 (129.1 to 182.5)	45.6 (16.1 to 79.4)	1.41 (1.13 to 1.86)	1.61 (1.05 to 2.47)
1460	25	31	150.3 (125.7 to 175.8)	205.7 (175.6 to 241.1)	55.4 (17.5 to 94.4)	1.37 (1.10 to 1.69)	1.28 (0.79 to 2.07)
1825	18	22	185.0 (154.4 to 214.0)	248.7 (214.7 to 285.0)	63.7 (18.6 to 111.4)	1.34 (1.09 to 1.68)	1.28 (0.70 to 2.58)
2190	17	36	224.1 (187.6 to 260.0)	332.2 (289.2 to 379.8)	108.1 (55.4 to 165.5)	1.48 (1.23 to 1.82)	2.23 (1.34 to 4.25)
2555	16	13	269.0 (228.8 to 312.3)	369.4 (320.9 to 418.6)	100.4 (35.6 to 161.4)	1.37 (1.12 to 1.68)	0.86 (0.39 to 1.64)
2920	13	9	316.5 (269.8 to 372.5)	402.4 (351.0 to 457.5)	86.0 (8.6 to 152.6)	1.27 (1.03 to 1.54)	0.74 (0.27 to 1.78)
3285	17	6	399.8 (341.5 to 469.2)	431.6 (371.6 to 488.2)	31.7 (-65.5 to 116.0)	1.08 (0.86 to 1.32)	0.38 (0.09 to 0.84)
3650	6	7	444.1 (373.2 to 516.4)	480.1 (407.8 to 547.8)	36.1 (-65.4 to 133.8)	1.08 (0.87 to 1.34)	***
4015	5	7	506.8 (424.6 to 601.5)	567.3 (477.8 to 677.3)	60.5 (-72.5 to 192.5)	1.12 (0.88 to 1.43)	***
4380	2	1	544.7 (446.3 to 659.2)	585.7 (492.8 to 702.8)	40.9 (-106.5 to 196.0)	1.08 (0.83 to 1.41)	***
4745	2	1	688.1 (489.3 to 927.2)	659.8 (509.9 to 861.0)	-28.3 (-330.4 to 260.8)	0.96 (0.63 to 1.49)	***
5110	0	0	688.1 (489.3 to 927.2)	659.8 (509.9 to 861.0)	-28.3 (-330.4 to 260.8)	0.96 (0.63 to 1.49)	***
**Cause: Accidents**							
30	2	5	1.8 (0.0 to 4.5)	4.5 (0.9 to 9.0)	2.7 (-1.8 to 7.2)	***	***
90	8	11	9.1 (4.6 to 14.7)	14.6 (8.2 to 21.9)	5.4 (-2.8 to 13.7)	1.60 (0.75 to 3.51)	***
180	8	21	16.7 (10.1 to 25.9)	34.4 (24.1 to 46.3)	17.7 (4.6 to 30.7)	2.06 (1.22 to 3.86)	2.64 (1.26 to 6.77)
365	21	25	37.7 (26.4 to 49.7)	59.4 (46.0 to 75.4)	21.7 (2.1 to 41.1)	1.58 (1.04 to 2.42)	1.20 (0.67 to 2.29)
730	40	53	82.8 (66.5 to 101.0)	119.2 (99.9 to 141.7)	36.4 (8.5 to 63.0)	1.44 (1.08 to 1.88)	1.35 (0.88 to 2.04)
1095	31	44	124.1 (101.7 to 146.1)	178.0 (152.0 to 210.2)	53.9 (16.4 to 93.4)	1.43 (1.11 to 1.89)	1.46 (0.97 to 2.49)
1460	30	31	172.6 (145.9 to 201.9)	227.8 (195.2 to 264.0)	55.3 (11.5 to 98.6)	1.32 (1.06 to 1.65)	1.07 (0.63 to 1.78)
1825	19	28	209.8 (177.4 to 243.5)	282.5 (247.1 to 323.1)	72.7 (25.5 to 122.7)	1.35 (1.11 to 1.67)	1.54 (0.84 to 3.04)
2190	15	24	244.4 (210.1 to 284.7)	338.2 (298.3 to 387.7)	93.8 (39.3 to 153.8)	1.38 (1.14 to 1.67)	1.68 (0.95 to 3.15)
2555	15	16	286.8 (243.1 to 333.7)	383.8 (337.7 to 434.7)	97.0 (32.5 to 162.1)	1.34 (1.11 to 1.63)	1.13 (0.57 to 2.44)
2920	13	12	334.1 (290.2 to 386.4)	427.4 (375.0 to 487.2)	93.3 (18.8 to 163.3)	1.28 (1.05 to 1.53)	0.99 (0.41 to 2.35)
3285	10	8	383.9 (327.8 to 443.6)	467.5 (412.1 to 535.5)	83.6 (0.2 to 166.2)	1.22 (1.00 to 1.47)	***

3650	3	2	403.8 (340.2 to 464.7)	482.7 (419.8 to 556.1)	78.9 (-12.5 to 168.1)	1.20 (0.97 to 1.45)	***
4015	2	3	432.6 (355.3 to 506.0)	519.0 (447.7 to 603.5)	86.4 (-16.3 to 183.2)	1.20 (0.97 to 1.50)	***
4380	1	2	458.9 (377.3 to 560.0)	578.9 (477.6 to 711.2)	120.0 (-35.4 to 268.5)	1.26 (0.93 to 1.65)	***
4745	0	0	458.9 (377.3 to 560.0)	578.9 (477.6 to 711.2)	120.0 (-35.4 to 268.5)	1.26 (0.93 to 1.65)	***
5110	0	1	458.9 (377.3 to 560.0)	827.2 (502.4 to 1409.4)	368.3 (17.6 to 965.0)	1.80 (1.03 to 3.17)	***
**Cause: Assault**							
30	5	13	4.5 (0.9 to 9.0)	11.7 (5.4 to 18.8)	7.2 (-0.5 to 14.4)	***	***
90	14	27	17.3 (10.0 to 25.5)	36.4 (25.5 to 48.7)	19.2 (6.4 to 34.2)	2.11 (1.28 to 3.77)	1.93 (1.06 to 3.85)
180	17	45	33.3 (22.2 to 44.5)	79.0 (62.3 to 96.5)	45.7 (26.1 to 66.1)	2.38 (1.67 to 3.71)	2.66 (1.65 to 5.79)
365	33	92	66.4 (50.2 to 83.2)	171.2 (148.1 to 199.1)	104.7 (77.2 to 135.1)	2.58 (1.98 to 3.48)	2.81 (1.93 to 4.73)
730	54	144	126.8 (104.6 to 147.7)	332.3 (294.5 to 370.9)	205.5 (164.2 to 249.0)	2.62 (2.20 to 3.25)	2.71 (2.04 to 3.79)
1095	53	101	198.1 (169.3 to 230.6)	466.3 (422.7 to 508.4)	268.2 (214.2 to 316.1)	2.35 (1.98 to 2.82)	1.96 (1.37 to 2.71)
1460	29	93	243.8 (212.5 to 278.3)	615.0 (562.1 to 667.1)	371.1 (307.6 to 427.0)	2.52 (2.13 to 2.94)	3.32 (2.27 to 5.14)
1825	35	71	311.6 (273.9 to 355.9)	752.0 (690.0 to 815.5)	440.4 (366.3 to 513.3)	2.41 (2.08 to 2.82)	2.12 (1.42 to 3.19)
2190	20	48	358.9 (316.7 to 406.3)	863.7 (797.1 to 939.0)	504.9 (430.5 to 589.5)	2.41 (2.09 to 2.81)	2.52 (1.56 to 4.34)
2555	21	37	419.1 (365.5 to 471.2)	968.7 (891.3 to 1044.7)	549.6 (462.0 to 636.3)	2.31 (2.01 to 2.71)	1.87 (1.16 to 3.50)
2920	19	28	488.6 (425.5 to 549.5)	1070.0 (988.1 to 1155.9)	581.5 (474.6 to 693.9)	2.19 (1.89 to 2.56)	1.57 (0.84 to 2.86)
3285	9	21	534.1 (465.9 to 601.6)	1171.5 (1074.7 to 1265.9)	637.4 (516.5 to 764.1)	2.19 (1.88 to 2.55)	2.51 (1.30 to 6.69)
3650	6	7	579.3 (501.1 to 660.9)	1218.7 (1123.9 to 1318.0)	639.4 (507.8 to 770.3)	2.10 (1.79 to 2.46)	***
4015	6	8	650.1 (556.2 to 751.4)	1304.5 (1186.9 to 1424.1)	654.5 (483.0 to 815.3)	2.01 (1.66 to 2.38)	***
4380	0	2	650.1 (556.2 to 751.4)	1370.3 (1224.6 to 1520.8)	720.3 (532.1 to 908.2)	2.11 (1.73 to 2.55)	***
4745	0	0	650.1 (556.2 to 751.4)	1370.3 (1224.6 to 1520.8)	720.3 (532.1 to 908.2)	2.11 (1.73 to 2.55)	***
5110	0	0	650.1 (556.2 to 751.4)	1370.3 (1224.6 to 1520.8)	720.3 (532.1 to 908.2)	2.11 (1.73 to 2.55)	***

*** IRR/RR was not estimated when there were fewer than 20 events in the risk period or fewer than 5 events in one group. Confidence intervals obtained through percentile bootstrap.

Supplementary Table 9. Estimated risk of death by cause-specific (defined by ICD-10 blocks) comparing household Tuberculosis contacts to non-exposed controls.

	Number of events		Risk per 100,000				
**Time - Days**	**Unexposed**	**Exposed**	**Unexposed**	**Exposed**	**Risk difference per 100,000 - 95% CI**	**Risk ratio - 95% CI**	**Incidence rate ratio - 95% CI**
**Cause: Cancer Respiratory**							
30	0	1	0.0 (0.0 to 0.0)	0.2 (0.0 to 0.7)	0.2 (0.0 to 0.7)	***	***

90	3	6	0.7 (0.0 to 1.6)	1.6 (0.5 to 3.0)	0.9 (-0.6 to 2.4)	***	***
180	8	7	2.6 (1.2 to 4.4)	3.2 (1.8 to 5.1)	0.7 (-1.9 to 3.0)	1.27 (0.53 to 3.16)	***
365	10	14	5.0 (2.9 to 7.2)	6.7 (4.3 to 9.3)	1.7 (-1.7 to 5.2)	1.33 (0.74 to 2.53)	1.40 (0.57 to 4.00)
730	21	23	10.8 (7.4 to 14.2)	13.0 (9.7 to 16.4)	2.2 (-2.2 to 7.2)	1.21 (0.83 to 1.89)	1.10 (0.63 to 2.19)
1095	18	22	16.5 (12.4 to 20.8)	20.1 (15.9 to 24.6)	3.6 (-2.8 to 10.3)	1.22 (0.86 to 1.76)	1.22 (0.67 to 2.31)
1460	16	16	22.5 (17.6 to 27.7)	26.0 (20.6 to 31.6)	3.5 (-3.6 to 11.2)	1.16 (0.85 to 1.58)	1.00 (0.50 to 2.01)
1825	12	15	27.7 (22.2 to 33.3)	32.4 (26.5 to 39.4)	4.7 (-3.4 to 13.7)	1.17 (0.89 to 1.59)	1.25 (0.59 to 3.01)
2190	9	18	32.4 (26.1 to 38.7)	41.8 (34.6 to 49.6)	9.3 (-0.1 to 19.2)	1.29 (1.00 to 1.70)	2.01 (0.92 to 6.02)
2555	7	9	36.8 (30.0 to 43.6)	47.4 (39.0 to 55.9)	10.6 (-0.3 to 20.6)	1.29 (0.99 to 1.64)	***
2920	13	8	47.3 (38.5 to 55.9)	53.8 (44.5 to 64.1)	6.4 (-6.3 to 19.2)	1.14 (0.88 to 1.47)	0.62 (0.19 to 1.41)
3285	3	7	50.4 (40.5 to 59.9)	61.0 (50.0 to 72.4)	10.6 (-3.0 to 25.5)	1.21 (0.95 to 1.55)	***
3650	3	0	54.5 (44.1 to 64.9)	61.0 (50.0 to 72.4)	6.6 (-7.7 to 22.4)	1.12 (0.88 to 1.47)	***
4015	5	3	64.5 (50.6 to 77.6)	67.0 (53.8 to 80.3)	2.5 (-16.0 to 22.3)	1.04 (0.78 to 1.40)	***
4380	3	2	75.9 (58.0 to 94.1)	73.5 (58.9 to 91.6)	-2.4 (-26.6 to 20.9)	0.97 (0.70 to 1.35)	***
4745	1	0	82.5 (61.3 to 105.2)	73.5 (58.9 to 91.6)	-9.0 (-35.1 to 18.2)	0.89 (0.65 to 1.29)	***
5110	0	0	82.5 (61.3 to 105.2)	73.5 (58.9 to 91.6)	-9.0 (-35.1 to 18.2)	0.89 (0.65 to 1.29)	***
**Cause: Cancer Digestive Organs**							
30	7	8	1.6 (0.7 to 2.9)	1.8 (0.7 to 3.1)	0.2 (-1.6 to 1.8)	***	***
90	5	7	2.7 (1.3 to 4.3)	3.4 (1.9 to 5.2)	0.7 (-1.6 to 2.7)	1.25 (0.59 to 2.85)	***
180	11	17	5.3 (3.2 to 7.6)	7.4 (5.0 to 9.9)	2.1 (-1.0 to 5.6)	1.39 (0.85 to 2.55)	1.55 (0.71 to 3.82)
365	16	27	9.2 (6.4 to 12.3)	14.0 (10.5 to 17.4)	4.8 (0.0 to 9.6)	1.52 (1.00 to 2.39)	1.69 (0.92 to 3.42)
730	47	43	22.3 (17.5 to 26.8)	25.9 (20.7 to 30.5)	3.6 (-2.5 to 9.9)	1.16 (0.90 to 1.53)	0.92 (0.63 to 1.36)
1095	44	40	36.3 (30.6 to 42.4)	38.5 (31.7 to 44.7)	2.2 (-6.2 to 10.5)	1.06 (0.85 to 1.32)	0.91 (0.58 to 1.42)
1460	43	31	52.3 (45.1 to 59.7)	49.8 (41.3 to 57.5)	-2.5 (-13.1 to 8.4)	0.95 (0.77 to 1.18)	0.72 (0.44 to 1.14)
1825	31	28	65.6 (56.0 to 74.3)	61.9 (52.2 to 70.7)	-3.8 (-15.6 to 8.8)	0.94 (0.77 to 1.15)	0.91 (0.54 to 1.59)
2190	19	25	75.5 (65.3 to 85.3)	74.9 (64.8 to 84.8)	-0.6 (-14.4 to 13.8)	0.99 (0.82 to 1.20)	1.32 (0.74 to 2.58)
2555	26	18	92.0 (79.3 to 104.4)	86.5 (75.6 to 98.0)	-5.5 (-22.1 to 10.8)	0.94 (0.78 to 1.13)	0.70 (0.36 to 1.30)
2920	13	16	102.3 (88.3 to 115.0)	99.0 (85.9 to 113.2)	-3.3 (-22.7 to 15.3)	0.97 (0.80 to 1.16)	1.24 (0.56 to 2.64)
3285	13	12	115.8 (101.5 to 130.8)	111.2 (95.5 to 126.8)	-4.7 (-24.6 to 14.3)	0.96 (0.80 to 1.14)	0.93 (0.42 to 1.97)
3650	7	12	125.9 (109.0 to 144.2)	128.0 (111.1 to 147.1)	2.1 (-22.3 to 24.9)	1.02 (0.84 to 1.22)	***
4015	4	8	133.7 (115.2 to 154.1)	145.2 (124.4 to 167.8)	11.5 (-16.6 to 38.9)	1.09 (0.89 to 1.33)	***
4380	5	2	154.1 (127.3 to 180.6)	153.6 (130.5 to 178.6)	-0.5 (-34.3 to 37.4)	1.00 (0.80 to 1.28)	***
4745	0	1	154.1 (127.3 to 180.6)	161.9 (133.6 to 193.2)	7.8 (-28.0 to 49.1)	1.05 (0.83 to 1.38)	***
5110	1	1	175.0 (134.7 to 227.7)	196.5 (139.1 to 289.1)	21.5 (-57.4 to 125.9)	1.12 (0.72 to 1.78)	***
**Cause: Ischaemic heart diseases**							

30	6	7	1.3 (0.4 to 2.5)	1.6 (0.6 to 2.9)	0.2 (-1.2 to 1.7)	***	***
90	15	15	4.8 (2.7 to 6.8)	5.0 (3.0 to 7.3)	0.2 (-2.7 to 3.2)	1.04 (0.55 to 1.91)	1.00 (0.44 to 2.22)
180	27	20	11.1 (8.1 to 14.3)	9.7 (6.7 to 12.9)	-1.4 (-5.9 to 3.1)	0.87 (0.57 to 1.34)	0.74 (0.40 to 1.39)
365	44	52	22.0 (17.7 to 26.7)	22.5 (17.7 to 27.3)	0.5 (-5.8 to 7.2)	1.02 (0.75 to 1.38)	1.18 (0.81 to 1.73)
730	73	87	42.1 (36.0 to 47.9)	46.4 (39.5 to 53.3)	4.3 (-5.2 to 14.0)	1.10 (0.88 to 1.37)	1.19 (0.90 to 1.64)
1095	58	65	60.6 (52.9 to 68.1)	67.1 (57.6 to 76.1)	6.5 (-5.2 to 19.1)	1.11 (0.92 to 1.34)	1.12 (0.80 to 1.57)
1460	66	50	84.9 (74.8 to 93.9)	85.5 (74.8 to 95.9)	0.7 (-14.3 to 15.2)	1.01 (0.84 to 1.20)	0.76 (0.51 to 1.04)
1825	36	44	100.5 (89.5 to 110.1)	104.4 (92.4 to 115.5)	3.8 (-12.8 to 19.6)	1.04 (0.88 to 1.21)	1.23 (0.80 to 1.91)
2190	31	43	116.7 (104.4 to 128.2)	126.5 (113.9 to 140.3)	9.8 (-7.1 to 26.8)	1.08 (0.94 to 1.24)	1.39 (0.91 to 2.27)
2555	37	33	140.3 (125.7 to 154.0)	147.2 (133.3 to 162.9)	6.9 (-11.6 to 27.7)	1.05 (0.92 to 1.21)	0.90 (0.57 to 1.48)
2920	32	36	165.5 (148.9 to 179.7)	175.7 (158.1 to 193.6)	10.1 (-11.7 to 35.4)	1.06 (0.93 to 1.23)	1.13 (0.70 to 1.84)
3285	19	29	184.8 (165.3 to 204.8)	205.7 (184.9 to 225.8)	20.9 (-6.4 to 50.7)	1.11 (0.97 to 1.30)	1.53 (0.88 to 2.94)
3650	20	16	214.1 (192.1 to 236.2)	228.2 (207.4 to 250.6)	14.1 (-16.0 to 47.3)	1.07 (0.93 to 1.25)	0.80 (0.39 to 1.58)
4015	12	7	240.4 (213.2 to 266.2)	244.6 (220.5 to 269.4)	4.1 (-32.2 to 42.9)	1.02 (0.88 to 1.20)	***
4380	3	8	252.0 (222.6 to 282.0)	273.9 (241.0 to 307.7)	22.0 (-19.3 to 69.2)	1.09 (0.93 to 1.31)	***
4745	2	1	264.9 (232.3 to 301.0)	283.9 (250.5 to 326.1)	19.0 (-34.6 to 73.5)	1.07 (0.88 to 1.31)	***
5110	0	1	264.9 (232.3 to 301.0)	311.7 (258.6 to 384.9)	46.7 (-25.6 to 128.0)	1.18 (0.91 to 1.51)	***
				**Cause: Cerebrovascular diseases**			
30	5	10	1.1 (0.2 to 2.2)	2.2 (0.9 to 3.6)	1.1 (-0.4 to 2.7)	***	***
90	7	12	2.7 (1.4 to 4.3)	5.0 (3.0 to 7.2)	2.3 (-0.1 to 4.8)	1.83 (0.96 to 4.11)	***
180	26	23	8.8 (6.3 to 11.9)	10.4 (7.5 to 13.5)	1.5 (-2.4 to 5.4)	1.18 (0.76 to 1.79)	0.88 (0.47 to 1.55)
365	36	38	17.8 (13.5 to 22.3)	19.8 (15.8 to 23.7)	2.0 (-3.6 to 7.4)	1.11 (0.83 to 1.51)	1.06 (0.67 to 1.67)
730	76	73	38.7 (32.8 to 45.1)	39.7 (33.7 to 45.7)	1.0 (-7.7 to 8.9)	1.03 (0.82 to 1.26)	0.96 (0.68 to 1.34)
1095	53	72	55.6 (48.0 to 63.5)	62.6 (54.6 to 70.9)	7.0 (-3.4 to 17.2)	1.13 (0.94 to 1.34)	1.36 (0.98 to 1.98)
1460	44	48	71.9 (63.2 to 80.2)	80.5 (71.3 to 89.7)	8.6 (-4.2 to 21.1)	1.12 (0.95 to 1.32)	1.09 (0.74 to 1.67)
1825	50	49	93.6 (83.9 to 104.9)	101.7 (90.4 to 112.5)	8.1 (-7.4 to 22.4)	1.09 (0.93 to 1.25)	0.98 (0.66 to 1.39)
2190	37	42	112.6 (101.6 to 125.1)	123.4 (111.2 to 135.7)	10.8 (-8.4 to 25.7)	1.10 (0.93 to 1.25)	1.14 (0.77 to 1.78)
2555	27	32	129.3 (116.1 to 143.4)	143.9 (129.8 to 158.4)	14.6 (-4.1 to 31.8)	1.11 (0.97 to 1.26)	1.19 (0.71 to 2.03)
2920	31	29	154.2 (140.7 to 171.7)	166.6 (149.8 to 183.3)	12.4 (-9.7 to 32.9)	1.08 (0.94 to 1.23)	0.94 (0.54 to 1.57)
3285	18	24	172.4 (155.7 to 192.6)	191.0 (171.2 to 210.3)	18.5 (-6.1 to 41.0)	1.11 (0.97 to 1.25)	1.34 (0.72 to 2.57)
3650	20	18	202.1 (181.1 to 224.5)	215.7 (191.2 to 238.0)	13.6 (-17.7 to 40.6)	1.07 (0.92 to 1.22)	0.91 (0.50 to 1.68)
4015	12	8	227.0 (202.1 to 255.5)	231.9 (207.0 to 257.6)	4.9 (-31.3 to 39.9)	1.02 (0.87 to 1.19)	0.67 (0.22 to 1.58)
4380	7	7	250.6 (219.9 to 288.1)	258.4 (228.1 to 294.6)	7.8 (-38.3 to 54.8)	1.03 (0.85 to 1.24)	***
4745	4	2	303.8 (246.1 to 372.6)	279.6 (239.9 to 329.2)	-24.2 (-107.6 to 54.4)	0.92 (0.70 to 1.21)	***
5110	1	0	324.6 (253.8 to 409.1)	279.6 (239.9 to 329.2)	-45.0 (-134.5 to 53.3)	0.86 (0.66 to 1.20)	***

				**Cause: Accidents**				
30	9	6	2.0 (0.7 to 3.6)	1.3 (0.4 to 2.5)	-0.7 (-2.5 to 0.9)	0.67 (0.20 to 1.99)	***
90	20	17	6.6 (4.3 to 9.0)	5.2 (3.2 to 7.3)	-1.4 (-4.6 to 1.4)	0.79 (0.44 to 1.30)	0.85 (0.40 to 1.54)
180	22	26	11.8 (9.0 to 15.0)	11.4 (8.3 to 14.4)	-0.4 (-4.9 to 4.2)	0.97 (0.65 to 1.45)	1.18 (0.66 to 2.00)
365	58	73	26.2 (21.3 to 31.0)	29.4 (24.4 to 34.8)	3.2 (-4.3 to 10.2)	1.12 (0.86 to 1.44)	1.26 (0.91 to 1.81)
730	82	85	48.5 (41.7 to 54.9)	52.7 (45.3 to 59.4)	4.2 (-6.1 to 13.8)	1.09 (0.88 to 1.31)	1.04 (0.77 to 1.43)
1095	86	73	75.9 (67.2 to 85.0)	75.9 (67.4 to 84.4)	-0.1 (-12.7 to 10.6)	1.00 (0.85 to 1.15)	0.85 (0.62 to 1.14)
1460	61	68	98.3 (89.0 to 108.2)	101.0 (90.6 to 111.1)	2.7 (-12.8 to 16.2)	1.03 (0.88 to 1.18)	1.12 (0.79 to 1.59)
1825	48	69	118.8 (108.3 to 130.5)	131.1 (118.4 to 143.5)	12.3 (-5.8 to 28.8)	1.10 (0.95 to 1.26)	1.44 (1.03 to 2.11)
2190	49	65	144.1 (131.9 to 158.0)	164.7 (149.2 to 181.1)	20.6 (-0.7 to 40.3)	1.14 (1.00 to 1.30)	1.33 (0.91 to 1.98)
2555	47	38	173.9 (158.4 to 188.9)	188.7 (171.7 to 206.6)	14.8 (-8.8 to 39.6)	1.08 (0.95 to 1.24)	0.81 (0.52 to 1.25)
2920	47	33	211.5 (193.5 to 229.5)	214.8 (196.3 to 234.9)	3.3 (-25.4 to 33.0)	1.02 (0.89 to 1.17)	0.71 (0.44 to 1.11)
3285	32	26	244.7 (222.8 to 266.2)	242.2 (222.0 to 267.3)	-2.5 (-33.7 to 31.5)	0.99 (0.87 to 1.13)	0.82 (0.48 to 1.38)
3650	21	18	273.5 (248.1 to 298.2)	267.9 (244.3 to 296.0)	-5.6 (-41.3 to 30.9)	0.98 (0.86 to 1.12)	0.86 (0.43 to 1.70)
4015	12	19	301.8 (272.6 to 332.5)	306.5 (276.6 to 338.4)	4.7 (-43.1 to 50.8)	1.02 (0.87 to 1.18)	1.59 (0.71 to 3.44)
4380	10	5	343.2 (303.3 to 382.7)	326.7 (296.1 to 362.6)	-16.5 (-68.5 to 41.1)	0.95 (0.82 to 1.13)	***
4745	1	2	351.1 (309.1 to 396.8)	343.5 (304.8 to 385.4)	-7.6 (-72.2 to 52.1)	0.98 (0.82 to 1.16)	***
5110	1	0	371.7 (317.7 to 432.5)	343.5 (304.8 to 385.4)	-28.2 (-102.4 to 44.9)	0.92 (0.76 to 1.14)	***
				**Cause: Assault**				
30	14	23	3.1 (1.6 to 4.7)	5.2 (2.9 to 7.4)	2.0 (-0.7 to 4.7)	1.64 (0.84 to 3.35)	1.64 (0.84 to 3.36)
90	33	52	10.6 (7.7 to 13.9)	17.0 (12.8 to 21.0)	6.4 (1.1 to 10.9)	1.60 (1.08 to 2.29)	1.58 (1.00 to 2.51)
180	36	61	19.1 (15.4 to 24.1)	31.3 (25.8 to 36.8)	12.2 (5.2 to 18.6)	1.64 (1.23 to 2.14)	1.70 (1.15 to 2.74)
365	78	147	38.4 (32.7 to 44.9)	67.7 (59.9 to 75.6)	29.3 (18.5 to 38.8)	1.76 (1.43 to 2.12)	1.89 (1.41 to 2.56)
730	169	238	85.0 (75.3 to 94.3)	133.2 (120.7 to 144.5)	48.2 (31.3 to 62.4)	1.57 (1.34 to 1.81)	1.41 (1.16 to 1.76)
1095	129	225	126.0 (114.4 to 137.9)	204.4 (188.7 to 218.5)	78.5 (59.1 to 97.5)	1.62 (1.45 to 1.84)	1.75 (1.43 to 2.20)
1460	158	188	184.5 (170.0 to 200.0)	273.3 (255.2 to 290.7)	88.8 (66.4 to 112.2)	1.48 (1.34 to 1.65)	1.19 (0.96 to 1.47)
1825	133	156	242.4 (224.9 to 261.4)	340.7 (318.3 to 360.5)	98.3 (70.7 to 125.4)	1.41 (1.28 to 1.54)	1.18 (0.93 to 1.49)
2190	107	136	298.1 (278.0 to 320.8)	410.7 (385.5 to 436.0)	112.7 (80.6 to 143.5)	1.38 (1.26 to 1.50)	1.28 (0.99 to 1.65)
2555	100	116	361.0 (338.8 to 387.3)	484.7 (455.6 to 512.7)	123.7 (84.6 to 158.6)	1.34 (1.22 to 1.46)	1.16 (0.90 to 1.49)
2920	69	81	415.2 (388.2 to 443.5)	549.5 (518.6 to 582.9)	134.3 (91.0 to 179.5)	1.32 (1.21 to 1.45)	1.18 (0.87 to 1.61)
3285	73	78	490.2 (460.2 to 523.1)	629.4 (593.0 to 661.7)	139.3 (92.5 to 188.5)	1.28 (1.18 to 1.40)	1.07 (0.77 to 1.50)
3650	55	73	568.4 (532.5 to 610.8)	731.4 (687.4 to 774.4)	163.0 (103.3 to 220.5)	1.29 (1.18 to 1.40)	1.33 (0.94 to 1.91)
4015	39	37	651.4 (607.7 to 702.1)	807.6 (753.0 to 859.8)	156.2 (89.3 to 226.8)	1.24 (1.13 to 1.37)	0.96 (0.60 to 1.46)
4380	22	19	730.8 (673.6 to 794.2)	887.0 (827.9 to 954.8)	156.3 (64.8 to 243.4)	1.21 (1.08 to 1.35)	0.87 (0.45 to 1.71)
4745	8	9	798.4 (724.4 to 875.2)	967.4 (888.7 to 1056.2)	169.0 (49.3 to 285.3)	1.21 (1.06 to 1.38)	***

5110	3	5	896.4 (778.2 to 1039.8)	1099.2 (957.5 to 1269.0)	202.9 (-0.6 to 404.2)	1.23 (1.00 to 1.50)	***

*** IRR/RR was not estimated when there were fewer than 20 events in the risk period or fewer than 5 events in one group. Confidence intervals obtained through percentile bootstrap.

Supplementary Table 10. Estimated risk of death of natural causes (excluding external causes, TB and HIV deaths) comparing diagnosed Tuberculosis cases to non-exposed controls by subgroup.

	Number of events		Risk per 100,000				
Time - Days	Unexposed	Exposed	Unexposed	Exposed	Risk difference per 100,000 - 95% CI	Risk ratio - 95% CI	Incidence rate ratio - 95% CI
				Subgroup: Male			
30	37	1026	31.7 (22.3 to 41.1)	876.6 (827.0 to 929.0)	844.9 (797.0 to 894.4)	27.68 (21.21 to 39.12)	28.24 (21.63 to 39.89)
90	86	765	106.7 (88.4 to 127.6)	1541.6 (1474.6 to 1619.5)	1434.9 (1368.0 to 1505.2)	14.45 (12.11 to 17.60)	9.25 (7.43 to 12.08)
180	121	578	215.8 (190.2 to 243.1)	2060.9 (1990.2 to 2146.3)	1845.1 (1766.4 to 1929.8)	9.55 (8.51 to 10.96)	5.03 (4.18 to 6.15)
365	245	817	449.3 (410.0 to 490.8)	2838.3 (2747.3 to 2941.2)	2389.0 (2286.1 to 2495.9)	6.32 (5.72 to 6.94)	3.55 (3.10 to 4.13)
730	417	1207	895.2 (842.2 to 955.3)	4125.8 (4014.5 to 4245.0)	3230.6 (3103.9 to 3363.2)	4.61 (4.28 to 4.93)	3.13 (2.84 to 3.54)
1095	389	899	1379.4 (1304.9 to 1460.5)	5244.0 (5109.1 to 5375.9)	3864.6 (3720.5 to 4012.5)	3.80 (3.56 to 4.04)	2.54 (2.27 to 2.86)
1460	380	747	1937.1 (1843.5 to 2027.3)	6332.9 (6180.2 to 6478.9)	4395.7 (4227.4 to 4583.7)	3.27 (3.11 to 3.46)	2.18 (1.94 to 2.49)
1825	305	647	2463.5 (2348.0 to 2579.0)	7444.7 (7276.0 to 7619.5)	4981.1 (4781.3 to 5196.4)	3.02 (2.88 to 3.18)	2.38 (2.11 to 2.75)
2190	253	545	2989.1 (2860.6 to 3104.4)	8568.3 (8364.9 to 8756.5)	5579.1 (5370.1 to 5807.5)	2.87 (2.74 to 3.00)	2.45 (2.14 to 2.83)
2555	179	414	3447.0 (3303.6 to 3587.6)	9619.8 (9402.8 to 9830.5)	6172.8 (5928.8 to 6419.7)	2.79 (2.67 to 2.92)	2.66 (2.26 to 3.17)
2920	142	290	3908.8 (3746.6 to 4075.2)	10554.2 (10309.4 to 10789.9)	6645.4 (6391.6 to 6948.8)	2.70 (2.58 to 2.83)	2.37 (1.95 to 2.93)
3285	124	266	4441.6 (4244.3 to 4635.8)	11688.8 (11417.4 to 11942.2)	7247.2 (6946.8 to 7562.5)	2.63 (2.52 to 2.77)	2.52 (2.04 to 3.15)
3650	92	156	4982.4 (4757.2 to 5217.3)	12625.1 (12324.6 to 12937.5)	7642.7 (7297.8 to 7995.9)	2.53 (2.41 to 2.66)	2.01 (1.56 to 2.59)
4015	57	117	5489.1 (5229.9 to 5745.5)	13714.3 (13351.2 to 14075.5)	8225.2 (7802.2 to 8672.6)	2.50 (2.38 to 2.63)	2.46 (1.83 to 3.38)
4380	32	66	6072.1 (5755.0 to 6397.7)	14869.4 (14409.0 to 15325.0)	8797.3 (8292.1 to 9290.4)	2.45 (2.31 to 2.59)	2.50 (1.74 to 4.06)
4745	16	17	6924.8 (6389.3 to 7455.6)	15662.3 (15045.5 to 16296.5)	8737.5 (7928.7 to 9550.7)	2.26 (2.09 to 2.47)	1.31 (0.62 to 2.99)
5110	5	0	7955.6 (6895.1 to 9105.7)	15662.3 (15045.5 to 16296.5)	7706.7 (6254.4 to 8989.2)	1.97 (1.69 to 2.28)	***
				Subgroup: Female			
30	17	498	24.9 (13.2 to 36.7)	729.5 (667.2 to 794.8)	704.6 (641.6 to 770.6)	29.26 (19.56 to 52.95)	29.72 (19.86 to 53.81)
90	30	354	69.6 (50.5 to 89.6)	1256.0 (1176.6 to 1334.2)	1186.4 (1101.5 to 1270.5)	18.05 (13.82 to 24.33)	12.18 (8.94 to 18.31)
180	45	264	138.5 (109.8 to 167.7)	1659.9 (1564.2 to 1756.3)	1521.4 (1420.3 to 1617.9)	11.98 (9.95 to 15.08)	6.12 (4.45 to 8.49)
365	92	334	287.1 (243.6 to 327.9)	2196.9 (2084.1 to 2305.1)	1909.7 (1793.2 to 2023.7)	7.65 (6.69 to 9.05)	3.82 (3.11 to 4.94)
730	148	485	548.6 (488.6 to 599.3)	3050.6 (2920.2 to 3184.5)	2502.0 (2368.5 to 2643.9)	5.56 (5.03 to 6.27)	3.49 (2.96 to 4.22)

1095	169	338	888.5 (809.6 to 962.8)	3733.8 (3580.0 to 3881.2)	2845.3 (2680.5 to 3001.7)	4.20 (3.86 to 4.63)	2.15 (1.80 to 2.63)
1460	135	303	1202.3 (1115.3 to 1293.2)	4435.5 (4270.5 to 4584.1)	3233.1 (3060.8 to 3419.7)	3.69 (3.42 to 4.03)	2.43 (2.01 to 3.03)
1825	130	256	1553.5 (1442.9 to 1662.2)	5124.2 (4940.5 to 5301.7)	3570.7 (3374.9 to 3772.2)	3.30 (3.07 to 3.56)	2.15 (1.76 to 2.66)
2190	95	186	1860.2 (1730.8 to 1988.8)	5719.8 (5520.7 to 5928.9)	3859.7 (3656.1 to 4081.1)	3.07 (2.88 to 3.31)	2.15 (1.69 to 2.72)
2555	91	173	2219.8 (2069.5 to 2369.1)	6399.8 (6178.3 to 6627.3)	4180.0 (3932.5 to 4432.4)	2.88 (2.70 to 3.10)	2.09 (1.64 to 2.69)
2920	71	115	2570.4 (2391.1 to 2724.5)	6965.8 (6703.1 to 7214.4)	4395.4 (4120.7 to 4657.6)	2.71 (2.54 to 2.91)	1.80 (1.32 to 2.35)
3285	55	106	2924.5 (2738.3 to 3126.0)	7645.5 (7349.5 to 7920.0)	4721.1 (4399.2 to 5019.7)	2.61 (2.44 to 2.80)	2.14 (1.58 to 2.98)
3650	44	78	3301.6 (3067.9 to 3516.3)	8310.9 (7997.5 to 8632.3)	5009.3 (4670.1 to 5362.7)	2.52 (2.35 to 2.71)	1.98 (1.42 to 2.96)
4015	30	55	3699.0 (3430.8 to 3949.9)	8996.6 (8606.8 to 9336.0)	5297.6 (4898.9 to 5681.2)	2.43 (2.26 to 2.63)	2.06 (1.38 to 3.34)
4380	21	32	4193.0 (3884.3 to 4520.7)	9741.1 (9296.4 to 10181.8)	5548.1 (5034.6 to 6090.6)	2.32 (2.14 to 2.54)	1.73 (1.05 to 3.08)
4745	5	13	4501.3 (4066.4 to 4932.1)	10464.9 (9888.6 to 11067.9)	5963.6 (5287.7 to 6715.9)	2.32 (2.10 to 2.60)	***
5110	2	4	4732.7 (4203.8 to 5284.3)	11068.1 (10260.7 to 12066.8)	6335.4 (5319.8 to 7335.9)	2.34 (2.04 to 2.69)	***
**Subgroup: Age <18**							
30	0	70	0.0 (0.0 to 0.0)	560.0 (432.1 to 680.3)	560.0 (432.1 to 680.3)	***	***
90	0	43	0.0 (0.0 to 0.0)	907.2 (750.4 to 1064.7)	907.2 (750.4 to 1064.7)	***	***
180	1	20	8.2 (0.0 to 24.7)	1071.9 (903.0 to 1253.7)	1063.7 (894.2 to 1241.8)	***	***
365	1	33	16.7 (0.0 to 42.2)	1351.5 (1158.2 to 1558.7)	1334.8 (1137.1 to 1542.8)	***	***
730	2	24	35.5 (8.2 to 73.3)	1570.7 (1353.9 to 1807.6)	1535.2 (1335.7 to 1783.1)	44.27 (21.17 to 194.41)	***
1095	2	16	57.2 (18.2 to 115.4)	1743.3 (1516.3 to 1993.6)	1686.1 (1464.6 to 1944.9)	30.47 (15.58 to 102.46)	***
1460	1	19	70.0 (21.8 to 131.7)	1974.9 (1739.0 to 2236.4)	1904.9 (1650.1 to 2177.2)	28.20 (14.49 to 92.20)	***
1825	1	7	82.9 (30.8 to 149.0)	2070.0 (1817.2 to 2341.0)	1987.1 (1726.8 to 2277.8)	24.97 (13.77 to 65.69)	***
2190	3	7	127.9 (56.0 to 212.7)	2175.3 (1909.7 to 2469.5)	2047.3 (1771.0 to 2347.8)	17.01 (10.19 to 38.86)	***
2555	2	8	164.3 (73.2 to 273.2)	2321.2 (2048.8 to 2637.7)	2156.9 (1862.2 to 2494.1)	14.13 (8.60 to 31.50)	***
2920	2	5	208.9 (100.5 to 350.8)	2442.2 (2134.5 to 2772.4)	2233.2 (1915.1 to 2579.3)	11.69 (7.03 to 25.45)	***
3285	0	3	208.9 (100.5 to 350.8)	2528.6 (2213.3 to 2883.6)	2319.7 (1989.4 to 2668.8)	12.10 (7.22 to 25.89)	***
3650	1	2	244.3 (133.1 to 405.3)	2613.8 (2262.1 to 2970.7)	2369.5 (1991.7 to 2775.6)	10.70 (6.45 to 20.71)	***
4015	1	1	289.8 (160.3 to 473.0)	2662.1 (2312.4 to 3038.9)	2372.3 (1966.3 to 2800.4)	9.19 (5.44 to 16.90)	***
4380	0	1	289.8 (160.3 to 473.0)	2730.2 (2364.6 to 3090.2)	2440.4 (2031.8 to 2860.8)	9.42 (5.54 to 17.22)	***
4745	0	0	289.8 (160.3 to 473.0)	2730.2 (2364.6 to 3090.2)	2440.4 (2031.8 to 2860.8)	9.42 (5.54 to 17.22)	***
5110	0	0	289.8 (160.3 to 473.0)	2730.2 (2364.6 to 3090.2)	2440.4 (2031.8 to 2860.8)	9.42 (5.54 to 17.22)	***
**Subgroup: Age 18-59**							
30	21	907	13.7 (7.8 to 19.6)	590.6 (552.5 to 628.7)	576.9 (537.8 to 614.9)	43.08 (30.35 to 72.97)	43.83 (30.86 to 74.28)
90	39	603	39.6 (29.4 to 50.5)	990.0 (941.4 to 1037.8)	950.4 (901.3 to 997.6)	24.98 (19.44 to 33.83)	15.94 (11.80 to 23.18)
180	57	465	78.7 (65.2 to 93.5)	1308.1 (1252.9 to 1362.6)	1229.4 (1173.2 to 1288.1)	16.62 (13.91 to 20.35)	8.49 (6.56 to 11.47)
365	127	638	170.6 (149.3 to 194.6)	1769.5 (1703.9 to 1832.5)	1599.0 (1534.2 to 1663.9)	10.37 (9.15 to 11.89)	5.28 (4.42 to 6.33)

730	236	1019	360.6 (327.3 to 392.5)	2587.9 (2498.9 to 2662.6)	2227.3 (2131.7 to 2305.5)	7.18 (6.50 to 7.90)	4.60 (4.06 to 5.42)
1095	244	777	587.0 (543.9 to 631.5)	3308.2 (3206.6 to 3403.3)	2721.2 (2608.3 to 2813.8)	5.64 (5.21 to 6.13)	3.44 (2.97 to 3.98)
1460	227	658	833.1 (782.4 to 886.6)	4016.8 (3904.1 to 4120.0)	3183.7 (3063.8 to 3290.4)	4.82 (4.48 to 5.14)	3.16 (2.73 to 3.66)
1825	194	587	1079.1 (1013.3 to 1149.6)	4760.5 (4630.8 to 4887.4)	3681.4 (3538.2 to 3818.3)	4.41 (4.13 to 4.73)	3.34 (2.85 to 3.95)
2190	173	480	1344.5 (1269.9 to 1420.1)	5494.9 (5348.9 to 5632.8)	4150.4 (3995.1 to 4318.4)	4.09 (3.85 to 4.36)	3.09 (2.61 to 3.64)
2555	145	368	1615.7 (1535.2 to 1704.4)	6186.8 (6035.1 to 6338.8)	4571.1 (4404.6 to 4756.1)	3.83 (3.62 to 4.07)	2.85 (2.42 to 3.55)
2920	119	279	1898.7 (1799.5 to 1989.4)	6845.5 (6667.4 to 7018.3)	4946.8 (4766.3 to 5151.8)	3.61 (3.42 to 3.82)	2.66 (2.15 to 3.27)
3285	98	261	2204.6 (2077.8 to 2309.9)	7651.9 (7442.5 to 7852.0)	5447.2 (5219.6 to 5680.7)	3.47 (3.28 to 3.70)	3.04 (2.44 to 3.85)
3650	79	173	2535.1 (2401.4 to 2659.4)	8394.8 (8145.7 to 8634.4)	5859.8 (5606.7 to 6122.6)	3.31 (3.13 to 3.52)	2.53 (1.98 to 3.34)
4015	56	127	2904.7 (2737.2 to 3055.6)	9239.9 (8958.6 to 9508.7)	6335.1 (6036.3 to 6651.9)	3.18 (3.00 to 3.39)	2.64 (1.98 to 3.53)
4380	24	71	3199.2 (2994.0 to 3405.3)	10102.7 (9794.5 to 10487.0)	6903.5 (6524.4 to 7343.0)	3.16 (2.95 to 3.40)	3.48 (2.25 to 6.06)
4745	12	23	3607.9 (3291.9 to 3936.8)	10841.5 (10414.0 to 11321.3)	7233.6 (6690.5 to 7824.6)	3.00 (2.74 to 3.33)	2.26 (1.10 to 5.20)
5110	5	3	4116.7 (3624.7 to 4638.2)	11164.1 (10611.5 to 11796.6)	7047.4 (6165.6 to 7858.2)	2.71 (2.35 to 3.14)	***
				**Subgroup: Age ≥60**			
30	33	547	171.6 (116.8 to 233.9)	2841.4 (2615.8 to 3072.8)	2669.8 (2441.4 to 2901.1)	16.56 (12.08 to 23.91)	17.28 (12.59 to 24.94)
90	77	473	580.0 (469.0 to 678.3)	5344.8 (5033.6 to 5654.4)	4764.8 (4440.4 to 5102.2)	9.22 (7.80 to 11.28)	6.79 (5.47 to 9.06)
180	108	357	1173.7 (1013.5 to 1319.6)	7297.8 (6939.1 to 7676.1)	6124.2 (5733.5 to 6548.0)	6.22 (5.47 to 7.24)	3.79 (3.14 to 4.73)
365	209	480	2395.9 (2196.6 to 2639.2)	10089.6 (9696.7 to 10496.1)	7693.6 (7242.3 to 8169.5)	4.21 (3.80 to 4.68)	2.70 (2.30 to 3.15)
730	327	649	4561.4 (4253.8 to 4915.1)	14364.2 (13833.8 to 14905.5)	9802.8 (9220.2 to 10449.4)	3.15 (2.91 to 3.41)	2.41 (2.11 to 2.72)
1095	312	444	7017.8 (6645.1 to 7445.9)	17851.8 (17295.9 to 18447.8)	10834.0 (10119.1 to 11574.3)	2.54 (2.39 to 2.71)	1.77 (1.54 to 2.02)
1460	287	373	9808.9 (9312.7 to 10376.3)	21411.9 (20773.4 to 22082.7)	11603.0 (10794.9 to 12400.4)	2.18 (2.05 to 2.32)	1.64 (1.40 to 1.91)
1825	240	309	12634.6 (12043.6 to 13247.4)	24971.5 (24266.1 to 25760.1)	12336.9 (11480.8 to 13272.4)	1.98 (1.87 to 2.08)	1.66 (1.42 to 2.00)
2190	172	244	15059.3 (14392.1 to 15745.2)	28307.4 (27500.6 to 29106.2)	13248.1 (12221.8 to 14292.9)	1.88 (1.78 to 1.98)	1.87 (1.53 to 2.26)
2555	123	211	17259.1 (16471.8 to 17977.3)	31920.8 (31011.5 to 32872.9)	14661.7 (13529.2 to 15802.0)	1.85 (1.75 to 1.95)	2.32 (1.90 to 3.03)
2920	92	121	19393.5 (18574.8 to 20265.2)	34627.0 (33751.0 to 35577.4)	15233.5 (14018.0 to 16501.6)	1.79 (1.70 to 1.88)	1.82 (1.40 to 2.33)
3285	81	108	22032.1 (21052.4 to 23023.9)	38080.1 (37002.8 to 39239.1)	16048.0 (14637.8 to 17535.1)	1.73 (1.64 to 1.82)	1.90 (1.41 to 2.57)
3650	56	59	24667.5 (23530.8 to 25750.8)	40729.8 (39483.7 to 41970.3)	16062.3 (14548.9 to 17764.7)	1.65 (1.57 to 1.74)	1.53 (1.06 to 2.17)
4015	30	44	26658.2 (25332.3 to 27859.5)	43544.5 (42113.3 to 44913.6)	16886.3 (15237.8 to 18726.2)	1.63 (1.55 to 1.73)	2.15 (1.37 to 3.27)
4380	29	26	30234.9 (28435.8 to 32064.6)	46618.1 (45012.6 to 48365.8)	16383.2 (13977.5 to 18805.4)	1.54 (1.44 to 1.65)	1.33 (0.79 to 2.18)
4745	9	7	32980.4 (30629.1 to 35613.1)	48373.3 (46463.1 to 50786.9)	15392.9 (12117.7 to 19056.8)	1.47 (1.34 to 1.61)	***
5110	2	1	34553.6 (31460.2 to 37707.8)	49454.3 (46884.3 to 52567.3)	14900.6 (10525.5 to 19344.6)	1.43 (1.28 to 1.60)	***
				**Subgroup: White**			
30	14	433	28.4 (14.2 to 43.6)	876.0 (800.0 to 960.2)	847.7 (768.6 to 935.8)	30.89 (20.04 to 62.00)	31.50 (20.44 to 63.33)
90	30	312	90.3 (61.4 to 116.9)	1518.0 (1412.8 to 1633.6)	1427.7 (1324.8 to 1539.7)	16.81 (12.96 to 25.02)	10.82 (7.90 to 16.96)
180	51	202	199.1 (157.7 to 238.3)	1947.5 (1835.9 to 2077.0)	1748.4 (1632.3 to 1885.0)	9.78 (8.07 to 12.61)	4.17 (3.18 to 5.83)

365	95	337	413.2 (358.1 to 467.0)	2706.3 (2569.3 to 2853.6)	2293.0 (2141.7 to 2465.7)	6.55 (5.73 to 7.73)	3.78 (3.04 to 4.73)
730	150	485	791.6 (705.6 to 871.0)	3925.9 (3766.5 to 4114.9)	3134.3 (2957.1 to 3334.1)	4.96 (4.47 to 5.55)	3.49 (2.92 to 4.19)
1095	156	373	1249.0 (1134.2 to 1359.2)	5020.1 (4818.5 to 5215.8)	3771.1 (3548.6 to 4008.5)	4.02 (3.67 to 4.48)	2.62 (2.22 to 3.18)
1460	147	291	1754.5 (1607.3 to 1887.0)	6013.1 (5765.6 to 6251.1)	4258.5 (4011.7 to 4536.3)	3.43 (3.16 to 3.76)	2.19 (1.80 to 2.72)
1825	125	262	2260.7 (2076.2 to 2434.1)	7067.1 (6786.0 to 7317.1)	4806.4 (4509.2 to 5102.8)	3.13 (2.90 to 3.42)	2.34 (1.94 to 2.85)
2190	84	209	2668.6 (2464.5 to 2866.9)	8083.8 (7776.6 to 8352.9)	5415.2 (5087.3 to 5766.1)	3.03 (2.80 to 3.27)	2.81 (2.23 to 3.65)
2555	72	171	3100.6 (2886.5 to 3320.9)	9106.3 (8771.7 to 9424.6)	6005.7 (5646.5 to 6374.0)	2.94 (2.73 to 3.16)	2.72 (2.08 to 3.67)
2920	52	117	3503.4 (3261.4 to 3732.3)	9997.6 (9665.3 to 10353.2)	6494.1 (6104.9 to 6917.4)	2.85 (2.65 to 3.08)	2.61 (1.91 to 3.78)
3285	46	111	3972.0 (3704.0 to 4254.2)	11122.3 (10699.4 to 11522.9)	7150.3 (6683.4 to 7613.4)	2.80 (2.61 to 3.01)	2.82 (2.01 to 4.16)
3650	28	57	4344.6 (4031.8 to 4645.4)	11914.2 (11461.1 to 12356.1)	7569.6 (7090.3 to 8064.4)	2.74 (2.55 to 2.96)	2.41 (1.58 to 3.90)
4015	29	43	4933.4 (4576.3 to 5274.8)	12766.7 (12237.0 to 13277.7)	7833.4 (7230.2 to 8419.2)	2.59 (2.40 to 2.81)	1.78 (1.17 to 2.82)
4380	9	25	5267.2 (4884.8 to 5702.2)	13719.0 (13105.1 to 14397.2)	8451.8 (7773.6 to 9197.1)	2.60 (2.41 to 2.83)	3.36 (1.74 to 8.99)
4745	6	7	5766.6 (5206.2 to 6376.0)	14363.7 (13583.0 to 15185.3)	8597.0 (7718.2 to 9624.3)	2.49 (2.24 to 2.80)	***
5110	0	0	5766.6 (5206.2 to 6376.0)	14363.7 (13583.0 to 15185.3)	8597.0 (7718.2 to 9624.3)	2.49 (2.24 to 2.80)	***
**Subgroup: Black**							
30	5	176	23.7 (4.8 to 47.5)	833.9 (715.8 to 964.2)	810.2 (687.0 to 947.1)	35.12 (17.89 to 170.74)	35.87 (18.30 to 174.25)
90	16	142	100.9 (62.4 to 143.8)	1518.1 (1351.0 to 1684.2)	1417.2 (1249.6 to 1591.9)	15.04 (10.51 to 25.48)	9.25 (6.00 to 18.17)
180	19	87	195.4 (141.1 to 257.9)	1949.8 (1758.5 to 2125.3)	1754.4 (1560.1 to 1939.3)	9.98 (7.49 to 14.35)	4.84 (3.09 to 8.45)
365	40	146	404.8 (322.0 to 489.7)	2711.9 (2482.2 to 2937.1)	2307.2 (2055.9 to 2556.6)	6.70 (5.34 to 8.54)	3.91 (2.74 to 5.57)
730	77	217	846.5 (730.2 to 976.7)	3958.3 (3657.6 to 4192.6)	3111.8 (2781.1 to 3393.8)	4.68 (3.97 to 5.48)	3.07 (2.34 to 3.99)
1095	84	171	1399.9 (1235.6 to 1558.9)	5084.3 (4739.9 to 5372.9)	3684.4 (3310.1 to 4012.5)	3.63 (3.18 to 4.17)	2.26 (1.76 to 2.93)
1460	60	142	1859.5 (1652.6 to 2073.4)	6164.3 (5769.9 to 6494.3)	4304.8 (3887.8 to 4691.9)	3.31 (2.91 to 3.78)	2.66 (2.01 to 3.57)
1825	55	120	2348.1 (2123.6 to 2584.7)	7224.5 (6824.2 to 7594.0)	4876.5 (4385.8 to 5300.0)	3.08 (2.73 to 3.44)	2.48 (1.84 to 3.38)
2190	49	84	2859.3 (2579.4 to 3167.0)	8095.8 (7640.9 to 8512.7)	5236.4 (4733.4 to 5700.3)	2.83 (2.53 to 3.16)	1.97 (1.42 to 2.80)
2555	38	74	3350.3 (3043.0 to 3677.3)	9041.0 (8551.1 to 9527.6)	5690.7 (5138.9 to 6213.8)	2.70 (2.43 to 2.97)	2.26 (1.56 to 3.26)
2920	26	48	3761.5 (3422.5 to 4113.4)	9795.6 (9265.7 to 10320.7)	6034.1 (5455.8 to 6605.5)	2.60 (2.35 to 2.87)	2.16 (1.33 to 3.51)
3285	24	48	4256.5 (3859.3 to 4658.9)	10796.5 (10189.0 to 11382.7)	6540.0 (5873.8 to 7189.5)	2.54 (2.27 to 2.83)	2.35 (1.50 to 4.03)
3650	17	29	4733.5 (4254.2 to 5219.3)	11614.1 (10978.1 to 12346.7)	6880.6 (6154.7 to 7658.5)	2.45 (2.21 to 2.74)	2.02 (1.18 to 3.84)
4015	10	18	5169.3 (4636.6 to 5685.7)	12332.1 (11642.1 to 13115.5)	7162.7 (6335.6 to 8037.7)	2.39 (2.14 to 2.71)	2.15 (1.05 to 5.09)
4380	10	11	6006.5 (5193.5 to 6821.3)	13161.6 (12272.4 to 14053.7)	7155.0 (6035.1 to 8345.6)	2.19 (1.91 to 2.54)	1.33 (0.48 to 3.40)
4745	2	6	6446.8 (5469.9 to 7482.7)	14022.4 (12918.5 to 15191.7)	7575.6 (6122.8 to 9048.7)	2.18 (1.84 to 2.60)	***
5110	2	2	7728.5 (5750.4 to 9999.7)	15069.2 (13579.0 to 16860.9)	7340.8 (4560.2 to 9931.7)	1.95 (1.46 to 2.62)	***
**Subgroup: Mixed**							
30	35	896	31.0 (20.8 to 41.7)	793.2 (743.3 to 839.8)	762.2 (710.8 to 807.2)	25.56 (19.16 to 38.24)	26.01 (19.50 to 38.92)

90	69	655	93.3 (76.2 to 113.8)	1382.7 (1317.1 to 1449.4)	1289.4 (1223.8 to 1362.6)	14.82 (12.18 to 18.21)	9.82 (7.77 to 12.80)
180	95	544	181.7 (157.3 to 207.1)	1887.5 (1814.9 to 1964.0)	1705.8 (1631.1 to 1791.0)	10.39 (9.10 to 12.11)	5.99 (4.94 to 7.51)
365	202	661	380.2 (344.4 to 414.6)	2535.0 (2448.7 to 2624.4)	2154.8 (2067.1 to 2249.6)	6.67 (6.08 to 7.39)	3.46 (3.01 to 4.06)
730	334	983	745.2 (694.0 to 797.7)	3604.9 (3493.8 to 3714.3)	2859.7 (2749.1 to 2979.9)	4.84 (4.49 to 5.21)	3.15 (2.82 to 3.59)
1095	315	688	1141.6 (1074.9 to 1216.0)	4472.0 (4342.8 to 4606.4)	3330.4 (3192.9 to 3466.6)	3.92 (3.66 to 4.17)	2.37 (2.04 to 2.70)
1460	305	610	1590.6 (1513.5 to 1676.2)	5365.3 (5216.7 to 5508.2)	3774.7 (3612.6 to 3934.1)	3.37 (3.20 to 3.57)	2.19 (1.94 to 2.52)
1825	254	519	2027.4 (1931.5 to 2131.7)	6254.8 (6096.2 to 6418.0)	4227.4 (4057.7 to 4411.6)	3.09 (2.93 to 3.26)	2.25 (1.96 to 2.59)
2190	212	436	2467.0 (2350.9 to 2585.7)	7146.5 (6969.9 to 7319.7)	4679.5 (4489.2 to 4894.6)	2.90 (2.76 to 3.05)	2.28 (1.94 to 2.69)
2555	157	341	2864.7 (2732.6 to 3009.9)	8003.6 (7811.0 to 8204.4)	5138.9 (4916.6 to 5390.2)	2.79 (2.66 to 2.96)	2.43 (2.04 to 3.00)
2920	135	238	3295.2 (3147.0 to 3447.1)	8759.7 (8544.4 to 8983.7)	5464.5 (5199.5 to 5754.7)	2.66 (2.53 to 2.80)	1.99 (1.63 to 2.43)
3285	108	208	3748.2 (3566.0 to 3928.5)	9619.6 (9389.2 to 9880.3)	5871.4 (5588.9 to 6196.3)	2.57 (2.43 to 2.71)	2.19 (1.75 to 2.70)
3650	91	148	4270.1 (4089.7 to 4473.8)	10468.0 (10195.2 to 10764.2)	6197.9 (5884.2 to 6548.7)	2.45 (2.33 to 2.60)	1.86 (1.47 to 2.40)
4015	48	109	4688.6 (4466.7 to 4932.8)	11450.2 (11151.3 to 11810.4)	6761.6 (6395.8 to 7164.3)	2.44 (2.32 to 2.58)	2.60 (1.91 to 3.78)
4380	33	62	5245.8 (4925.9 to 5543.5)	12474.2 (12051.4 to 12906.0)	7228.4 (6737.6 to 7760.1)	2.38 (2.24 to 2.56)	2.17 (1.46 to 3.62)
4745	13	17	5892.4 (5403.9 to 6371.6)	13295.1 (12723.3 to 13942.9)	7402.7 (6620.2 to 8191.2)	2.26 (2.05 to 2.48)	1.52 (0.71 to 3.47)
5110	5	2	6525.6 (5786.7 to 7361.6)	13614.8 (12953.1 to 14447.4)	7089.2 (6125.4 to 8180.5)	2.09 (1.85 to 2.38)	***
**Subgroup: HIV**							
30	4	106	26.3 (6.6 to 52.6)	695.6 (554.6 to 826.8)	669.3 (531.4 to 800.8)	***	***
90	6	77	66.5 (29.8 to 106.3)	1209.1 (1017.4 to 1376.9)	1142.6 (944.8 to 1314.2)	18.17 (11.20 to 42.15)	14.77 (8.12 to 46.23)
180	11	65	142.3 (88.2 to 200.6)	1655.1 (1426.0 to 1864.3)	1512.8 (1280.9 to 1720.3)	11.63 (7.88 to 19.20)	7.18 (4.19 to 14.70)
365	12	73	230.2 (157.4 to 299.7)	2186.2 (1951.3 to 2425.3)	1956.0 (1721.8 to 2198.0)	9.50 (6.94 to 14.28)	7.79 (4.67 to 15.64)
730	40	99	552.1 (428.6 to 681.4)	2993.2 (2718.5 to 3282.1)	2441.1 (2159.4 to 2756.4)	5.42 (4.31 to 7.10)	3.38 (2.42 to 5.04)
1095	29	60	827.7 (675.4 to 996.1)	3563.3 (3268.4 to 3877.6)	2735.6 (2396.1 to 3092.4)	4.31 (3.49 to 5.40)	3.00 (1.98 to 4.66)
1460	24	57	1090.6 (909.2 to 1289.2)	4193.3 (3848.0 to 4531.5)	3102.7 (2721.9 to 3484.5)	3.84 (3.18 to 4.63)	3.62 (2.22 to 5.94)
1825	21	50	1368.1 (1170.2 to 1620.5)	4859.2 (4459.8 to 5263.6)	3491.1 (3037.1 to 3933.3)	3.55 (2.97 to 4.32)	3.80 (2.35 to 6.66)
2190	17	33	1633.1 (1398.4 to 1912.1)	5410.1 (4942.9 to 5882.6)	3777.0 (3256.2 to 4321.2)	3.31 (2.79 to 4.01)	3.22 (1.71 to 5.75)
2555	11	28	1852.3 (1579.5 to 2181.6)	5987.8 (5460.9 to 6529.8)	4135.5 (3530.4 to 4684.2)	3.23 (2.69 to 3.89)	4.45 (2.38 to 9.45)
2920	10	14	2114.1 (1826.0 to 2491.5)	6367.8 (5830.3 to 6952.2)	4253.7 (3661.4 to 4841.1)	3.01 (2.52 to 3.59)	2.56 (1.09 to 6.31)
3285	7	19	2352.7 (2029.2 to 2756.8)	7055.2 (6377.7 to 7644.7)	4702.6 (3972.5 to 5349.4)	3.00 (2.50 to 3.55)	5.18 (2.42 to 14.80)
3650	4	12	2546.1 (2162.5 to 3026.8)	7655.8 (6862.7 to 8339.5)	5109.7 (4262.4 to 5921.5)	3.01 (2.45 to 3.65)	***
4015	4	10	2836.4 (2399.4 to 3415.6)	8442.1 (7586.0 to 9306.5)	5605.6 (4563.7 to 6654.1)	2.98 (2.42 to 3.67)	***
4380	3	1	3237.6 (2610.7 to 4001.7)	8655.0 (7724.9 to 9654.4)	5417.4 (4257.5 to 6546.5)	2.67 (2.09 to 3.38)	***
4745	1	0	3454.5 (2732.3 to 4258.3)	8655.0 (7724.9 to 9654.4)	5200.5 (3907.6 to 6498.0)	2.51 (1.94 to 3.32)	***
5110	0	0	3454.5 (2732.3 to 4258.3)	8655.0 (7724.9 to 9654.4)	5200.5 (3907.6 to 6498.0)	2.51 (1.94 to 3.32)	***
**Subgroup: Diabetes**							

30	8	173	82.1 (30.8 to 153.8)	1776.7 (1519.5 to 2054.5)	1694.6 (1402.7 to 1976.5)	21.64 (11.86 to 57.80)	22.23 (12.19 to 59.37)
90	11	119	197.6 (114.0 to 291.3)	3025.5 (2683.5 to 3367.0)	2827.9 (2499.0 to 3181.0)	15.31 (10.31 to 26.50)	11.49 (6.89 to 25.58)
180	18	91	394.0 (275.9 to 520.7)	4018.6 (3610.4 to 4419.1)	3624.6 (3196.0 to 4073.0)	10.20 (7.61 to 14.89)	5.48 (3.38 to 10.03)
365	41	145	873.1 (678.5 to 1096.4)	5709.8 (5214.1 to 6175.4)	4836.7 (4298.9 to 5328.1)	6.54 (5.16 to 8.52)	3.90 (2.76 to 5.56)
730	63	231	1718.9 (1408.0 to 2020.9)	8787.0 (8205.3 to 9400.4)	7068.2 (6431.8 to 7765.8)	5.11 (4.28 to 6.33)	4.14 (3.19 to 5.62)
1095	51	147	2532.5 (2145.4 to 2928.7)	11141.5 (10452.5 to 11883.8)	8609.1 (7846.9 to 9421.1)	4.40 (3.73 to 5.26)	3.33 (2.37 to 4.59)
1460	58	136	3658.8 (3191.1 to 4115.4)	13752.9 (12956.3 to 14670.8)	10094.1 (9125.2 to 11102.6)	3.76 (3.30 to 4.41)	2.77 (2.06 to 3.76)
1825	49	138	4809.8 (4241.1 to 5394.9)	16921.3 (15965.9 to 17863.3)	12111.5 (10950.2 to 13215.9)	3.52 (3.09 to 4.03)	3.42 (2.48 to 4.95)
2190	36	109	5838.3 (5158.1 to 6504.6)	20010.2 (18935.9 to 21084.9)	14172.0 (12883.7 to 15446.9)	3.43 (3.05 to 3.92)	3.79 (2.60 to 5.59)
2555	31	83	6971.4 (6134.1 to 7771.6)	23068.4 (21906.3 to 24339.5)	16097.0 (14607.5 to 17674.4)	3.31 (2.94 to 3.79)	3.47 (2.42 to 5.68)
2920	21	59	8012.3 (7084.9 to 8858.1)	25964.2 (24635.1 to 27418.6)	17951.9 (16249.1 to 19551.0)	3.24 (2.89 to 3.73)	3.77 (2.43 to 6.44)
3285	17	56	9238.0 (8183.3 to 10178.6)	29715.8 (28145.5 to 31298.9)	20477.8 (18623.6 to 22295.6)	3.22 (2.88 to 3.66)	4.65 (2.87 to 8.49)
3650	8	36	9986.6 (8846.2 to 11007.2)	33362.5 (31460.7 to 35220.6)	23375.9 (21356.6 to 25581.5)	3.34 (2.98 to 3.81)	6.79 (3.63 to 21.13)
4015	13	22	11856.4 (10346.5 to 13425.6)	36599.9 (34365.4 to 39057.2)	24743.5 (22282.5 to 27450.1)	3.09 (2.73 to 3.54)	2.70 (1.40 to 5.69)
4380	0	10	11856.4 (10346.5 to 13425.6)	39268.7 (36612.0 to 41950.8)	27412.3 (24292.7 to 30282.3)	3.31 (2.88 to 3.85)	***
4745	2	2	13394.8 (11104.6 to 16520.0)	40285.5 (37237.7 to 43205.0)	26890.7 (22875.6 to 30715.7)	3.01 (2.43 to 3.67)	***
5110	2	0	20541.9 (12520.0 to 34806.8)	40285.5 (37237.7 to 43205.0)	19743.5 (5091.7 to 28574.2)	1.96 (1.15 to 3.27)	***
**Subgroup: TB - Pulmonary**							
30	49	1127	31.3 (22.3 to 39.5)	717.7 (678.1 to 755.8)	686.4 (645.4 to 724.3)	22.96 (18.29 to 31.78)	23.35 (18.59 to 32.33)
90	103	817	98.1 (83.1 to 113.3)	1246.6 (1194.1 to 1300.5)	1148.5 (1095.8 to 1204.5)	12.71 (11.04 to 15.16)	8.18 (6.83 to 10.28)
180	144	662	194.5 (172.7 to 215.6)	1688.6 (1630.5 to 1752.2)	1494.1 (1432.8 to 1563.0)	8.68 (7.80 to 9.77)	4.79 (4.02 to 5.86)
365	294	944	402.4 (373.6 to 434.1)	2354.6 (2281.1 to 2431.9)	1952.3 (1873.4 to 2036.8)	5.85 (5.37 to 6.35)	3.38 (2.98 to 3.83)
730	484	1439	782.8 (739.4 to 827.2)	3482.9 (3387.4 to 3579.8)	2700.1 (2590.4 to 2799.6)	4.45 (4.16 to 4.73)	3.17 (2.86 to 3.46)
1095	486	1085	1223.9 (1166.1 to 1285.1)	4468.1 (4361.2 to 4564.1)	3244.2 (3116.3 to 3354.3)	3.65 (3.45 to 3.85)	2.41 (2.18 to 2.69)
1460	447	929	1699.1 (1623.1 to 1768.2)	5449.4 (5328.7 to 5566.4)	3750.3 (3601.2 to 3886.7)	3.21 (3.07 to 3.38)	2.27 (2.01 to 2.53)
1825	377	804	2167.6 (2083.4 to 2249.7)	6445.8 (6308.0 to 6576.1)	4278.3 (4123.2 to 4434.9)	2.97 (2.86 to 3.12)	2.35 (2.07 to 2.66)
2190	299	650	2613.3 (2514.4 to 2711.1)	7406.4 (7265.3 to 7548.7)	4793.0 (4621.1 to 4957.7)	2.83 (2.72 to 2.95)	2.41 (2.12 to 2.75)
2555	234	522	3041.4 (2932.8 to 3151.3)	8355.1 (8177.3 to 8521.7)	5313.7 (5104.6 to 5495.4)	2.75 (2.64 to 2.86)	2.50 (2.13 to 2.90)
2920	188	376	3475.8 (3348.1 to 3602.2)	9217.2 (9025.9 to 9410.1)	5741.4 (5536.1 to 5974.8)	2.65 (2.56 to 2.77)	2.26 (1.89 to 2.72)
3285	160	329	3961.0 (3809.5 to 4127.5)	10208.9 (9980.9 to 10438.0)	6247.9 (5990.5 to 6504.4)	2.58 (2.47 to 2.69)	2.34 (1.95 to 2.83)
3650	117	209	4441.6 (4267.0 to 4634.5)	11077.8 (10826.2 to 11333.9)	6636.2 (6337.2 to 6932.0)	2.49 (2.39 to 2.60)	2.05 (1.61 to 2.61)
4015	76	154	4917.9 (4718.2 to 5127.7)	12047.6 (11764.9 to 12342.9)	7129.7 (6795.4 to 7449.8)	2.45 (2.34 to 2.57)	2.34 (1.80 to 3.11)
4380	44	88	5440.1 (5183.1 to 5689.9)	13067.1 (12695.5 to 13448.9)	7627.0 (7222.4 to 8037.3)	2.40 (2.28 to 2.53)	2.33 (1.63 to 3.29)
4745	17	27	5963.7 (5589.8 to 6316.5)	13887.1 (13432.1 to 14390.7)	7923.4 (7397.1 to 8465.3)	2.33 (2.19 to 2.49)	1.86 (1.02 to 3.53)
5110	7	4	6617.0 (6033.7 to 7249.4)	14298.2 (13734.0 to 14965.0)	7681.1 (6888.3 to 8575.5)	2.16 (1.97 to 2.38)	***

**Subgroup: TB - Extrapulmonary**							
30	4	304	17.4 (2.1 to 39.0)	1318.8 (1173.3 to 1460.3)	1301.5 (1156.1 to 1444.5)	***	***
90	11	241	66.1 (35.2 to 101.3)	2382.5 (2183.3 to 2586.8)	2316.4 (2110.4 to 2519.8)	36.06 (22.83 to 67.43)	23.08 (14.26 to 50.52)
180	17	141	143.8 (98.6 to 192.7)	3025.3 (2781.6 to 3243.7)	2881.5 (2642.0 to 3103.0)	21.04 (15.45 to 31.11)	8.90 (5.56 to 16.12)
365	31	164	293.5 (227.9 to 367.8)	3813.6 (3528.1 to 4063.4)	3520.1 (3200.9 to 3783.0)	12.99 (10.26 to 16.74)	5.76 (3.88 to 9.01)
730	66	193	648.3 (546.3 to 758.8)	4843.3 (4522.7 to 5084.7)	4195.0 (3862.7 to 4464.8)	7.47 (6.35 to 9.02)	3.23 (2.50 to 4.27)
1095	60	119	1018.9 (888.8 to 1162.7)	5580.5 (5240.6 to 5885.8)	4561.5 (4180.7 to 4879.8)	5.48 (4.74 to 6.34)	2.22 (1.60 to 3.05)
1460	54	101	1410.2 (1249.3 to 1576.4)	6304.8 (5944.8 to 6668.4)	4894.7 (4502.5 to 5244.7)	4.47 (3.93 to 5.05)	2.11 (1.56 to 3.00)
1825	48	78	1815.6 (1615.2 to 2029.9)	6959.4 (6566.9 to 7337.0)	5143.8 (4696.3 to 5517.7)	3.83 (3.38 to 4.31)	1.85 (1.29 to 2.58)
2190	40	64	2221.0 (1987.4 to 2459.9)	7614.3 (7200.8 to 8000.4)	5393.2 (4889.3 to 5801.9)	3.43 (3.04 to 3.84)	1.83 (1.26 to 2.83)
2555	32	46	2621.5 (2360.4 to 2905.2)	8184.7 (7724.1 to 8614.3)	5563.1 (5082.4 to 6030.7)	3.12 (2.80 to 3.50)	1.66 (1.05 to 2.59)
2920	24	25	3000.5 (2695.4 to 3296.5)	8575.3 (8139.3 to 9036.2)	5574.8 (5083.7 to 6097.8)	2.86 (2.57 to 3.17)	1.21 (0.71 to 2.18)
3285	14	34	3296.3 (2968.7 to 3642.9)	9269.4 (8783.1 to 9763.2)	5973.0 (5438.2 to 6561.8)	2.81 (2.54 to 3.14)	2.85 (1.63 to 5.85)
3650	16	22	3740.1 (3347.2 to 4159.6)	9880.4 (9339.6 to 10404.6)	6140.3 (5563.5 to 6766.5)	2.64 (2.37 to 2.97)	1.63 (0.88 to 3.65)
4015	7	13	4037.3 (3583.5 to 4494.5)	10404.4 (9811.8 to 10968.4)	6367.1 (5689.5 to 7071.2)	2.58 (2.29 to 2.90)	2.21 (0.84 to 7.35)
4380	6	7	4592.8 (3959.1 to 5235.2)	11002.2 (10209.3 to 11749.9)	6409.4 (5470.2 to 7346.8)	2.40 (2.07 to 2.82)	***
4745	4	3	5605.0 (4554.5 to 6843.8)	11520.3 (10545.9 to 12569.9)	5915.3 (4344.1 to 7301.4)	2.06 (1.66 to 2.56)	***
5110	0	0	5605.0 (4554.5 to 6843.8)	11520.3 (10545.9 to 12569.9)	5915.3 (4344.1 to 7301.4)	2.06 (1.66 to 2.56)	***
**Subgroup: TB - Pulmonary+Extrapulmonary**							
30	1	93	19.4 (0.0 to 58.2)	1802.0 (1435.1 to 2141.1)	1782.6 (1414.0 to 2131.5)	***	***
90	2	61	58.9 (0.0 to 128.2)	3005.4 (2532.9 to 3403.4)	2946.5 (2485.7 to 3355.1)	***	***
180	5	38	161.3 (49.9 to 273.2)	3778.5 (3229.0 to 4264.6)	3617.2 (3073.3 to 4110.5)	23.42 (13.34 to 77.19)	***
365	12	41	418.6 (247.2 to 590.2)	4652.9 (4001.5 to 5193.4)	4234.3 (3586.8 to 4816.5)	11.12 (7.49 to 19.57)	8.87 (4.09 to 44.16)
730	15	60	775.9 (519.9 to 1033.9)	6076.1 (5388.8 to 6741.4)	5300.2 (4615.2 to 5985.0)	7.83 (5.72 to 12.14)	4.10 (2.22 to 9.74)
1095	12	33	1104.3 (799.0 to 1417.0)	6984.0 (6284.6 to 7708.3)	5879.7 (5127.1 to 6668.9)	6.32 (4.84 to 8.83)	4.97 (3.06 to 9.30)
1460	14	20	1543.6 (1146.7 to 1944.3)	7613.7 (6819.9 to 8335.6)	6070.2 (5234.7 to 6941.6)	4.93 (3.85 to 6.62)	3.52 (1.86 to 7.94)
1825	10	21	1929.8 (1495.4 to 2390.0)	8370.0 (7581.9 to 9224.5)	6440.2 (5504.4 to 7355.8)	4.34 (3.40 to 5.59)	1.85 (0.94 to 3.83)
2190	9	17	2344.3 (1873.7 to 2920.7)	9117.1 (8307.8 to 9988.1)	6772.8 (5779.2 to 7744.7)	3.89 (3.07 to 4.86)	2.77 (1.33 to 6.98)
2555	4	19	2572.3 (2037.2 to 3133.1)	10165.8 (9226.1 to 11151.1)	7593.4 (6397.8 to 8689.0)	3.95 (3.14 to 4.99)	2.51 (1.22 to 6.24)
2920	1	4	2640.2 (2103.3 to 3232.4)	10439.7 (9457.9 to 11483.2)	7799.5 (6554.6 to 8931.7)	3.95 (3.15 to 5.03)	***
3285	5	9	3090.6 (2462.5 to 3786.3)	11272.2 (10128.2 to 12449.2)	8181.6 (6769.6 to 9413.2)	3.65 (2.94 to 4.64)	***
3650	3	3	3492.3 (2651.0 to 4348.0)	11663.2 (10507.4 to 12913.8)	8170.9 (6661.7 to 9707.4)	3.34 (2.61 to 4.50)	***
4015	4	5	4251.6 (3186.6 to 5421.2)	12726.0 (11288.9 to 14418.9)	8474.4 (6722.5 to 10437.5)	2.99 (2.32 to 4.08)	***
4380	3	3	5352.9 (3877.3 to 7074.5)	13921.1 (11907.4 to 16253.8)	8568.2 (5895.4 to 11520.6)	2.60 (1.89 to 3.79)	***
4745	0	0	5352.9 (3877.3 to 7074.5)	13921.1 (11907.4 to 16253.8)	8568.2 (5895.4 to 11520.6)	2.60 (1.89 to 3.79)	***

5110	0	0	5352.9 (3877.3 to 7074.5)	13921.1 (11907.4 to 16253.8)	8568.2 (5895.4 to 11520.6)	2.60 (1.89 to 3.79)	***

*** IRR/RR was not estimated when there were fewer than 20 events in the risk period or fewer than 5 events in one group. Confidence intervals obtained through percentile bootstrap.

Supplementary Table 11. Estimated risk of death of natural causes (excluding external causes, TB and HIV deaths) comparing treated Tuberculosis cases to non-exposed controls by subgroup.

	Number of events		Risk per 100,000				
Time - Days	Unexposed	Exposed	Unexposed	Exposed	Risk difference per 100,000 - 95% CI	Risk ratio - 95% CI	Incidence rate ratio - 95% CI
				Subgroup: Male			
30	26	72	37.9 (24.8 to 52.5)	104.9 (82.3 to 126.8)	67.0 (40.8 to 91.8)	2.77 (1.85 to 4.24)	2.77 (1.85 to 4.25)
90	49	179	110.6 (88.4 to 135.7)	370.9 (323.6 to 416.4)	260.3 (210.0 to 313.3)	3.35 (2.65 to 4.25)	3.66 (2.68 to 5.01)
180	67	212	213.2 (182.2 to 244.4)	695.8 (633.8 to 753.5)	482.6 (415.3 to 546.0)	3.26 (2.75 to 3.86)	3.18 (2.49 to 4.09)
365	125	411	417.0 (373.9 to 465.5)	1366.4 (1272.8 to 1446.8)	949.4 (845.7 to 1039.6)	3.28 (2.89 to 3.69)	3.32 (2.75 to 3.99)
730	229	648	840.4 (773.6 to 913.1)	2565.1 (2440.4 to 2690.2)	1724.7 (1573.1 to 1861.4)	3.05 (2.75 to 3.35)	2.89 (2.49 to 3.34)
1095	238	527	1364.3 (1266.0 to 1470.0)	3725.5 (3571.8 to 3872.3)	2361.2 (2176.4 to 2533.5)	2.73 (2.49 to 2.96)	2.29 (1.95 to 2.66)
1460	212	460	1924.9 (1805.6 to 2051.1)	4951.8 (4772.3 to 5135.5)	3026.9 (2814.8 to 3246.5)	2.57 (2.39 to 2.76)	2.26 (1.97 to 2.63)
1825	179	355	2504.7 (2359.5 to 2640.8)	6105.6 (5892.2 to 6351.6)	3600.9 (3353.4 to 3850.8)	2.44 (2.28 to 2.60)	2.09 (1.76 to 2.54)
2190	109	281	2926.9 (2771.8 to 3095.2)	7207.1 (6973.2 to 7490.5)	4280.1 (4010.8 to 4558.8)	2.46 (2.32 to 2.61)	2.74 (2.19 to 3.48)
2555	93	201	3371.3 (3196.0 to 3565.0)	8167.3 (7911.1 to 8462.8)	4796.0 (4507.1 to 5099.0)	2.42 (2.27 to 2.57)	2.32 (1.85 to 2.99)
2920	77	179	3848.4 (3654.8 to 4073.4)	9272.9 (8988.7 to 9606.0)	5424.5 (5082.3 to 5790.4)	2.41 (2.27 to 2.56)	2.52 (1.98 to 3.25)
3285	68	129	4414.6 (4174.7 to 4660.3)	10338.9 (10013.2 to 10738.0)	5924.3 (5512.4 to 6328.5)	2.34 (2.19 to 2.48)	2.07 (1.62 to 2.87)
3650	40	90	4886.4 (4608.0 to 5172.8)	11413.3 (11026.9 to 11849.3)	6527.0 (6055.9 to 7008.2)	2.34 (2.19 to 2.49)	2.48 (1.77 to 3.71)
4015	26	57	5460.2 (5072.0 to 5845.1)	12579.8 (12105.0 to 13118.4)	7119.6 (6500.5 to 7729.6)	2.30 (2.14 to 2.49)	2.44 (1.61 to 4.04)
4380	12	25	6013.0 (5504.6 to 6522.9)	13640.3 (13043.4 to 14308.4)	7627.4 (6852.4 to 8488.6)	2.27 (2.07 to 2.48)	2.33 (1.18 to 5.05)
4745	7	1	7100.6 (6175.3 to 8017.2)	13742.4 (13160.0 to 14432.3)	6641.8 (5425.8 to 7753.9)	1.94 (1.68 to 2.24)	***
5110	2	0	8215.1 (6701.5 to 10128.7)	13742.4 (13160.0 to 14432.3)	5527.3 (3604.5 to 7226.3)	1.67 (1.36 to 2.07)	***
				Subgroup: Female			
30	13	34	30.5 (16.4 to 49.3)	79.8 (54.0 to 104.5)	49.3 (19.9 to 79.7)	2.62 (1.49 to 5.38)	2.62 (1.49 to 5.39)
90	16	61	68.6 (47.2 to 96.0)	225.5 (182.8 to 268.2)	156.8 (107.1 to 207.8)	3.28 (2.19 to 4.97)	3.82 (2.32 to 6.97)

180	37	87	159.6 (124.0 to 196.5)	439.2 (383.2 to 497.0)	279.6 (208.1 to 345.7)	2.75 (2.12 to 3.64)	2.36 (1.70 to 3.66)
365	47	150	281.3 (228.9 to 330.7)	826.4 (747.1 to 911.7)	545.1 (461.6 to 648.1)	2.94 (2.46 to 3.66)	3.21 (2.47 to 4.61)
730	112	232	604.0 (521.8 to 681.9)	1494.2 (1375.0 to 1610.6)	890.3 (754.9 to 1025.4)	2.47 (2.16 to 2.87)	2.10 (1.67 to 2.62)
1095	82	212	879.5 (791.1 to 980.2)	2202.6 (2052.0 to 2357.9)	1323.1 (1142.3 to 1481.8)	2.50 (2.20 to 2.83)	2.63 (2.05 to 3.44)
1460	82	181	1207.4 (1089.7 to 1328.6)	2924.8 (2743.0 to 3130.1)	1717.5 (1507.3 to 1948.8)	2.42 (2.17 to 2.71)	2.26 (1.78 to 2.98)
1825	64	129	1515.3 (1381.1 to 1656.4)	3544.9 (3318.6 to 3773.0)	2029.5 (1777.3 to 2269.1)	2.34 (2.09 to 2.58)	2.08 (1.54 to 2.81)
2190	65	120	1891.9 (1739.8 to 2070.6)	4237.2 (3977.3 to 4489.7)	2345.3 (2065.5 to 2585.1)	2.24 (2.01 to 2.47)	1.91 (1.42 to 2.58)
2555	51	99	2249.3 (2064.9 to 2472.4)	4939.9 (4658.1 to 5214.2)	2690.6 (2362.8 to 2972.0)	2.20 (1.98 to 2.40)	2.02 (1.47 to 2.75)
2920	52	67	2720.2 (2486.7 to 2979.4)	5546.9 (5224.4 to 5807.1)	2826.7 (2450.0 to 3184.0)	2.04 (1.84 to 2.23)	1.35 (0.95 to 1.94)
3285	28	62	3053.0 (2792.8 to 3337.6)	6271.4 (5881.2 to 6592.6)	3218.3 (2790.6 to 3593.9)	2.05 (1.86 to 2.25)	2.32 (1.55 to 3.84)
3650	20	37	3388.6 (3115.6 to 3691.9)	6916.1 (6494.7 to 7297.3)	3527.5 (3010.4 to 3965.8)	2.04 (1.84 to 2.24)	1.95 (1.18 to 3.56)
4015	16	21	3843.8 (3454.8 to 4210.6)	7510.8 (7041.7 to 8000.3)	3667.0 (3047.6 to 4247.6)	1.95 (1.76 to 2.19)	1.39 (0.80 to 2.89)
4380	9	13	4248.4 (3774.5 to 4754.9)	8127.0 (7581.4 to 8693.2)	3878.6 (3171.2 to 4591.1)	1.91 (1.69 to 2.17)	1.54 (0.66 to 3.88)
4745	5	4	5005.6 (4248.5 to 5894.9)	8728.7 (7959.0 to 9682.7)	3723.1 (2527.6 to 4756.7)	1.74 (1.44 to 2.09)	***
5110	0	0	5005.6 (4248.5 to 5894.9)	8728.7 (7959.0 to 9682.7)	3723.1 (2527.6 to 4756.7)	1.74 (1.44 to 2.09)	***
				**Subgroup: Age <18**			
30	0	1	0.0 (0.0 to 0.0)	14.4 (0.0 to 43.2)	14.4 (0.0 to 43.2)	***	***
90	1	11	14.7 (0.0 to 58.6)	174.9 (87.1 to 277.1)	160.2 (58.0 to 262.8)	***	***
180	0	5	14.7 (0.0 to 58.6)	249.1 (146.3 to 380.8)	234.4 (118.0 to 365.9)	***	***
365	0	8	14.7 (0.0 to 58.6)	371.6 (237.2 to 508.4)	356.9 (223.3 to 503.7)	***	***
730	1	10	33.1 (0.0 to 88.2)	541.2 (373.9 to 726.2)	508.1 (337.0 to 686.5)	***	***
1095	0	8	33.1 (0.0 to 88.2)	702.6 (517.6 to 904.7)	669.5 (485.5 to 884.9)	***	***
1460	0	6	33.1 (0.0 to 88.2)	841.9 (627.5 to 1060.0)	808.8 (592.9 to 1040.6)	***	***
1825	2	1	86.7 (14.7 to 189.3)	868.5 (647.7 to 1099.3)	781.8 (535.1 to 1022.9)	***	***
2190	1	1	118.5 (21.9 to 244.7)	900.3 (666.3 to 1144.2)	781.8 (527.3 to 1049.5)	7.60 (3.46 to 47.12)	***
2555	0	4	118.5 (21.9 to 244.7)	1034.1 (780.3 to 1319.7)	915.7 (632.0 to 1227.0)	8.73 (4.03 to 52.67)	***
2920	0	4	118.5 (21.9 to 244.7)	1223.5 (912.3 to 1573.6)	1105.0 (788.1 to 1475.1)	10.33 (4.95 to 60.32)	***
3285	1	0	185.5 (40.7 to 387.0)	1223.5 (912.3 to 1573.6)	1038.0 (688.3 to 1418.6)	6.60 (3.07 to 33.30)	***
3650	0	1	185.5 (40.7 to 387.0)	1311.2 (960.9 to 1718.7)	1125.7 (724.1 to 1524.5)	7.07 (3.30 to 36.06)	***
4015	0	1	185.5 (40.7 to 387.0)	1437.8 (1034.3 to 1889.9)	1252.3 (807.7 to 1742.1)	7.75 (3.54 to 36.57)	***
4380	0	0	185.5 (40.7 to 387.0)	1437.8 (1034.3 to 1889.9)	1252.3 (807.7 to 1742.1)	7.75 (3.54 to 36.57)	***
4745	0	0	185.5 (40.7 to 387.0)	1437.8 (1034.3 to 1889.9)	1252.3 (807.7 to 1742.1)	7.75 (3.54 to 36.57)	***
5110	0	0	185.5 (40.7 to 387.0)	1437.8 (1034.3 to 1889.9)	1252.3 (807.7 to 1742.1)	7.75 (3.54 to 36.57)	***
				**Subgroup: Age 18-59**			
30	15	53	16.2 (8.7 to 23.8)	57.3 (43.3 to 71.4)	41.1 (24.3 to 57.3)	3.53 (2.15 to 7.12)	3.53 (2.15 to 7.11)

90	21	115	39.4 (27.3 to 52.5)	184.0 (156.5 to 210.7)	144.7 (115.3 to 175.5)	4.68 (3.34 to 6.89)	5.49 (3.49 to 9.06)
180	36	157	80.2 (62.5 to 100.1)	362.3 (322.2 to 398.9)	282.1 (235.4 to 324.0)	4.52 (3.48 to 5.94)	4.38 (3.10 to 6.62)
365	69	306	163.4 (137.9 to 189.9)	731.3 (680.9 to 784.3)	567.9 (509.7 to 625.1)	4.48 (3.69 to 5.43)	4.47 (3.44 to 5.78)
730	137	489	349.4 (308.9 to 390.7)	1396.6 (1320.6 to 1474.8)	1047.2 (958.1 to 1139.0)	4.00 (3.54 to 4.60)	3.62 (3.10 to 4.45)
1095	132	422	560.9 (504.7 to 618.2)	2068.8 (1968.9 to 2166.2)	1507.9 (1398.2 to 1627.3)	3.69 (3.32 to 4.21)	3.27 (2.70 to 4.07)
1460	122	360	793.5 (722.1 to 860.9)	2758.8 (2640.6 to 2875.5)	1965.3 (1838.8 to 2101.4)	3.48 (3.17 to 3.84)	3.05 (2.51 to 3.77)
1825	117	282	1065.8 (977.5 to 1151.7)	3418.2 (3278.6 to 3571.6)	2352.4 (2194.8 to 2513.2)	3.21 (2.94 to 3.53)	2.51 (2.01 to 3.12)
2190	85	227	1303.8 (1193.3 to 1396.7)	4058.5 (3906.9 to 4240.6)	2754.7 (2579.6 to 2971.6)	3.11 (2.86 to 3.42)	2.80 (2.23 to 3.73)
2555	67	176	1533.4 (1416.2 to 1653.3)	4667.5 (4496.6 to 4864.2)	3134.2 (2939.0 to 3387.6)	3.04 (2.80 to 3.33)	2.77 (2.09 to 3.78)
2920	66	161	1821.5 (1684.9 to 1959.2)	5380.9 (5179.2 to 5614.2)	3559.5 (3334.4 to 3808.0)	2.95 (2.72 to 3.21)	2.59 (1.98 to 3.49)
3285	63	128	2183.3 (2015.7 to 2343.2)	6126.2 (5862.8 to 6395.4)	3942.8 (3654.1 to 4253.4)	2.81 (2.59 to 3.05)	2.18 (1.68 to 3.00)
3650	37	90	2491.6 (2301.4 to 2677.6)	6881.5 (6593.8 to 7199.4)	4389.9 (4013.8 to 4735.1)	2.76 (2.52 to 3.00)	2.62 (1.86 to 3.92)
4015	27	56	2898.6 (2651.6 to 3138.9)	7702.3 (7342.9 to 8078.0)	4803.7 (4344.5 to 5262.7)	2.66 (2.41 to 2.93)	2.25 (1.45 to 3.58)
4380	11	23	3231.4 (2922.7 to 3532.2)	8320.8 (7887.3 to 8758.4)	5089.3 (4570.7 to 5687.5)	2.57 (2.31 to 2.90)	2.27 (1.09 to 5.27)
4745	7	3	3901.5 (3352.2 to 4514.2)	8553.5 (8058.3 to 9118.6)	4652.0 (3848.2 to 5426.6)	2.19 (1.87 to 2.58)	***
5110	2	0	4453.0 (3573.9 to 5431.6)	8553.5 (8058.3 to 9118.6)	4100.6 (3062.5 to 5092.8)	1.92 (1.57 to 2.38)	***
**Subgroup: Age ≥60**							
30	24	52	202.8 (126.7 to 287.7)	439.2 (316.3 to 565.8)	236.4 (76.2 to 397.2)	2.17 (1.29 to 3.97)	2.17 (1.29 to 3.98)
90	43	114	572.4 (452.2 to 717.6)	1421.8 (1214.1 to 1661.1)	849.5 (591.7 to 1125.4)	2.48 (1.89 to 3.26)	2.67 (1.90 to 3.67)
180	68	137	1177.9 (1010.0 to 1404.5)	2643.6 (2359.2 to 2947.9)	1465.7 (1123.6 to 1797.1)	2.24 (1.86 to 2.70)	2.04 (1.54 to 2.75)
365	103	247	2156.2 (1910.3 to 2419.0)	4985.0 (4641.0 to 5364.8)	2828.8 (2407.4 to 3263.8)	2.31 (2.02 to 2.66)	2.46 (1.99 to 3.14)
730	203	381	4350.1 (3958.8 to 4735.1)	9083.1 (8605.7 to 9576.0)	4733.0 (4089.6 to 5381.9)	2.09 (1.89 to 2.33)	1.96 (1.65 to 2.34)
1095	188	309	6798.6 (6329.9 to 7306.7)	13103.3 (12437.6 to 13749.6)	6304.7 (5438.3 to 7147.7)	1.93 (1.76 to 2.10)	1.75 (1.47 to 2.11)
1460	172	275	9619.0 (8979.7 to 10264.4)	17605.7 (16788.1 to 18377.1)	7986.7 (6823.1 to 8910.1)	1.83 (1.68 to 1.99)	1.73 (1.41 to 2.07)
1825	124	201	12149.6 (11476.1 to 12931.1)	21653.4 (20600.2 to 22657.3)	9503.8 (8172.3 to 10625.5)	1.78 (1.65 to 1.91)	1.80 (1.45 to 2.29)
2190	88	173	14324.7 (13484.2 to 15195.8)	25888.1 (24681.1 to 27024.5)	11563.4 (10225.2 to 12983.7)	1.81 (1.68 to 1.94)	2.24 (1.75 to 2.96)
2555	77	120	16725.3 (15751.8 to 17782.9)	29560.0 (28220.2 to 30813.9)	12834.7 (11085.1 to 14551.0)	1.77 (1.64 to 1.90)	1.82 (1.34 to 2.38)
2920	63	81	19443.9 (18333.1 to 20649.7)	32869.8 (31494.9 to 34245.7)	13425.9 (11382.3 to 15350.8)	1.69 (1.56 to 1.82)	1.54 (1.13 to 2.11)
3285	32	63	21489.0 (20271.9 to 22856.2)	36520.6 (35008.4 to 38108.3)	15031.7 (12856.5 to 17040.1)	1.70 (1.57 to 1.82)	2.40 (1.63 to 3.84)
3650	23	36	23537.4 (22024.4 to 25013.8)	39744.9 (38079.2 to 41626.8)	16207.4 (13918.7 to 18396.7)	1.69 (1.56 to 1.81)	1.94 (1.21 to 3.33)
4015	15	21	25802.3 (23943.4 to 27705.3)	42542.3 (40561.2 to 44727.2)	16740.0 (14178.6 to 19389.1)	1.65 (1.53 to 1.79)	1.77 (0.92 to 3.32)
4380	10	15	28113.8 (25890.1 to 30664.2)	46243.3 (44007.9 to 48913.7)	18129.5 (14932.8 to 21224.5)	1.64 (1.49 to 1.80)	1.96 (0.78 to 5.29)
4745	5	2	31847.0 (28168.5 to 35998.8)	48491.4 (45093.7 to 53302.3)	16644.4 (11325.9 to 23268.1)	1.52 (1.32 to 1.81)	***
5110	0	0	31847.0 (28168.5 to 35998.8)	48491.4 (45093.7 to 53302.3)	16644.4 (11325.9 to 23268.1)	1.52 (1.32 to 1.81)	***
**Subgroup: White**							

30	12	33	42.0 (21.0 to 66.5)	115.7 (75.3 to 157.7)	73.6 (28.1 to 119.2)	2.75 (1.50 to 5.66)	2.75 (1.50 to 5.65)
90	15	71	95.5 (63.5 to 131.4)	369.7 (300.8 to 436.2)	274.1 (197.4 to 345.7)	3.87 (2.64 to 5.94)	4.75 (2.94 to 9.21)
180	25	87	188.0 (143.9 to 238.6)	691.8 (606.5 to 787.0)	503.8 (395.4 to 614.0)	3.68 (2.73 to 4.96)	3.50 (2.29 to 5.82)
365	44	144	362.0 (297.7 to 440.8)	1259.1 (1119.0 to 1407.7)	897.1 (741.4 to 1054.0)	3.48 (2.80 to 4.40)	3.31 (2.47 to 4.67)
730	90	268	767.5 (662.9 to 886.0)	2466.3 (2266.4 to 2674.8)	1698.7 (1473.1 to 1916.0)	3.21 (2.77 to 3.78)	3.04 (2.42 to 3.83)
1095	86	199	1235.9 (1097.2 to 1387.8)	3549.2 (3311.1 to 3790.0)	2313.3 (2028.7 to 2606.9)	2.87 (2.50 to 3.29)	2.39 (1.87 to 3.12)
1460	84	170	1799.0 (1626.4 to 1994.4)	4694.2 (4397.0 to 4998.1)	2895.2 (2574.0 to 3217.3)	2.61 (2.32 to 2.94)	2.10 (1.65 to 2.78)
1825	63	130	2327.6 (2089.8 to 2547.8)	5787.3 (5437.8 to 6133.0)	3459.7 (3045.0 to 3851.3)	2.49 (2.23 to 2.78)	2.17 (1.57 to 3.04)
2190	37	124	2705.0 (2424.7 to 2950.4)	7061.9 (6654.7 to 7474.2)	4356.9 (3867.6 to 4826.0)	2.61 (2.36 to 2.94)	3.57 (2.59 to 5.29)
2555	31	60	3090.5 (2783.4 to 3371.1)	7820.2 (7376.8 to 8280.4)	4729.7 (4232.9 to 5222.3)	2.53 (2.29 to 2.81)	2.09 (1.43 to 3.33)
2920	34	66	3649.3 (3306.6 to 3992.5)	8902.4 (8437.0 to 9428.0)	5253.1 (4663.4 to 5865.3)	2.44 (2.19 to 2.72)	2.11 (1.43 to 3.38)
3285	19	44	4049.1 (3635.3 to 4461.6)	9842.8 (9313.9 to 10454.6)	5793.7 (5116.1 to 6485.3)	2.43 (2.19 to 2.72)	2.55 (1.49 to 4.45)
3650	19	39	4625.7 (4115.6 to 5098.5)	11018.5 (10373.8 to 11629.5)	6392.8 (5645.9 to 7142.6)	2.38 (2.15 to 2.72)	2.28 (1.36 to 4.18)
4015	5	14	4890.4 (4312.1 to 5422.6)	11760.0 (11018.1 to 12528.2)	6869.6 (5998.6 to 7795.3)	2.40 (2.14 to 2.75)	***
4380	2	8	5040.3 (4435.8 to 5549.8)	12547.4 (11641.1 to 13510.7)	7507.1 (6380.5 to 8582.8)	2.49 (2.19 to 2.88)	***
4745	1	1	5249.6 (4581.8 to 5973.8)	12746.6 (11720.4 to 13852.2)	7497.0 (6273.5 to 8740.4)	2.43 (2.08 to 2.85)	***
5110	0	0	5249.6 (4581.8 to 5973.8)	12746.6 (11720.4 to 13852.2)	7497.0 (6273.5 to 8740.4)	2.43 (2.08 to 2.85)	***
				**Subgroup: Black**			
30	4	11	33.1 (8.3 to 58.1)	91.2 (41.5 to 149.1)	58.0 (-4.3 to 120.3)	***	***
90	8	23	100.5 (50.2 to 150.9)	285.2 (192.8 to 377.8)	184.7 (83.7 to 289.7)	2.84 (1.61 to 5.86)	2.88 (1.41 to 8.52)
180	12	38	204.6 (126.9 to 282.6)	614.9 (476.1 to 765.9)	410.3 (264.5 to 591.8)	3.01 (2.02 to 5.40)	3.18 (1.82 to 7.50)
365	23	56	414.0 (304.0 to 520.2)	1126.3 (935.4 to 1331.0)	712.3 (523.2 to 939.7)	2.72 (2.06 to 3.87)	2.46 (1.55 to 4.30)
730	38	116	803.1 (641.2 to 966.1)	2309.5 (2053.3 to 2614.5)	1506.3 (1216.8 to 1825.9)	2.88 (2.33 to 3.74)	3.11 (2.29 to 4.71)
1095	37	90	1248.7 (1043.6 to 1452.5)	3378.2 (3066.6 to 3734.5)	2129.6 (1758.5 to 2520.7)	2.71 (2.24 to 3.25)	2.51 (1.77 to 3.77)
1460	26	78	1613.0 (1356.1 to 1895.2)	4479.7 (4101.1 to 4876.0)	2866.7 (2419.3 to 3350.3)	2.78 (2.33 to 3.32)	3.13 (2.08 to 5.30)
1825	37	59	2236.3 (1908.4 to 2569.3)	5472.1 (4991.1 to 5956.3)	3235.8 (2682.3 to 3821.3)	2.45 (2.08 to 2.94)	1.68 (1.14 to 2.59)
2190	27	45	2770.8 (2369.8 to 3184.7)	6379.0 (5796.7 to 6913.6)	3608.2 (2952.0 to 4245.3)	2.30 (1.97 to 2.74)	1.76 (1.10 to 3.10)
2555	24	44	3355.2 (2863.3 to 3854.5)	7439.9 (6814.4 to 8012.1)	4084.7 (3409.9 to 4811.7)	2.22 (1.92 to 2.62)	1.95 (1.32 to 3.21)
2920	12	28	3731.4 (3149.3 to 4233.2)	8318.3 (7675.4 to 9006.8)	4586.9 (3792.2 to 5363.6)	2.23 (1.93 to 2.62)	2.49 (1.38 to 6.03)
3285	12	25	4240.9 (3589.1 to 4800.1)	9300.2 (8537.3 to 10035.8)	5059.3 (4171.0 to 5948.9)	2.19 (1.90 to 2.58)	2.24 (1.15 to 6.13)
3650	3	18	4405.8 (3752.5 to 5011.4)	10356.7 (9471.1 to 11165.0)	5951.0 (4962.2 to 7003.9)	2.35 (2.04 to 2.80)	***
4015	5	11	4908.2 (4126.0 to 5662.4)	11487.6 (10402.8 to 12515.1)	6579.4 (5353.4 to 7962.1)	2.34 (2.01 to 2.87)	***
4380	2	5	5248.9 (4435.3 to 6234.9)	12525.6 (11148.7 to 14039.1)	7276.7 (5714.7 to 8977.4)	2.39 (1.96 to 2.92)	***
4745	1	1	5989.4 (4611.4 to 7905.6)	13396.4 (11504.2 to 15810.6)	7406.9 (4442.9 to 10221.6)	2.24 (1.58 to 3.10)	***
5110	1	0	7532.2 (4880.5 to 11298.3)	13396.4 (11504.2 to 15810.6)	5864.2 (1264.6 to 9792.5)	1.78 (1.12 to 2.92)	***

**Subgroup: Mixed**							
30	23	62	33.2 (21.6 to 47.6)	89.4 (65.6 to 112.5)	56.2 (30.2 to 82.2)	2.69 (1.71 to 4.45)	2.70 (1.72 to 4.45)
90	42	142	94.8 (73.6 to 119.4)	297.9 (258.6 to 341.0)	203.1 (157.0 to 248.4)	3.14 (2.39 to 4.17)	3.39 (2.44 to 4.77)
180	67	174	196.0 (169.6 to 236.6)	560.8 (508.3 to 611.8)	364.8 (299.2 to 427.2)	2.86 (2.34 to 3.44)	2.61 (1.95 to 3.41)
365	104	358	362.4 (323.8 to 411.8)	1133.5 (1049.1 to 1219.7)	771.1 (673.2 to 859.6)	3.13 (2.70 to 3.54)	3.47 (2.81 to 4.32)
730	210	493	738.8 (678.6 to 809.1)	2017.8 (1908.0 to 2133.2)	1279.0 (1136.9 to 1407.9)	2.73 (2.44 to 3.02)	2.39 (2.05 to 2.83)
1095	194	445	1145.7 (1057.9 to 1231.4)	2952.3 (2808.2 to 3088.8)	1806.6 (1645.3 to 1958.6)	2.58 (2.35 to 2.79)	2.35 (1.97 to 2.88)
1460	184	393	1604.5 (1490.1 to 1711.1)	3936.9 (3743.8 to 4110.2)	2332.4 (2125.9 to 2517.3)	2.45 (2.27 to 2.65)	2.20 (1.87 to 2.61)
1825	142	294	2031.5 (1896.1 to 2158.3)	4823.7 (4616.7 to 5000.8)	2792.2 (2532.3 to 3015.7)	2.37 (2.18 to 2.55)	2.15 (1.75 to 2.65)
2190	106	230	2413.0 (2275.4 to 2548.1)	5654.3 (5447.9 to 5863.3)	3241.2 (2974.2 to 3484.8)	2.34 (2.18 to 2.51)	2.27 (1.82 to 2.89)
2555	89	194	2805.0 (2649.5 to 2972.9)	6512.1 (6274.7 to 6746.7)	3707.1 (3425.4 to 3998.6)	2.32 (2.17 to 2.48)	2.30 (1.81 to 3.03)
2920	82	151	3269.9 (3069.2 to 3464.0)	7365.7 (7103.9 to 7624.0)	4095.7 (3752.2 to 4383.1)	2.25 (2.10 to 2.41)	1.95 (1.52 to 2.58)
3285	65	122	3764.6 (3537.0 to 3976.0)	8287.8 (7988.8 to 8574.5)	4523.2 (4136.4 to 4900.8)	2.20 (2.06 to 2.36)	2.00 (1.49 to 2.73)
3650	38	69	4174.8 (3917.4 to 4446.6)	9054.1 (8721.7 to 9388.0)	4879.3 (4448.0 to 5266.6)	2.17 (2.01 to 2.32)	1.94 (1.39 to 2.93)
4015	32	53	4804.8 (4458.7 to 5179.5)	10019.8 (9559.9 to 10496.4)	5215.0 (4686.9 to 5700.1)	2.09 (1.92 to 2.25)	1.79 (1.15 to 2.95)
4380	16	25	5411.6 (4955.4 to 5856.2)	10857.1 (10251.1 to 11422.6)	5445.5 (4747.3 to 6092.4)	2.01 (1.82 to 2.21)	1.69 (0.95 to 3.21)
4745	10	3	6658.0 (5706.3 to 7660.5)	11187.0 (10557.6 to 11920.1)	4529.1 (3387.4 to 5562.4)	1.68 (1.46 to 1.95)	***
5110	1	0	7054.2 (5918.3 to 8208.9)	11187.0 (10557.6 to 11920.1)	4132.8 (2835.9 to 5452.2)	1.59 (1.34 to 1.89)	***
**Subgroup: HIV**							
30	2	5	33.3 (0.0 to 83.5)	83.4 (16.7 to 166.6)	50.1 (-33.4 to 133.6)	***	***
90	2	5	67.2 (16.6 to 134.6)	168.7 (75.5 to 270.4)	101.6 (-16.6 to 219.5)	***	***
180	5	13	154.9 (68.4 to 267.2)	397.5 (241.3 to 552.8)	242.6 (52.2 to 414.2)	2.57 (1.25 to 5.94)	***
365	6	22	268.1 (141.3 to 425.7)	808.2 (590.3 to 1064.8)	540.2 (246.7 to 817.8)	3.02 (1.72 to 5.73)	3.80 (1.50 to 13.74)
730	10	35	477.3 (315.9 to 676.1)	1550.2 (1241.6 to 1851.7)	1072.9 (700.3 to 1439.8)	3.25 (2.11 to 5.18)	3.76 (1.97 to 9.47)
1095	14	24	826.7 (573.5 to 1119.5)	2170.1 (1821.8 to 2591.7)	1343.5 (872.8 to 1794.2)	2.63 (1.83 to 3.81)	1.91 (1.03 to 4.12)
1460	11	25	1167.6 (841.0 to 1496.6)	2964.8 (2477.7 to 3440.5)	1797.2 (1161.9 to 2387.8)	2.54 (1.81 to 3.58)	2.65 (1.38 to 6.11)
1825	9	17	1518.8 (1120.8 to 1934.3)	3625.4 (3050.2 to 4210.9)	2106.6 (1385.6 to 2741.2)	2.39 (1.75 to 3.25)	2.29 (1.11 to 5.50)
2190	2	11	1617.6 (1196.6 to 2093.8)	4173.2 (3508.9 to 4793.0)	2555.7 (1721.7 to 3300.8)	2.58 (1.90 to 3.54)	***
2555	4	10	1854.8 (1415.6 to 2354.9)	4806.0 (4034.5 to 5549.7)	2951.3 (2042.4 to 3830.3)	2.59 (1.91 to 3.43)	***
2920	3	12	2073.4 (1564.0 to 2691.6)	5804.8 (4783.0 to 6757.3)	3731.4 (2510.0 to 4791.4)	2.80 (2.03 to 3.81)	***
3285	3	6	2356.0 (1760.4 to 3022.4)	6481.1 (5510.2 to 7646.5)	4125.1 (2900.5 to 5420.2)	2.75 (2.04 to 3.81)	***
3650	2	4	2650.8 (2010.6 to 3440.8)	7187.0 (5945.1 to 8593.6)	4536.2 (3107.6 to 6053.0)	2.71 (1.98 to 3.75)	***
4015	1	1	2977.3 (2067.7 to 4015.0)	7432.8 (6045.4 to 9049.3)	4455.5 (2832.5 to 6038.9)	2.50 (1.73 to 3.52)	***
4380	0	1	2977.3 (2067.7 to 4015.0)	7957.4 (6323.5 to 9795.7)	4980.1 (3070.1 to 6836.2)	2.67 (1.85 to 3.92)	***
4745	0	0	2977.3 (2067.7 to 4015.0)	7957.4 (6323.5 to 9795.7)	4980.1 (3070.1 to 6836.2)	2.67 (1.85 to 3.92)	***

5110	0	0	2977.3 (2067.7 to 4015.0)	7957.4 (6323.5 to 9795.7)	4980.1 (3070.1 to 6836.2)	2.67 (1.85 to 3.92)	***
**Subgroup: Diabetes**							
30	7	18	119.4 (34.2 to 221.5)	306.8 (187.1 to 443.7)	187.4 (17.7 to 350.6)	2.57 (1.11 to 9.13)	2.57 (1.11 to 9.19)
90	5	37	205.6 (102.6 to 309.1)	948.6 (723.5 to 1206.1)	743.0 (466.5 to 1042.5)	4.61 (2.68 to 10.68)	7.44 (3.40 to 39.78)
180	12	38	422.3 (264.6 to 578.3)	1634.6 (1319.3 to 1985.0)	1212.3 (878.4 to 1650.7)	3.87 (2.65 to 6.72)	3.20 (1.92 to 6.90)
365	30	90	1008.3 (758.8 to 1241.7)	3372.3 (2931.5 to 3882.1)	2364.0 (1858.6 to 2939.5)	3.34 (2.58 to 4.46)	3.07 (2.01 to 5.01)
730	37	112	1842.2 (1505.5 to 2248.7)	5909.6 (5261.0 to 6520.0)	4067.4 (3316.3 to 4799.8)	3.21 (2.53 to 4.06)	3.13 (2.14 to 4.78)
1095	32	103	2747.5 (2284.3 to 3241.2)	8746.4 (7833.4 to 9631.7)	5998.9 (4919.2 to 7034.3)	3.18 (2.60 to 3.89)	3.39 (2.28 to 5.49)
1460	34	108	3887.0 (3256.1 to 4483.4)	12455.7 (11500.1 to 13574.3)	8568.7 (7435.6 to 9831.3)	3.20 (2.75 to 3.86)	3.43 (2.44 to 5.45)
1825	28	78	5073.1 (4276.2 to 5834.7)	15782.9 (14546.0 to 17149.4)	10709.8 (9354.8 to 12203.6)	3.11 (2.66 to 3.69)	3.10 (2.10 to 4.77)
2190	29	69	6619.3 (5645.0 to 7625.3)	19466.2 (18043.7 to 20869.0)	12846.9 (11194.4 to 14468.7)	2.94 (2.52 to 3.49)	2.73 (1.85 to 4.47)
2555	17	45	7788.3 (6712.6 to 8876.1)	22608.1 (21099.2 to 24284.1)	14819.7 (12988.6 to 16713.6)	2.90 (2.54 to 3.41)	3.14 (1.92 to 5.83)
2920	16	40	9349.1 (8148.3 to 10758.7)	26396.9 (24509.8 to 28280.4)	17047.8 (14961.0 to 19269.3)	2.82 (2.44 to 3.29)	3.04 (1.79 to 5.73)
3285	4	29	9863.5 (8542.1 to 11211.9)	30315.9 (28136.1 to 32534.5)	20452.4 (18034.4 to 23211.0)	3.07 (2.65 to 3.59)	***
3650	6	19	11088.9 (9513.4 to 12942.5)	34223.7 (31600.9 to 36932.7)	23134.9 (20111.0 to 26501.3)	3.09 (2.62 to 3.72)	4.38 (2.06 to 15.78)
4015	1	9	11425.4 (9693.4 to 13292.6)	37498.1 (34021.9 to 40799.1)	26072.8 (22415.1 to 29871.2)	3.28 (2.78 to 4.02)	***
4380	1	3	12026.9 (10068.7 to 14147.4)	39888.4 (35833.3 to 43727.5)	27861.5 (23525.2 to 32333.2)	3.32 (2.75 to 4.07)	***
4745	2	0	16820.3 (11291.2 to 23936.3)	39888.4 (35833.3 to 43727.5)	23068.1 (15300.4 to 30774.3)	2.37 (1.64 to 3.65)	***
5110	1	0	24314.2 (11662.6 to 41880.7)	39888.4 (35833.3 to 43727.5)	15574.2 (-1745.0 to 29991.1)	1.64 (0.96 to 3.53)	***
**Subgroup: TB - Pulmonary**							
30	35	89	36.5 (24.5 to 50.1)	92.9 (74.6 to 112.8)	56.4 (34.5 to 78.9)	2.54 (1.78 to 3.90)	2.54 (1.77 to 3.91)
90	57	204	97.0 (77.5 to 116.9)	310.0 (277.2 to 343.8)	212.9 (175.4 to 249.2)	3.19 (2.55 to 4.11)	3.59 (2.73 to 4.84)
180	87	259	192.4 (165.8 to 221.6)	593.8 (545.7 to 642.2)	401.4 (346.5 to 458.6)	3.09 (2.61 to 3.68)	2.99 (2.38 to 3.82)
365	158	496	376.1 (341.8 to 418.5)	1169.8 (1101.7 to 1237.3)	793.6 (712.9 to 872.6)	3.11 (2.78 to 3.47)	3.17 (2.68 to 3.82)
730	293	764	759.3 (697.3 to 815.7)	2169.1 (2069.1 to 2265.6)	1409.8 (1292.9 to 1515.9)	2.86 (2.63 to 3.14)	2.65 (2.34 to 3.07)
1095	277	658	1186.5 (1113.5 to 1262.8)	3182.8 (3054.7 to 3308.7)	1996.3 (1842.9 to 2134.7)	2.68 (2.49 to 2.89)	2.44 (2.12 to 2.81)
1460	253	580	1652.3 (1563.6 to 1749.9)	4257.5 (4104.4 to 4396.3)	2605.1 (2428.9 to 2769.0)	2.58 (2.42 to 2.75)	2.37 (2.02 to 2.76)
1825	220	441	2144.8 (2030.1 to 2258.2)	5245.6 (5057.9 to 5407.8)	3100.7 (2881.1 to 3302.1)	2.45 (2.30 to 2.60)	2.09 (1.77 to 2.48)
2190	159	364	2571.5 (2447.1 to 2695.7)	6226.8 (6020.0 to 6414.4)	3655.4 (3412.0 to 3875.2)	2.42 (2.28 to 2.57)	2.41 (2.03 to 2.93)
2555	123	281	2974.4 (2822.2 to 3124.5)	7153.8 (6908.7 to 7357.0)	4179.4 (3910.9 to 4431.4)	2.41 (2.28 to 2.55)	2.42 (2.02 to 3.03)
2920	116	220	3465.9 (3280.8 to 3634.6)	8084.0 (7813.0 to 8330.2)	4618.1 (4315.7 to 4937.1)	2.33 (2.21 to 2.48)	2.03 (1.64 to 2.60)
3285	87	171	3955.7 (3733.9 to 4156.0)	9034.6 (8712.6 to 9297.8)	5079.0 (4734.9 to 5424.7)	2.28 (2.16 to 2.42)	2.11 (1.68 to 2.70)
3650	52	116	4370.8 (4125.0 to 4619.6)	9980.2 (9624.3 to 10325.1)	5609.4 (5221.2 to 5993.9)	2.28 (2.15 to 2.44)	2.41 (1.72 to 3.36)
4015	38	68	4915.2 (4628.2 to 5218.7)	10907.3 (10518.5 to 11337.7)	5992.1 (5549.7 to 6468.3)	2.22 (2.08 to 2.39)	1.95 (1.33 to 2.97)
4380	17	35	5369.2 (4990.5 to 5737.6)	11804.0 (11316.6 to 12374.5)	6434.8 (5821.7 to 7146.7)	2.20 (2.03 to 2.40)	2.25 (1.30 to 4.62)

4745	10	4	6261.0 (5638.1 to 6942.3)	12180.8 (11588.3 to 12878.8)	5919.8 (5055.6 to 6857.8)	1.95 (1.74 to 2.18)	***
5110	2	0	6799.3 (5885.9 to 7784.4)	12180.8 (11588.3 to 12878.8)	5381.5 (4231.5 to 6474.7)	1.79 (1.55 to 2.09)	***
				**Subgroup: TB - Extrapulmonary**			
30	3	15	23.0 (0.0 to 46.2)	114.9 (61.4 to 176.3)	91.9 (30.6 to 153.3)	***	***
90	3	30	46.6 (11.5 to 92.5)	349.4 (248.9 to 451.2)	302.8 (194.7 to 419.9)	7.50 (3.57 to 33.38)	***
180	13	31	150.9 (87.1 to 222.3)	599.4 (474.7 to 733.5)	448.5 (299.3 to 614.3)	3.97 (2.53 to 7.33)	2.40 (1.32 to 5.03)
365	14	54	269.6 (176.3 to 375.7)	1060.9 (901.3 to 1251.7)	791.3 (602.3 to 1003.7)	3.94 (2.77 to 6.18)	3.90 (2.23 to 7.78)
730	38	86	635.2 (489.5 to 772.2)	1886.3 (1660.5 to 2161.4)	1251.0 (989.8 to 1564.2)	2.97 (2.34 to 4.07)	2.30 (1.65 to 3.48)
1095	35	62	1037.0 (826.3 to 1235.2)	2596.6 (2290.9 to 2905.9)	1559.5 (1205.5 to 1936.9)	2.50 (2.05 to 3.22)	1.82 (1.22 to 2.70)
1460	37	50	1549.8 (1302.8 to 1801.0)	3288.4 (2930.6 to 3631.7)	1738.6 (1292.2 to 2160.9)	2.12 (1.77 to 2.59)	1.40 (0.91 to 2.15)
1825	18	36	1854.7 (1563.1 to 2141.0)	3909.3 (3482.9 to 4280.9)	2054.6 (1558.7 to 2501.4)	2.11 (1.76 to 2.54)	2.07 (1.16 to 3.66)
2190	14	30	2139.7 (1831.3 to 2457.0)	4526.6 (4093.8 to 4917.3)	2386.9 (1851.5 to 2877.3)	2.12 (1.79 to 2.48)	2.24 (1.27 to 4.55)
2555	18	15	2600.5 (2234.5 to 2963.8)	4895.2 (4417.2 to 5355.3)	2294.7 (1703.1 to 2885.1)	1.88 (1.59 to 2.25)	0.87 (0.40 to 1.85)
2920	12	22	2999.8 (2567.9 to 3433.9)	5617.8 (5073.8 to 6158.9)	2618.0 (1928.6 to 3310.1)	1.87 (1.59 to 2.22)	1.93 (1.00 to 4.64)
3285	9	17	3404.8 (2907.9 to 3936.7)	6376.8 (5721.3 to 7044.7)	2972.0 (2168.8 to 3737.4)	1.87 (1.57 to 2.21)	2.01 (0.88 to 5.08)
3650	5	10	3715.4 (3193.5 to 4320.3)	6999.9 (6209.2 to 7722.8)	3284.4 (2335.9 to 4112.2)	1.88 (1.55 to 2.19)	***
4015	3	7	4086.6 (3512.4 to 4826.3)	7787.1 (6846.7 to 8793.3)	3700.5 (2512.8 to 4839.0)	1.91 (1.55 to 2.29)	***
4380	2	2	4406.7 (3684.7 to 5259.4)	8199.5 (7145.2 to 9455.1)	3792.8 (2401.9 to 5169.2)	1.86 (1.48 to 2.30)	***
4745	1	1	4917.0 (3841.2 to 6249.5)	8666.6 (7441.8 to 10295.4)	3749.6 (1833.5 to 5491.0)	1.76 (1.31 to 2.36)	***
5110	0	0	4917.0 (3841.2 to 6249.5)	8666.6 (7441.8 to 10295.4)	3749.6 (1833.5 to 5491.0)	1.76 (1.31 to 2.36)	***
				**Subgroup: TB - Pulmonary+Extrapulmonary**			
30	1	2	41.3 (0.0 to 165.1)	82.7 (0.0 to 207.3)	41.4 (-82.9 to 206.9)	***	***
90	5	6	251.9 (83.4 to 461.3)	335.1 (125.4 to 565.8)	83.1 (-252.4 to 418.0)	***	***
180	4	9	424.6 (171.2 to 702.8)	724.9 (423.1 to 1063.2)	300.3 (-126.1 to 731.2)	1.71 (0.81 to 4.48)	***
365	0	11	424.6 (171.2 to 702.8)	1224.9 (787.4 to 1670.2)	800.3 (267.3 to 1320.9)	2.89 (1.50 to 7.31)	***
730	10	30	938.7 (548.7 to 1369.2)	2761.1 (2122.4 to 3409.5)	1822.4 (994.9 to 2566.5)	2.94 (1.85 to 4.95)	3.10 (1.52 to 7.41)
1095	8	19	1424.6 (944.8 to 1940.2)	3894.4 (3055.2 to 4689.1)	2469.8 (1539.2 to 3453.1)	2.73 (1.87 to 4.23)	2.49 (1.16 to 7.52)
1460	4	11	1716.9 (1127.9 to 2323.1)	4690.4 (3797.6 to 5630.0)	2973.5 (1871.0 to 3919.5)	2.73 (1.92 to 4.14)	***
1825	5	7	2167.8 (1523.1 to 2877.0)	5319.0 (4264.0 to 6384.2)	3151.1 (1819.3 to 4157.8)	2.45 (1.71 to 3.46)	***
2190	1	7	2271.5 (1633.2 to 2976.0)	6083.6 (4841.5 to 7249.3)	3812.2 (2282.4 to 4951.6)	2.68 (1.87 to 3.92)	***
2555	3	4	2654.1 (1861.8 to 3522.5)	6594.1 (5247.9 to 7858.6)	3940.0 (2397.3 to 5262.1)	2.48 (1.74 to 3.55)	***
2920	1	4	2836.3 (1981.0 to 3707.9)	7300.3 (5872.4 to 8728.0)	4464.0 (2668.9 to 6002.2)	2.57 (1.78 to 3.85)	***
3285	0	3	2836.3 (1981.0 to 3707.9)	8015.4 (6389.6 to 9599.9)	5179.1 (3287.2 to 6892.0)	2.83 (1.98 to 4.28)	***
3650	3	1	3792.8 (2518.7 to 5124.8)	8352.7 (6633.3 to 10054.8)	4559.9 (2191.1 to 6625.0)	2.20 (1.46 to 3.46)	***
4015	1	3	4245.6 (2780.2 to 5976.1)	9872.9 (7540.5 to 12396.7)	5627.3 (2586.0 to 8519.1)	2.33 (1.49 to 3.68)	***

4380	2	1	6293.9 (3499.8 to 10034.0)	10908.4 (8014.5 to 14446.3)	4614.6 (39.6 to 9461.8)	1.73 (1.00 to 3.31)	***
4745	1	0	9722.5 (4212.8 to 18394.1)	10908.4 (8014.5 to 14446.3)	1185.9 (-8230.4 to 7477.5)	1.12 (0.54 to 2.59)	***
5110	0	0	9722.5 (4212.8 to 18394.1)	10908.4 (8014.5 to 14446.3)	1185.9 (-8230.4 to 7477.5)	1.12 (0.54 to 2.59)	***

*** IRR/RR was not estimated when there were fewer than 20 events in the risk period or fewer than 5 events in one group. Confidence intervals obtained through percentile bootstrap.

Supplementary Table 12. Estimated risk of death by cause-specific (defined by ICD-10 chapters) comparing diagnosed Tuberculosis cases to non-exposed controls in Females.

	Number of events		Risk per 100,000				
Time - Days	Unexposed	Exposed	Unexposed	Exposed	Risk difference per 100,000 - 95% CI	Risk ratio - 95% CI	Incidence rate ratio - 95% CI
				Cause: All-cause			
30	17	1601	24.9 (13.2 to 36.7)	2344.3 (2234.9 to 2454.9)	2319.4 (2211.3 to 2430.7)	94.04 (63.24 to 172.24)	95.56 (64.27 to 175.11)
90	34	947	75.5 (56.2 to 96.4)	3752.5 (3611.4 to 3898.4)	3676.9 (3532.4 to 3824.9)	49.68 (38.27 to 66.07)	28.75 (21.08 to 41.34)
180	49	620	150.6 (120.4 to 181.9)	4700.8 (4550.5 to 4854.0)	4550.2 (4391.0 to 4702.9)	31.22 (26.07 to 39.30)	13.20 (9.90 to 18.00)
365	104	686	318.5 (275.1 to 361.1)	5802.7 (5621.7 to 5986.7)	5484.2 (5292.6 to 5668.6)	18.22 (16.07 to 21.13)	6.95 (5.81 to 8.77)
730	171	873	620.6 (552.2 to 676.7)	7338.9 (7126.8 to 7535.6)	6718.3 (6509.5 to 6930.0)	11.83 (10.83 to 13.30)	5.44 (4.73 to 6.49)
1095	192	531	1006.5 (918.4 to 1079.7)	8409.7 (8198.2 to 8632.3)	7403.2 (7174.9 to 7638.6)	8.36 (7.76 to 9.13)	2.97 (2.55 to 3.58)
1460	155	441	1366.6 (1268.0 to 1467.3)	9431.7 (9193.0 to 9664.1)	8065.1 (7840.4 to 8322.2)	6.90 (6.44 to 7.42)	3.08 (2.63 to 3.73)
1825	148	368	1767.2 (1653.2 to 1886.3)	10420.8 (10151.8 to 10688.7)	8653.6 (8369.8 to 8923.2)	5.90 (5.53 to 6.33)	2.71 (2.29 to 3.28)
2190	104	267	2102.7 (1965.5 to 2231.9)	11278.8 (11016.8 to 11558.8)	9176.1 (8898.2 to 9453.7)	5.36 (5.05 to 5.72)	2.81 (2.27 to 3.52)
2555	103	232	2509.5 (2351.6 to 2662.8)	12189.8 (11886.6 to 12484.2)	9680.4 (9389.7 to 10003.5)	4.86 (4.57 to 5.18)	2.48 (1.99 to 3.06)
2920	81	159	2908.4 (2708.9 to 3084.9)	12966.0 (12647.7 to 13286.1)	10057.6 (9709.8 to 10372.9)	4.46 (4.20 to 4.77)	2.18 (1.68 to 2.84)
3285	60	135	3293.4 (3094.6 to 3491.2)	13830.6 (13457.5 to 14181.2)	10537.2 (10150.9 to 10886.7)	4.20 (3.93 to 4.47)	2.50 (1.88 to 3.45)
3650	48	98	3709.1 (3475.7 to 3927.6)	14668.5 (14264.6 to 15065.3)	10959.4 (10528.7 to 11340.5)	3.95 (3.71 to 4.22)	2.28 (1.67 to 3.45)
4015	35	64	4166.9 (3896.1 to 4431.3)	15464.9 (15040.4 to 15913.3)	11298.1 (10846.6 to 11752.0)	3.71 (3.47 to 3.97)	2.05 (1.36 to 3.19)
4380	22	36	4681.0 (4337.9 to 5004.2)	16288.3 (15788.8 to 16824.8)	11607.3 (11063.2 to 12207.7)	3.48 (3.24 to 3.78)	1.86 (1.13 to 3.22)
4745	5	16	4989.3 (4541.4 to 5424.5)	17182.2 (16584.1 to 17887.4)	12192.9 (11478.1 to 12986.4)	3.44 (3.17 to 3.80)	3.69 (1.59 to 19.29)
5110	2	4	5220.7 (4684.7 to 5780.1)	17785.3 (16930.5 to 18777.8)	12564.6 (11613.8 to 13639.4)	3.41 (3.05 to 3.84)	***
				Cause: Natural - excluding (HIV/TB and external causes)			
30	17	498	24.9 (13.2 to 36.7)	729.5 (667.2 to 794.8)	704.6 (641.6 to 770.6)	29.26 (19.56 to 52.95)	29.72 (19.86 to 53.81)
90	30	354	69.6 (50.5 to 89.6)	1256.0 (1176.6 to 1334.2)	1186.4 (1101.5 to 1270.5)	18.05 (13.82 to 24.33)	12.18 (8.94 to 18.31)
180	45	264	138.5 (109.8 to 167.7)	1659.9 (1564.2 to 1756.3)	1521.4 (1420.3 to 1617.9)	11.98 (9.95 to 15.08)	6.12 (4.45 to 8.49)

365	92	334	287.1 (243.6 to 327.9)	2196.9 (2084.1 to 2305.1)	1909.7 (1793.2 to 2023.7)	7.65 (6.69 to 9.05)	3.82 (3.11 to 4.94)
730	148	485	548.6 (488.6 to 599.3)	3050.6 (2920.2 to 3184.5)	2502.0 (2368.5 to 2643.9)	5.56 (5.03 to 6.27)	3.49 (2.96 to 4.22)
1095	169	338	888.5 (809.6 to 962.8)	3733.8 (3580.0 to 3881.2)	2845.3 (2680.5 to 3001.7)	4.20 (3.86 to 4.63)	2.15 (1.80 to 2.63)
1460	135	303	1202.3 (1115.3 to 1293.2)	4435.5 (4270.5 to 4584.1)	3233.1 (3060.8 to 3419.7)	3.69 (3.42 to 4.03)	2.43 (2.01 to 3.03)
1825	130	256	1553.5 (1442.9 to 1662.2)	5124.2 (4940.5 to 5301.7)	3570.7 (3374.9 to 3772.2)	3.30 (3.07 to 3.56)	2.15 (1.76 to 2.66)
2190	95	186	1860.2 (1730.8 to 1988.8)	5719.8 (5520.7 to 5928.9)	3859.7 (3656.1 to 4081.1)	3.07 (2.88 to 3.31)	2.15 (1.69 to 2.72)
2555	91	173	2219.8 (2069.5 to 2369.1)	6399.8 (6178.3 to 6627.3)	4180.0 (3932.5 to 4432.4)	2.88 (2.70 to 3.10)	2.09 (1.64 to 2.69)
2920	71	115	2570.4 (2391.1 to 2724.5)	6965.8 (6703.1 to 7214.4)	4395.4 (4120.7 to 4657.6)	2.71 (2.54 to 2.91)	1.80 (1.32 to 2.35)
3285	55	106	2924.5 (2738.3 to 3126.0)	7645.5 (7349.5 to 7920.0)	4721.1 (4399.2 to 5019.7)	2.61 (2.44 to 2.80)	2.14 (1.58 to 2.98)
3650	44	78	3301.6 (3067.9 to 3516.3)	8310.9 (7997.5 to 8632.3)	5009.3 (4670.1 to 5362.7)	2.52 (2.35 to 2.71)	1.98 (1.42 to 2.96)
4015	30	55	3699.0 (3430.8 to 3949.9)	8996.6 (8606.8 to 9336.0)	5297.6 (4898.9 to 5681.2)	2.43 (2.26 to 2.63)	2.06 (1.38 to 3.34)
4380	21	32	4193.0 (3884.3 to 4520.7)	9741.1 (9296.4 to 10181.8)	5548.1 (5034.6 to 6090.6)	2.32 (2.14 to 2.54)	1.73 (1.05 to 3.08)
4745	5	13	4501.3 (4066.4 to 4932.1)	10464.9 (9888.6 to 11067.9)	5963.6 (5287.7 to 6715.9)	2.32 (2.10 to 2.60)	***
5110	2	4	4732.7 (4203.8 to 5284.3)	11068.1 (10260.7 to 12066.8)	6335.4 (5319.8 to 7335.9)	2.34 (2.04 to 2.69)	***
**Cause: Cancer**							
30	3	81	4.4 (0.0 to 9.6)	118.7 (93.8 to 142.2)	114.3 (89.4 to 138.6)	***	***
90	4	99	10.4 (3.0 to 17.8)	266.1 (229.3 to 305.9)	255.7 (217.6 to 296.1)	25.69 (14.76 to 83.91)	***
180	9	86	24.1 (12.1 to 36.3)	397.6 (350.4 to 444.3)	373.5 (325.2 to 421.1)	16.50 (10.69 to 31.63)	9.97 (5.43 to 24.01)
365	18	98	53.2 (37.2 to 72.5)	554.8 (498.2 to 610.6)	501.6 (444.9 to 558.0)	10.43 (7.71 to 14.93)	5.73 (3.77 to 10.57)
730	35	105	114.7 (89.5 to 142.6)	739.2 (674.9 to 810.5)	624.4 (563.4 to 699.7)	6.44 (5.17 to 8.38)	3.20 (2.25 to 4.71)
1095	44	65	203.5 (164.1 to 242.3)	870.1 (801.1 to 946.6)	666.6 (594.5 to 756.0)	4.28 (3.55 to 5.44)	1.59 (1.13 to 2.38)
1460	32	51	277.9 (233.2 to 325.7)	988.0 (911.7 to 1066.4)	710.1 (625.4 to 803.4)	3.55 (3.00 to 4.35)	1.73 (1.18 to 2.81)
1825	32	49	365.2 (309.6 to 419.9)	1119.6 (1033.7 to 1210.1)	754.4 (650.3 to 861.1)	3.07 (2.62 to 3.66)	1.67 (1.06 to 2.65)
2190	18	26	424.4 (369.5 to 482.5)	1202.6 (1110.8 to 1297.7)	778.2 (672.8 to 889.6)	2.83 (2.41 to 3.37)	1.58 (0.89 to 2.91)
2555	12	30	472.0 (407.8 to 540.6)	1319.8 (1219.8 to 1428.4)	847.8 (734.4 to 969.9)	2.80 (2.42 to 3.34)	2.75 (1.51 to 6.28)
2920	18	24	562.8 (491.4 to 639.9)	1434.8 (1321.1 to 1551.2)	872.1 (734.1 to 1018.3)	2.55 (2.18 to 3.01)	1.48 (0.76 to 2.58)
3285	10	16	626.7 (543.6 to 719.9)	1536.2 (1401.2 to 1652.2)	909.5 (760.5 to 1053.3)	2.45 (2.11 to 2.85)	1.78 (0.85 to 4.45)
3650	13	12	739.2 (627.4 to 850.4)	1639.2 (1500.9 to 1769.8)	900.0 (721.5 to 1076.7)	2.22 (1.88 to 2.65)	1.03 (0.46 to 2.45)
4015	12	4	895.1 (754.9 to 1027.5)	1689.7 (1545.1 to 1840.3)	794.7 (602.3 to 995.2)	1.89 (1.61 to 2.25)	***
4380	7	5	1054.3 (862.1 to 1238.4)	1788.7 (1608.8 to 1970.5)	734.4 (486.6 to 1008.1)	1.70 (1.40 to 2.13)	***
4745	1	2	1122.5 (894.1 to 1371.2)	1900.8 (1701.0 to 2146.4)	778.3 (455.6 to 1098.5)	1.69 (1.33 to 2.20)	***
5110	0	1	1122.5 (894.1 to 1371.2)	2033.3 (1728.8 to 2450.8)	910.8 (534.3 to 1363.6)	1.81 (1.42 to 2.42)	***
**Cause: Cardiovascular**							
30	4	64	5.9 (1.5 to 11.7)	93.7 (72.5 to 117.2)	87.9 (66.0 to 112.1)	***	***
90	10	52	20.7 (11.9 to 32.6)	171.1 (143.1 to 201.4)	150.4 (116.6 to 180.7)	8.25 (4.96 to 15.59)	5.37 (2.87 to 11.92)

180	17	39	46.7 (33.2 to 64.8)	230.8 (197.0 to 268.1)	184.1 (144.1 to 225.0)	4.94 (3.44 to 7.17)	2.39 (1.39 to 4.55)
365	28	55	92.2 (70.6 to 114.5)	319.2 (279.5 to 363.5)	227.0 (182.2 to 276.4)	3.46 (2.64 to 4.60)	2.07 (1.34 to 3.27)
730	46	90	173.3 (142.4 to 205.7)	477.3 (429.4 to 530.7)	304.0 (248.3 to 366.8)	2.75 (2.29 to 3.39)	2.08 (1.46 to 2.95)
1095	54	72	282.5 (242.7 to 323.4)	623.7 (562.7 to 692.5)	341.2 (270.3 to 414.6)	2.21 (1.87 to 2.65)	1.43 (1.02 to 2.06)
1460	42	77	380.9 (332.1 to 431.1)	803.1 (728.0 to 884.1)	422.2 (341.0 to 511.2)	2.11 (1.82 to 2.47)	1.99 (1.40 to 3.09)
1825	46	60	504.2 (440.9 to 567.0)	965.2 (874.5 to 1054.0)	461.0 (361.4 to 563.6)	1.91 (1.66 to 2.23)	1.42 (0.95 to 2.06)
2190	33	47	611.1 (536.1 to 690.3)	1115.2 (1018.5 to 1216.8)	504.0 (388.3 to 622.4)	1.82 (1.58 to 2.13)	1.56 (0.96 to 2.43)
2555	35	36	751.5 (671.1 to 842.1)	1255.8 (1153.5 to 1364.2)	504.4 (368.2 to 653.8)	1.67 (1.45 to 1.92)	1.13 (0.72 to 1.78)
2920	16	28	831.5 (746.5 to 926.9)	1395.1 (1280.1 to 1511.5)	563.6 (412.1 to 714.1)	1.68 (1.45 to 1.92)	1.94 (1.11 to 3.67)
3285	20	18	961.3 (869.6 to 1086.4)	1513.4 (1384.8 to 1648.9)	552.0 (374.2 to 706.3)	1.57 (1.36 to 1.77)	1.00 (0.54 to 1.92)
3650	17	22	1108.8 (985.4 to 1258.5)	1703.0 (1557.4 to 1853.2)	594.2 (391.2 to 773.9)	1.54 (1.32 to 1.76)	1.45 (0.82 to 2.92)
4015	5	16	1171.2 (1041.7 to 1324.9)	1899.9 (1722.1 to 2080.0)	728.7 (504.9 to 945.8)	1.62 (1.38 to 1.87)	3.59 (1.60 to 15.97)
4380	5	11	1288.4 (1120.6 to 1478.1)	2174.2 (1946.9 to 2439.8)	885.8 (583.7 to 1205.0)	1.69 (1.40 to 2.00)	***
4745	1	2	1324.1 (1145.8 to 1516.0)	2286.0 (2031.6 to 2631.3)	961.9 (630.9 to 1381.1)	1.73 (1.42 to 2.14)	***
5110	1	1	1457.0 (1178.6 to 1830.5)	2478.1 (2061.4 to 3000.3)	1021.0 (433.6 to 1661.0)	1.70 (1.25 to 2.29)	***
**Cause: Endocrine**							
30	1	21	1.5 (0.0 to 4.4)	30.8 (17.6 to 44.0)	29.3 (16.1 to 43.9)	***	***
90	3	22	5.9 (1.5 to 11.9)	63.5 (44.4 to 82.6)	57.5 (38.4 to 76.9)	***	***
180	3	11	10.5 (3.0 to 19.3)	80.3 (59.5 to 101.4)	69.8 (47.4 to 92.2)	7.67 (3.97 to 24.96)	***
365	6	26	20.2 (10.7 to 32.5)	122.1 (96.2 to 148.1)	101.9 (72.8 to 129.7)	6.04 (3.59 to 12.26)	4.56 (2.30 to 14.78)
730	19	41	54.0 (35.0 to 74.6)	194.1 (160.8 to 230.0)	140.1 (99.9 to 181.6)	3.59 (2.45 to 5.56)	2.30 (1.44 to 4.27)
1095	9	31	72.2 (51.0 to 94.8)	256.4 (216.0 to 297.2)	184.2 (137.3 to 231.7)	3.55 (2.58 to 5.08)	3.70 (1.92 to 8.59)
1460	14	25	104.8 (78.5 to 131.7)	314.3 (269.5 to 363.4)	209.5 (157.6 to 262.8)	3.00 (2.24 to 4.12)	1.93 (1.00 to 4.09)
1825	12	20	137.1 (103.9 to 172.0)	369.0 (316.3 to 422.7)	231.9 (170.4 to 296.4)	2.69 (2.02 to 3.61)	1.82 (0.87 to 4.05)
2190	10	23	169.3 (131.9 to 208.3)	442.3 (380.6 to 499.9)	273.0 (202.3 to 344.6)	2.61 (2.04 to 3.45)	2.52 (1.21 to 6.38)
2555	9	14	204.5 (162.6 to 251.7)	497.4 (431.8 to 563.7)	292.9 (213.9 to 373.9)	2.43 (1.89 to 3.18)	1.71 (0.74 to 4.40)
2920	5	12	228.6 (181.6 to 280.5)	557.1 (483.3 to 629.9)	328.5 (240.2 to 416.3)	2.44 (1.92 to 3.13)	***
3285	2	19	240.6 (191.3 to 295.2)	680.6 (588.6 to 769.8)	440.0 (332.6 to 549.8)	2.83 (2.21 to 3.69)	***
3650	3	9	265.2 (207.7 to 326.6)	759.7 (661.0 to 867.0)	494.5 (380.1 to 613.7)	2.86 (2.24 to 3.78)	***
4015	1	10	277.4 (213.4 to 345.5)	885.5 (765.3 to 1020.9)	608.1 (476.1 to 761.9)	3.19 (2.51 to 4.30)	***
4380	0	3	277.4 (213.4 to 345.5)	958.4 (810.8 to 1124.3)	681.0 (512.3 to 857.6)	3.45 (2.65 to 4.65)	***
4745	0	3	277.4 (213.4 to 345.5)	1095.4 (898.5 to 1317.1)	818.0 (610.7 to 1050.9)	3.95 (2.97 to 5.34)	***
5110	1	0	375.9 (228.7 to 617.0)	1095.4 (898.5 to 1317.1)	719.6 (426.7 to 983.5)	2.91 (1.73 to 4.92)	***
**Cause: Respiratory**							
30	4	135	5.9 (1.5 to 13.2)	197.7 (166.2 to 231.4)	191.9 (159.7 to 225.5)	***	***

90	1	54	7.3 (1.5 to 14.7)	278.0 (241.4 to 317.7)	270.7 (233.1 to 310.3)	37.84 (18.73 to 189.39)	***
180	7	51	18.1 (9.0 to 30.1)	356.2 (311.9 to 399.6)	338.1 (293.9 to 382.5)	19.63 (11.92 to 42.99)	7.60 (3.71 to 25.27)
365	14	61	40.6 (26.1 to 56.4)	454.6 (404.3 to 503.1)	414.0 (363.7 to 465.4)	11.20 (8.07 to 17.88)	4.59 (2.86 to 9.04)
730	9	103	56.7 (38.5 to 76.5)	635.6 (573.2 to 697.0)	578.9 (515.7 to 640.6)	11.21 (8.22 to 16.50)	12.20 (6.82 to 27.83)
1095	24	61	104.8 (77.5 to 133.4)	758.4 (692.4 to 827.0)	653.7 (582.0 to 725.9)	7.24 (5.52 to 9.94)	2.73 (1.77 to 4.65)
1460	18	58	146.7 (113.5 to 182.5)	891.6 (819.6 to 969.0)	744.9 (662.6 to 828.0)	6.08 (4.78 to 7.85)	3.49 (2.14 to 7.09)
1825	16	59	190.2 (151.7 to 230.7)	1051.3 (964.7 to 1142.8)	861.1 (762.9 to 961.5)	5.53 (4.43 to 6.99)	4.02 (2.26 to 7.72)
2190	15	34	238.0 (191.0 to 285.9)	1159.0 (1068.5 to 1254.0)	921.0 (813.0 to 1026.9)	4.87 (3.98 to 6.19)	2.48 (1.46 to 4.88)
2555	12	34	285.3 (228.0 to 338.1)	1294.8 (1197.8 to 1393.2)	1009.5 (892.6 to 1133.3)	4.54 (3.73 to 5.72)	3.12 (1.80 to 6.91)
2920	8	20	324.2 (262.7 to 383.4)	1393.8 (1289.2 to 1505.6)	1069.6 (936.0 to 1197.8)	4.30 (3.53 to 5.35)	2.77 (1.22 to 7.05)
3285	10	25	390.0 (316.2 to 463.4)	1549.7 (1430.1 to 1683.7)	1159.7 (1010.7 to 1307.9)	3.97 (3.25 to 5.02)	2.78 (1.45 to 7.92)
3650	2	17	405.5 (331.3 to 481.5)	1686.7 (1558.8 to 1819.7)	1281.2 (1125.3 to 1435.7)	4.16 (3.45 to 5.22)	***
4015	3	8	442.8 (349.2 to 532.5)	1788.2 (1633.4 to 1945.2)	1345.5 (1169.4 to 1517.8)	4.04 (3.28 to 5.24)	***
4380	5	3	564.0 (434.3 to 713.6)	1873.0 (1713.1 to 2057.3)	1309.0 (1059.8 to 1525.8)	3.32 (2.57 to 4.39)	***
4745	3	2	768.3 (528.7 to 1087.8)	2018.4 (1783.0 to 2312.4)	1250.1 (850.7 to 1618.5)	2.63 (1.84 to 3.90)	***
5110	0	1	768.3 (528.7 to 1087.8)	2148.2 (1793.6 to 2531.5)	1379.9 (886.2 to 1862.9)	2.80 (1.90 to 4.20)	***
				**Cause: External**			
30	0	7	0.0 (0.0 to 0.0)	10.3 (2.9 to 19.1)	10.3 (2.9 to 19.1)	***	***
90	3	11	4.5 (0.0 to 10.4)	26.6 (14.8 to 41.4)	22.2 (8.9 to 36.9)	***	***
180	3	19	9.1 (3.0 to 18.0)	55.8 (37.7 to 75.0)	46.7 (26.4 to 67.9)	6.15 (2.91 to 23.12)	***
365	10	23	25.1 (14.2 to 37.9)	92.5 (69.8 to 118.5)	67.4 (40.3 to 94.5)	3.68 (2.18 to 6.62)	2.42 (1.22 to 6.31)
730	17	31	55.2 (37.9 to 74.9)	146.7 (119.3 to 180.5)	91.5 (57.9 to 128.2)	2.66 (1.85 to 4.11)	1.94 (1.07 to 3.85)
1095	19	18	93.4 (68.6 to 118.3)	182.7 (150.6 to 219.2)	89.3 (47.6 to 133.5)	1.96 (1.41 to 2.82)	1.02 (0.51 to 2.00)
1460	15	20	128.3 (96.5 to 159.7)	230.0 (191.3 to 274.4)	101.7 (49.9 to 157.8)	1.79 (1.33 to 2.52)	1.44 (0.78 to 3.09)
1825	13	19	164.2 (128.8 to 199.6)	280.8 (233.6 to 331.1)	116.6 (62.6 to 176.9)	1.71 (1.34 to 2.29)	1.59 (0.73 to 3.27)
2190	8	14	189.9 (149.5 to 229.6)	325.9 (274.9 to 383.3)	136.0 (72.8 to 201.6)	1.72 (1.34 to 2.25)	1.92 (0.88 to 5.81)
2555	8	15	221.0 (176.7 to 265.1)	385.4 (325.4 to 453.5)	164.3 (90.9 to 241.4)	1.74 (1.35 to 2.29)	2.07 (0.85 to 6.07)
2920	9	5	265.0 (211.1 to 315.3)	409.0 (345.2 to 474.2)	144.0 (63.7 to 236.3)	1.54 (1.21 to 2.05)	***
3285	3	7	282.8 (223.9 to 342.9)	452.9 (387.4 to 526.7)	170.1 (80.4 to 273.0)	1.60 (1.25 to 2.16)	***
3650	2	4	300.7 (233.4 to 370.6)	488.3 (410.0 to 571.8)	187.6 (88.1 to 295.6)	1.62 (1.24 to 2.21)	***
4015	4	2	349.2 (269.2 to 434.1)	515.3 (432.1 to 602.8)	166.0 (45.4 to 290.6)	1.48 (1.11 to 1.98)	***
4380	0	2	349.2 (269.2 to 434.1)	554.6 (450.7 to 663.8)	205.3 (64.7 to 336.7)	1.59 (1.15 to 2.14)	***
4745	0	1	349.2 (269.2 to 434.1)	591.6 (478.3 to 728.6)	242.3 (94.3 to 390.7)	1.69 (1.23 to 2.37)	***
5110	0	0	349.2 (269.2 to 434.1)	591.6 (478.3 to 728.6)	242.3 (94.3 to 390.7)	1.69 (1.23 to 2.37)	***

*** IRR/RR was not estimated when there were fewer than 20 events in the risk period or fewer than 5 events in one group. Confidence intervals obtained through percentile bootstrap.

Supplementary Table 13. Estimated risk of death by cause-specific (defined by ICD-10 chapters) comparing diagnosed Tuberculosis cases to non-exposed controls in Males.

	Number of events		Risk per 100,000				
Time - Days	Unexposed	Exposed	Unexposed	Exposed	Risk difference per 100,000 - 95% CI	Risk ratio - 95% CI	Incidence rate ratio - 95% CI
				Cause: All-cause			
30	70	3465	59.9 (47.5 to 74.0)	2959.2 (2870.4 to 3053.1)	2899.3 (2807.6 to 2994.0)	49.38 (39.89 to 62.47)	50.42 (40.70 to 63.80)
90	131	1922	174.2 (151.1 to 199.7)	4629.9 (4510.0 to 4744.0)	4455.7 (4336.6 to 4573.3)	26.58 (23.34 to 30.54)	15.25 (12.89 to 18.75)
180	161	1319	319.2 (288.4 to 351.3)	5814.8 (5690.9 to 5944.2)	5495.6 (5372.8 to 5625.8)	18.22 (16.51 to 20.15)	8.63 (7.46 to 10.25)
365	360	1607	662.3 (618.2 to 710.6)	7342.8 (7216.7 to 7486.6)	6680.5 (6541.6 to 6838.6)	11.09 (10.27 to 11.94)	4.76 (4.26 to 5.37)
730	597	2115	1299.6 (1231.4 to 1371.1)	9592.7 (9429.2 to 9763.4)	8293.1 (8115.6 to 8481.6)	7.38 (6.96 to 7.83)	3.83 (3.53 to 4.24)
1095	545	1484	1977.6 (1885.5 to 2066.7)	11437.7 (11252.0 to 11623.7)	9460.1 (9257.3 to 9672.4)	5.78 (5.52 to 6.07)	2.99 (2.72 to 3.34)
1460	517	1211	2734.8 (2627.9 to 2836.5)	13202.3 (13003.9 to 13407.7)	10467.6 (10248.0 to 10709.2)	4.83 (4.63 to 5.04)	2.60 (2.36 to 2.89)
1825	416	1001	3450.3 (3326.0 to 3576.2)	14919.8 (14688.7 to 15150.3)	11469.5 (11217.2 to 11744.7)	4.32 (4.16 to 4.50)	2.70 (2.43 to 3.05)
2190	321	809	4118.1 (3972.8 to 4265.8)	16590.3 (16349.4 to 16834.1)	12472.2 (12164.5 to 12769.5)	4.03 (3.88 to 4.19)	2.86 (2.52 to 3.29)
2555	254	619	4768.7 (4594.4 to 4936.4)	18161.5 (17888.3 to 18430.4)	13392.8 (13089.1 to 13690.4)	3.81 (3.67 to 3.96)	2.80 (2.44 to 3.32)
2920	200	431	5420.5 (5215.9 to 5607.8)	19552.2 (19237.5 to 19835.4)	14131.6 (13792.1 to 14496.5)	3.61 (3.48 to 3.75)	2.50 (2.11 to 3.00)
3285	161	377	6116.4 (5896.7 to 6318.0)	21153.4 (20805.5 to 21480.8)	15037.1 (14622.9 to 15457.2)	3.46 (3.34 to 3.60)	2.75 (2.33 to 3.35)
3650	125	207	6853.4 (6605.6 to 7101.4)	22383.4 (22036.4 to 22743.2)	15529.9 (15075.9 to 15955.8)	3.27 (3.15 to 3.40)	1.97 (1.58 to 2.47)
4015	72	148	7507.5 (7211.2 to 7789.0)	23761.6 (23333.0 to 24182.9)	16254.0 (15749.5 to 16731.7)	3.17 (3.04 to 3.30)	2.47 (1.86 to 3.29)
4380	39	82	8201.1 (7845.5 to 8556.5)	25210.7 (24690.7 to 25760.2)	17009.6 (16420.4 to 17585.0)	3.07 (2.93 to 3.22)	2.55 (1.76 to 3.76)

4745	21	24	9270.8 (8663.6 to 9897.0)	26392.1 (25661.6 to 27190.3)	17121.4 (16172.6 to 18104.5)	2.85 (2.65 to 3.07)	1.41 (0.74 to 2.75)
5110	5	1	10301.5 (9235.8 to 11505.5)	26722.2 (25866.3 to 27730.2)	16420.7 (14811.9 to 17975.8)	2.59 (2.29 to 2.90)	***
**Cause: Natural - excluding (HIV/TB and external causes)**							
30	37	1026	31.7 (22.3 to 41.1)	876.6 (827.0 to 929.0)	844.9 (797.0 to 894.4)	27.68 (21.21 to 39.12)	28.24 (21.63 to 39.89)
90	86	765	106.7 (88.4 to 127.6)	1541.6 (1474.6 to 1619.5)	1434.9 (1368.0 to 1505.2)	14.45 (12.11 to 17.60)	9.25 (7.43 to 12.08)
180	121	578	215.8 (190.2 to 243.1)	2060.9 (1990.2 to 2146.3)	1845.1 (1766.4 to 1929.8)	9.55 (8.51 to 10.96)	5.03 (4.18 to 6.15)
365	245	817	449.3 (410.0 to 490.8)	2838.3 (2747.3 to 2941.2)	2389.0 (2286.1 to 2495.9)	6.32 (5.72 to 6.94)	3.55 (3.10 to 4.13)
730	417	1207	895.2 (842.2 to 955.3)	4125.8 (4014.5 to 4245.0)	3230.6 (3103.9 to 3363.2)	4.61 (4.28 to 4.93)	3.13 (2.84 to 3.54)
1095	389	899	1379.4 (1304.9 to 1460.5)	5244.0 (5109.1 to 5375.9)	3864.6 (3720.5 to 4012.5)	3.80 (3.56 to 4.04)	2.54 (2.27 to 2.86)
1460	380	747	1937.1 (1843.5 to 2027.3)	6332.9 (6180.2 to 6478.9)	4395.7 (4227.4 to 4583.7)	3.27 (3.11 to 3.46)	2.18 (1.94 to 2.49)
1825	305	647	2463.5 (2348.0 to 2579.0)	7444.7 (7276.0 to 7619.5)	4981.1 (4781.3 to 5196.4)	3.02 (2.88 to 3.18)	2.38 (2.11 to 2.75)
2190	253	545	2989.1 (2860.6 to 3104.4)	8568.3 (8364.9 to 8756.5)	5579.1 (5370.1 to 5807.5)	2.87 (2.74 to 3.00)	2.45 (2.14 to 2.83)
2555	179	414	3447.0 (3303.6 to 3587.6)	9619.8 (9402.8 to 9830.5)	6172.8 (5928.8 to 6419.7)	2.79 (2.67 to 2.92)	2.66 (2.26 to 3.17)
2920	142	290	3908.8 (3746.6 to 4075.2)	10554.2 (10309.4 to 10789.9)	6645.4 (6391.6 to 6948.8)	2.70 (2.58 to 2.83)	2.37 (1.95 to 2.93)
3285	124	266	4441.6 (4244.3 to 4635.8)	11688.8 (11417.4 to 11942.2)	7247.2 (6946.8 to 7562.5)	2.63 (2.52 to 2.77)	2.52 (2.04 to 3.15)
3650	92	156	4982.4 (4757.2 to 5217.3)	12625.1 (12324.6 to 12937.5)	7642.7 (7297.8 to 7995.9)	2.53 (2.41 to 2.66)	2.01 (1.56 to 2.59)
4015	57	117	5489.1 (5229.9 to 5745.5)	13714.3 (13351.2 to 14075.5)	8225.2 (7802.2 to 8672.6)	2.50 (2.38 to 2.63)	2.46 (1.83 to 3.38)
4380	32	66	6072.1 (5755.0 to 6397.7)	14869.4 (14409.0 to 15325.0)	8797.3 (8292.1 to 9290.4)	2.45 (2.31 to 2.59)	2.50 (1.74 to 4.06)
4745	16	17	6924.8 (6389.3 to 7455.6)	15662.3 (15045.5 to 16296.5)	8737.5 (7928.7 to 9550.7)	2.26 (2.09 to 2.47)	1.31 (0.62 to 2.99)
5110	5	0	7955.6 (6895.1 to 9105.7)	15662.3 (15045.5 to 16296.5)	7706.7 (6254.4 to 8989.2)	1.97 (1.69 to 2.28)	***
**Cause: Cancer**							
30	6	164	5.1 (1.7 to 9.4)	140.2 (117.5 to 160.7)	135.1 (112.9 to 155.6)	27.27 (14.30 to 92.35)	27.84 (14.60 to 94.26)
90	15	199	18.2 (10.4 to 26.1)	313.3 (278.7 to 347.3)	295.0 (261.3 to 329.3)	17.17 (11.77 to 29.52)	13.79 (8.59 to 26.41)
180	15	164	31.8 (22.5 to 43.4)	460.6 (421.6 to 501.0)	428.7 (389.3 to 469.6)	14.47 (10.59 to 21.15)	11.51 (7.44 to 21.06)
365	41	240	71.2 (55.9 to 89.2)	688.8 (641.8 to 736.5)	617.7 (566.5 to 667.3)	9.68 (7.55 to 12.29)	6.24 (4.36 to 8.80)
730	77	273	153.1 (128.0 to 179.4)	979.5 (918.1 to 1039.2)	826.4 (757.2 to 891.9)	6.40 (5.40 to 7.62)	3.84 (3.07 to 5.07)
1095	51	168	217.0 (187.6 to 248.5)	1188.8 (1119.2 to 1253.8)	971.7 (891.8 to 1047.0)	5.48 (4.72 to 6.40)	3.62 (2.64 to 5.08)
1460	72	113	322.9 (287.2 to 361.6)	1353.6 (1273.4 to 1425.1)	1030.8 (940.3 to 1116.7)	4.19 (3.68 to 4.79)	1.74 (1.32 to 2.44)

1825	51	120	410.7 (366.7 to 454.3)	1559.8 (1470.7 to 1641.7)	1149.1 (1043.2 to 1248.9)	3.80 (3.38 to 4.29)	2.64 (1.91 to 3.77)
2190	44	94	502.7 (454.5 to 556.5)	1752.6 (1651.5 to 1837.6)	1249.9 (1143.2 to 1352.8)	3.49 (3.14 to 3.92)	2.43 (1.73 to 3.45)
2555	33	67	585.7 (522.5 to 645.2)	1920.7 (1816.8 to 2011.6)	1335.0 (1225.1 to 1449.3)	3.28 (2.94 to 3.69)	2.34 (1.58 to 3.62)
2920	25	49	668.4 (604.2 to 743.6)	2078.6 (1956.7 to 2176.5)	1410.2 (1288.0 to 1529.0)	3.11 (2.76 to 3.48)	2.28 (1.41 to 3.93)
3285	26	47	778.9 (705.7 to 864.1)	2280.2 (2146.0 to 2392.8)	1501.3 (1338.3 to 1633.8)	2.93 (2.60 to 3.28)	2.12 (1.24 to 3.36)
3650	18	34	884.1 (798.0 to 976.5)	2486.1 (2328.5 to 2611.3)	1602.0 (1419.2 to 1759.7)	2.81 (2.51 to 3.16)	2.24 (1.36 to 4.23)
4015	12	20	994.5 (882.7 to 1112.6)	2672.5 (2501.0 to 2823.7)	1678.0 (1468.1 to 1871.3)	2.69 (2.37 to 3.02)	2.00 (1.00 to 4.80)
4380	6	17	1099.3 (977.9 to 1249.4)	2977.1 (2765.1 to 3182.8)	1877.8 (1623.7 to 2127.1)	2.71 (2.35 to 3.11)	3.44 (1.49 to 10.08)
4745	5	3	1352.4 (1110.1 to 1698.4)	3101.8 (2858.0 to 3352.3)	1749.4 (1328.1 to 2136.0)	2.29 (1.79 to 2.87)	***
5110	1	0	1469.0 (1149.6 to 1896.6)	3101.8 (2858.0 to 3352.3)	1632.8 (1118.3 to 2054.1)	2.11 (1.60 to 2.72)	***
**Cause: Cardiovascular**							
30	9	125	7.7 (3.4 to 12.8)	106.8 (89.7 to 126.9)	99.1 (80.7 to 119.6)	13.89 (8.08 to 29.67)	14.15 (8.22 to 30.20)
90	28	106	32.1 (21.7 to 42.6)	199.0 (174.3 to 226.2)	166.9 (139.9 to 195.4)	6.20 (4.52 to 9.09)	3.94 (2.66 to 6.39)
180	36	107	64.6 (50.5 to 80.9)	295.2 (266.3 to 329.4)	230.6 (195.9 to 266.5)	4.57 (3.62 to 5.97)	3.13 (2.20 to 4.59)
365	93	147	153.0 (132.5 to 176.5)	434.7 (398.1 to 476.0)	281.7 (236.2 to 327.2)	2.84 (2.40 to 3.36)	1.68 (1.30 to 2.15)
730	129	225	291.1 (257.3 to 324.0)	674.4 (628.8 to 725.1)	383.3 (330.2 to 447.5)	2.32 (2.04 to 2.66)	1.89 (1.56 to 2.37)
1095	151	191	478.7 (435.0 to 519.6)	912.2 (849.7 to 975.8)	433.5 (355.3 to 507.3)	1.91 (1.70 to 2.14)	1.39 (1.11 to 1.70)
1460	124	182	659.7 (607.4 to 714.2)	1177.9 (1108.7 to 1256.3)	518.2 (429.0 to 610.8)	1.79 (1.61 to 1.99)	1.63 (1.29 to 2.14)
1825	102	134	835.4 (767.5 to 904.5)	1409.7 (1330.8 to 1499.1)	574.2 (464.9 to 692.4)	1.69 (1.52 to 1.87)	1.48 (1.13 to 1.93)
2190	89	113	1019.7 (944.6 to 1094.5)	1643.7 (1549.2 to 1739.4)	623.9 (505.3 to 748.5)	1.61 (1.47 to 1.79)	1.44 (1.08 to 1.93)
2555	65	86	1186.8 (1101.1 to 1272.7)	1865.3 (1757.6 to 1970.2)	678.5 (538.2 to 823.2)	1.57 (1.44 to 1.74)	1.52 (1.12 to 2.13)
2920	56	70	1366.8 (1268.7 to 1470.1)	2090.4 (1964.4 to 2212.7)	723.6 (554.6 to 876.4)	1.53 (1.39 to 1.68)	1.45 (0.98 to 2.05)
3285	41	54	1546.2 (1429.1 to 1669.1)	2324.7 (2181.9 to 2457.3)	778.6 (599.7 to 950.2)	1.50 (1.37 to 1.65)	1.55 (1.03 to 2.31)
3650	40	35	1780.8 (1647.7 to 1926.7)	2534.4 (2383.1 to 2678.8)	753.5 (530.7 to 932.7)	1.42 (1.29 to 1.55)	1.04 (0.66 to 1.58)
4015	19	19	1941.7 (1789.5 to 2102.6)	2707.3 (2548.3 to 2856.3)	765.6 (539.7 to 975.8)	1.39 (1.26 to 1.53)	1.20 (0.66 to 2.29)
4380	15	17	2195.7 (1990.0 to 2394.3)	2995.2 (2784.4 to 3204.6)	799.5 (511.9 to 1068.0)	1.36 (1.22 to 1.52)	1.38 (0.72 to 2.73)
4745	6	3	2572.5 (2228.7 to 2970.2)	3105.7 (2865.0 to 3363.8)	533.2 (2.9 to 914.8)	1.21 (1.00 to 1.40)	***
5110	0	0	2572.5 (2228.7 to 2970.2)	3105.7 (2865.0 to 3363.8)	533.2 (2.9 to 914.8)	1.21 (1.00 to 1.40)	***
**Cause: Endocrine**							
30	2	64	1.7 (0.0 to 4.3)	54.7 (41.0 to 68.3)	53.0 (39.3 to 66.7)	***	***
90	8	42	8.7 (3.5 to 14.8)	91.3 (74.0 to 107.6)	82.6 (64.3 to 99.7)	10.48 (5.89 to 24.42)	5.46 (2.81 to 15.59)
180	9	35	16.8 (9.7 to 24.8)	122.7 (102.5 to 142.6)	105.9 (84.0 to 126.2)	7.30 (4.75 to 13.06)	4.10 (2.11 to 11.24)
365	21	42	36.8 (26.2 to 48.2)	162.8 (138.6 to 186.7)	126.0 (97.7 to 151.6)	4.42 (3.19 to 6.39)	2.13 (1.26 to 3.93)
730	37	62	76.2 (59.7 to 94.2)	229.7 (200.1 to 258.4)	153.5 (117.6 to 186.0)	3.01 (2.33 to 3.93)	1.81 (1.22 to 2.82)
1095	26	59	108.6 (88.2 to 129.2)	302.5 (266.1 to 335.2)	193.9 (153.2 to 231.6)	2.79 (2.23 to 3.51)	2.49 (1.63 to 4.15)

1460	27	55	148.7 (123.6 to 175.0)	382.6 (343.4 to 424.3)	233.9 (187.7 to 281.5)	2.57 (2.11 to 3.21)	2.26 (1.43 to 3.75)
1825	17	40	178.1 (149.1 to 206.6)	451.0 (410.7 to 495.6)	272.9 (219.9 to 329.2)	2.53 (2.12 to 3.11)	2.64 (1.60 to 5.01)
2190	16	43	211.1 (178.8 to 241.8)	539.8 (487.3 to 593.8)	328.7 (269.2 to 394.4)	2.56 (2.15 to 3.10)	3.05 (1.81 to 6.02)
2555	12	31	242.1 (205.0 to 276.3)	617.5 (562.8 to 678.8)	375.4 (306.8 to 449.9)	2.55 (2.15 to 3.13)	2.97 (1.58 to 6.57)
2920	4	18	254.8 (215.7 to 292.7)	674.2 (611.9 to 744.1)	419.4 (348.1 to 501.0)	2.65 (2.23 to 3.19)	5.23 (2.15 to 26.80)
3285	7	17	285.7 (243.0 to 334.4)	747.4 (679.6 to 819.8)	461.7 (380.4 to 550.4)	2.62 (2.17 to 3.17)	2.85 (1.29 to 8.76)
3650	5	12	315.9 (265.4 to 367.0)	820.2 (743.4 to 902.9)	504.3 (406.3 to 605.1)	2.60 (2.16 to 3.19)	***
4015	2	5	334.1 (278.2 to 390.4)	869.7 (781.7 to 968.0)	535.5 (436.2 to 652.2)	2.60 (2.14 to 3.23)	***
4380	4	2	417.1 (321.6 to 527.4)	903.4 (805.0 to 1011.8)	486.3 (342.8 to 631.4)	2.17 (1.69 to 2.88)	***
4745	1	1	451.3 (345.3 to 583.1)	946.6 (814.4 to 1097.2)	495.4 (326.6 to 681.6)	2.10 (1.60 to 2.88)	***
5110	0	0	451.3 (345.3 to 583.1)	946.6 (814.4 to 1097.2)	495.4 (326.6 to 681.6)	2.10 (1.60 to 2.88)	***
**Cause: Respiratory**							
30	6	309	5.1 (1.7 to 9.0)	263.8 (234.3 to 291.6)	258.7 (228.8 to 288.6)	51.36 (29.30 to 163.19)	52.45 (29.94 to 166.84)
90	8	133	12.1 (6.0 to 19.0)	379.4 (342.4 to 414.8)	367.3 (330.4 to 402.9)	31.42 (20.34 to 65.47)	17.28 (10.14 to 49.76)
180	16	98	26.5 (16.9 to 36.2)	467.4 (428.2 to 507.1)	440.9 (401.1 to 483.6)	17.61 (13.06 to 28.42)	6.45 (4.21 to 12.33)
365	20	158	45.7 (32.6 to 58.2)	617.8 (574.5 to 664.8)	572.1 (526.9 to 621.7)	13.52 (10.50 to 19.38)	8.42 (5.52 to 15.12)
730	50	250	99.3 (79.8 to 118.1)	884.7 (823.8 to 937.0)	785.4 (721.6 to 842.7)	8.91 (7.35 to 11.13)	5.41 (4.08 to 7.63)
1095	51	168	162.5 (136.8 to 189.4)	1093.3 (1027.9 to 1154.0)	930.8 (862.0 to 995.1)	6.73 (5.65 to 8.02)	3.62 (2.72 to 5.07)
1460	40	157	221.6 (190.0 to 252.2)	1321.5 (1251.0 to 1387.1)	1100.0 (1021.8 to 1167.9)	5.96 (5.18 to 7.00)	4.36 (3.12 to 6.08)
1825	36	135	283.7 (245.8 to 318.2)	1552.1 (1473.7 to 1625.5)	1268.4 (1179.5 to 1353.3)	5.47 (4.79 to 6.36)	4.21 (3.02 to 6.44)
2190	29	96	344.6 (301.9 to 384.2)	1749.8 (1658.6 to 1838.3)	1405.3 (1304.4 to 1510.8)	5.08 (4.50 to 5.88)	3.76 (2.49 to 5.79)
2555	18	78	390.3 (337.3 to 439.4)	1946.9 (1851.6 to 2047.2)	1556.6 (1455.7 to 1670.2)	4.99 (4.42 to 5.79)	4.99 (3.26 to 9.12)
2920	17	59	446.2 (388.4 to 498.1)	2138.4 (2032.4 to 2253.2)	1692.3 (1574.3 to 1813.5)	4.79 (4.21 to 5.53)	4.03 (2.33 to 7.42)
3285	9	49	484.8 (424.0 to 543.5)	2344.6 (2227.3 to 2459.9)	1859.8 (1723.9 to 1994.5)	4.84 (4.26 to 5.58)	6.40 (3.57 to 18.50)
3650	10	26	544.5 (475.5 to 616.7)	2498.2 (2370.8 to 2625.8)	1953.7 (1814.0 to 2095.8)	4.59 (4.06 to 5.28)	3.09 (1.43 to 7.40)
4015	5	35	593.9 (515.6 to 679.0)	2819.7 (2646.2 to 3008.0)	2225.8 (2044.1 to 2420.6)	4.75 (4.12 to 5.58)	8.40 (3.73 to 42.68)
4380	1	13	610.1 (523.4 to 705.3)	3029.5 (2822.1 to 3270.8)	2419.4 (2205.1 to 2659.0)	4.97 (4.24 to 5.88)	***
4745	1	4	681.7 (550.5 to 859.0)	3253.2 (2945.6 to 3556.8)	2571.5 (2206.2 to 2934.6)	4.77 (3.71 to 6.14)	***
5110	1	0	838.8 (574.9 to 1266.2)	3253.2 (2945.6 to 3556.8)	2414.4 (1952.7 to 2848.4)	3.88 (2.57 to 5.68)	***
**Cause: External**							
30	31	52	26.5 (17.5 to 36.4)	44.4 (33.3 to 56.9)	17.9 (3.3 to 32.0)	1.67 (1.10 to 2.63)	1.71 (1.12 to 2.68)
90	42	61	63.2 (49.4 to 77.8)	97.5 (80.7 to 115.7)	34.4 (13.5 to 56.0)	1.54 (1.18 to 2.03)	1.51 (1.04 to 2.28)
180	40	132	99.1 (81.5 to 117.2)	216.3 (192.3 to 245.9)	117.2 (87.2 to 150.5)	2.18 (1.76 to 2.73)	3.48 (2.45 to 5.21)
365	107	263	201.1 (176.5 to 228.4)	467.3 (427.5 to 508.9)	266.2 (216.1 to 311.6)	2.32 (1.98 to 2.70)	2.62 (2.13 to 3.31)

730	174	450	386.1 (350.1 to 420.7)	947.8 (890.7 to 1008.9)	561.7 (491.7 to 631.1)	2.45 (2.20 to 2.75)	2.80 (2.36 to 3.35)
1095	150	331	572.6 (526.1 to 618.5)	1358.5 (1282.6 to 1437.7)	785.9 (698.6 to 875.7)	2.37 (2.14 to 2.61)	2.42 (2.02 to 2.99)
1460	127	246	757.1 (702.6 to 809.1)	1717.4 (1630.8 to 1803.9)	960.3 (854.9 to 1064.3)	2.27 (2.08 to 2.48)	2.15 (1.73 to 2.69)
1825	102	207	931.2 (866.0 to 994.1)	2073.0 (1979.7 to 2181.0)	1141.8 (1022.7 to 1266.7)	2.23 (2.05 to 2.43)	2.28 (1.80 to 2.92)
2190	64	163	1064.4 (992.0 to 1139.5)	2410.1 (2299.7 to 2531.3)	1345.7 (1215.8 to 1482.4)	2.26 (2.08 to 2.46)	2.89 (2.21 to 3.96)
2555	69	115	1241.7 (1159.4 to 1332.5)	2704.1 (2587.2 to 2832.8)	1462.4 (1315.5 to 1610.4)	2.18 (2.01 to 2.37)	1.92 (1.43 to 2.64)
2920	53	92	1415.1 (1330.7 to 1511.1)	3000.9 (2869.8 to 3149.5)	1585.8 (1427.7 to 1748.9)	2.12 (1.95 to 2.28)	2.02 (1.43 to 2.87)
3285	35	67	1568.5 (1462.1 to 1686.4)	3282.7 (3146.8 to 3451.3)	1714.2 (1530.4 to 1908.7)	2.09 (1.93 to 2.28)	2.25 (1.52 to 3.61)
3650	28	33	1735.1 (1620.8 to 1864.1)	3472.0 (3323.4 to 3647.7)	1736.9 (1541.0 to 1930.0)	2.00 (1.84 to 2.17)	1.40 (0.85 to 2.32)
4015	12	23	1851.9 (1708.9 to 2006.8)	3685.8 (3516.9 to 3898.7)	1833.9 (1595.5 to 2063.6)	1.99 (1.82 to 2.19)	2.30 (1.20 to 5.31)
4380	6	11	1946.3 (1793.0 to 2104.6)	3892.2 (3682.2 to 4137.7)	1945.9 (1671.5 to 2239.3)	2.00 (1.80 to 2.22)	***
4745	5	5	2163.3 (1924.0 to 2423.7)	4218.9 (3892.7 to 4679.8)	2055.6 (1623.0 to 2517.9)	1.95 (1.68 to 2.24)	***
5110	0	1	2163.3 (1924.0 to 2423.7)	4549.0 (3961.0 to 5379.4)	2385.7 (1757.6 to 3198.4)	2.10 (1.76 to 2.58)	***

*** IRR/RR was not estimated when there were fewer than 20 events in the risk period or fewer than 5 events in one group. Confidence intervals obtained through percentile bootstrap.

Supplementary Table 14. Estimated risk of death by cause-specific (defined by ICD-10 chapters) comparing treated Tuberculosis cases to non-exposed controls in Females.

	Number of events		Risk per 100,000				
Time - Days	Unexposed	Exposed	Unexposed	Exposed	Risk difference per 100,000 - 95% CI	Risk ratio - 95% CI	Incidence rate ratio - 95% CI
				Cause: All-cause			
30	14	49	32.9 (16.5 to 51.7)	115.0 (84.4 to 145.4)	82.1 (48.0 to 112.7)	3.50 (2.05 to 7.00)	3.50 (2.05 to 7.00)
90	18	95	75.7 (52.0 to 104.2)	341.8 (284.7 to 392.5)	266.0 (207.0 to 323.1)	4.51 (3.18 to 6.68)	5.29 (3.35 to 9.73)
180	41	127	176.5 (139.7 to 217.8)	653.7 (582.1 to 729.4)	477.2 (397.7 to 556.1)	3.70 (2.95 to 4.82)	3.11 (2.31 to 4.56)
365	56	219	321.4 (262.9 to 375.9)	1218.5 (1118.9 to 1322.6)	897.1 (795.5 to 1017.3)	3.79 (3.25 to 4.70)	3.94 (3.08 to 5.49)
730	128	340	690.2 (607.3 to 772.5)	2194.2 (2044.4 to 2344.5)	1504.0 (1353.1 to 1667.4)	3.18 (2.83 to 3.61)	2.69 (2.21 to 3.26)
1095	91	274	996.0 (896.0 to 1100.8)	3110.7 (2924.1 to 3290.5)	2114.8 (1916.6 to 2294.4)	3.12 (2.78 to 3.48)	3.07 (2.43 to 3.89)
1460	86	247	1340.0 (1221.8 to 1473.1)	4096.0 (3883.8 to 4330.3)	2756.0 (2525.5 to 3015.0)	3.06 (2.76 to 3.38)	2.94 (2.37 to 3.77)
1825	75	154	1700.9 (1551.7 to 1849.5)	4837.9 (4595.1 to 5097.1)	3137.0 (2871.9 to 3399.7)	2.84 (2.60 to 3.11)	2.11 (1.64 to 2.84)
2190	73	153	2122.8 (1953.5 to 2309.1)	5718.7 (5440.2 to 5983.4)	3596.0 (3279.5 to 3900.0)	2.69 (2.45 to 2.95)	2.17 (1.65 to 2.89)
2555	54	119	2500.0 (2314.8 to 2730.4)	6565.6 (6247.2 to 6869.4)	4065.6 (3689.6 to 4414.8)	2.63 (2.39 to 2.86)	2.29 (1.68 to 3.15)

2920	58	81	3025.0 (2789.4 to 3306.3)	7300.6 (6933.4 to 7626.2)	4275.6 (3879.6 to 4670.2)	2.41 (2.20 to 2.64)	1.46 (1.08 to 2.04)
3285	30	76	3382.9 (3115.1 to 3689.3)	8191.1 (7777.0 to 8582.2)	4808.3 (4344.3 to 5227.2)	2.42 (2.21 to 2.64)	2.66 (1.77 to 4.31)
3650	24	46	3785.1 (3473.9 to 4117.9)	8984.1 (8508.1 to 9428.5)	5199.0 (4690.0 to 5718.5)	2.37 (2.17 to 2.58)	2.02 (1.26 to 3.40)
4015	17	25	4270.9 (3862.6 to 4664.9)	9704.9 (9155.1 to 10250.1)	5434.1 (4783.3 to 6055.6)	2.27 (2.05 to 2.52)	1.56 (0.89 to 3.17)
4380	10	14	4714.4 (4247.6 to 5210.4)	10390.9 (9711.0 to 10989.4)	5676.6 (4892.2 to 6412.2)	2.20 (1.97 to 2.45)	1.49 (0.68 to 3.52)
4745	5	5	5471.5 (4675.2 to 6371.2)	11116.3 (10213.0 to 12155.6)	5644.8 (4417.7 to 6836.4)	2.03 (1.71 to 2.41)	***
5110	0	0	5471.5 (4675.2 to 6371.2)	11116.3 (10213.0 to 12155.6)	5644.8 (4417.7 to 6836.4)	2.03 (1.71 to 2.41)	***
**Cause: Natural - excluding (HIV/TB and external causes)**							
30	13	34	30.5 (16.4 to 49.3)	79.8 (54.0 to 104.5)	49.3 (19.9 to 79.7)	2.62 (1.49 to 5.38)	2.62 (1.49 to 5.39)
90	16	61	68.6 (47.2 to 96.0)	225.5 (182.8 to 268.2)	156.8 (107.1 to 207.8)	3.28 (2.19 to 4.97)	3.82 (2.32 to 6.97)
180	37	87	159.6 (124.0 to 196.5)	439.2 (383.2 to 497.0)	279.6 (208.1 to 345.7)	2.75 (2.12 to 3.64)	2.36 (1.70 to 3.66)
365	47	150	281.3 (228.9 to 330.7)	826.4 (747.1 to 911.7)	545.1 (461.6 to 648.1)	2.94 (2.46 to 3.66)	3.21 (2.47 to 4.61)
730	112	232	604.0 (521.8 to 681.9)	1494.2 (1375.0 to 1610.6)	890.3 (754.9 to 1025.4)	2.47 (2.16 to 2.87)	2.10 (1.67 to 2.62)
1095	82	212	879.5 (791.1 to 980.2)	2202.6 (2052.0 to 2357.9)	1323.1 (1142.3 to 1481.8)	2.50 (2.20 to 2.83)	2.63 (2.05 to 3.44)
1460	82	181	1207.4 (1089.7 to 1328.6)	2924.8 (2743.0 to 3130.1)	1717.5 (1507.3 to 1948.8)	2.42 (2.17 to 2.71)	2.26 (1.78 to 2.98)
1825	64	129	1515.3 (1381.1 to 1656.4)	3544.9 (3318.6 to 3773.0)	2029.5 (1777.3 to 2269.1)	2.34 (2.09 to 2.58)	2.08 (1.54 to 2.81)
2190	65	120	1891.9 (1739.8 to 2070.6)	4237.2 (3977.3 to 4489.7)	2345.3 (2065.5 to 2585.1)	2.24 (2.01 to 2.47)	1.91 (1.42 to 2.58)
2555	51	99	2249.3 (2064.9 to 2472.4)	4939.9 (4658.1 to 5214.2)	2690.6 (2362.8 to 2972.0)	2.20 (1.98 to 2.40)	2.02 (1.47 to 2.75)
2920	52	67	2720.2 (2486.7 to 2979.4)	5546.9 (5224.4 to 5807.1)	2826.7 (2450.0 to 3184.0)	2.04 (1.84 to 2.23)	1.35 (0.95 to 1.94)
3285	28	62	3053.0 (2792.8 to 3337.6)	6271.4 (5881.2 to 6592.6)	3218.3 (2790.6 to 3593.9)	2.05 (1.86 to 2.25)	2.32 (1.55 to 3.84)
3650	20	37	3388.6 (3115.6 to 3691.9)	6916.1 (6494.7 to 7297.3)	3527.5 (3010.4 to 3965.8)	2.04 (1.84 to 2.24)	1.95 (1.18 to 3.56)
4015	16	21	3843.8 (3454.8 to 4210.6)	7510.8 (7041.7 to 8000.3)	3667.0 (3047.6 to 4247.6)	1.95 (1.76 to 2.19)	1.39 (0.80 to 2.89)
4380	9	13	4248.4 (3774.5 to 4754.9)	8127.0 (7581.4 to 8693.2)	3878.6 (3171.2 to 4591.1)	1.91 (1.69 to 2.17)	1.54 (0.66 to 3.88)
4745	5	4	5005.6 (4248.5 to 5894.9)	8728.7 (7959.0 to 9682.7)	3723.1 (2527.6 to 4756.7)	1.74 (1.44 to 2.09)	***
5110	0	0	5005.6 (4248.5 to 5894.9)	8728.7 (7959.0 to 9682.7)	3723.1 (2527.6 to 4756.7)	1.74 (1.44 to 2.09)	***
**Cause: Cancer**							
30	2	10	4.7 (0.0 to 13.0)	23.5 (9.4 to 37.6)	18.8 (2.3 to 35.2)	***	***
90	3	15	11.9 (2.4 to 23.7)	59.4 (40.2 to 83.4)	47.5 (26.1 to 72.3)	5.01 (2.38 to 24.58)	***
180	3	19	19.1 (7.2 to 33.6)	105.9 (77.0 to 137.2)	86.7 (54.1 to 121.8)	5.53 (2.93 to 14.12)	***
365	11	32	47.5 (28.1 to 70.4)	188.7 (152.8 to 232.6)	141.2 (95.3 to 187.6)	3.97 (2.40 to 6.96)	2.93 (1.55 to 6.25)
730	29	38	131.6 (97.6 to 166.0)	298.0 (245.4 to 349.8)	166.4 (102.3 to 224.9)	2.27 (1.66 to 3.07)	1.33 (0.80 to 2.03)
1095	20	46	199.2 (150.1 to 246.0)	451.3 (382.6 to 520.3)	252.1 (166.0 to 338.1)	2.27 (1.73 to 3.00)	2.34 (1.47 to 4.44)
1460	21	37	283.8 (226.5 to 341.4)	601.1 (517.8 to 686.7)	317.3 (214.3 to 419.3)	2.12 (1.66 to 2.72)	1.81 (1.06 to 3.23)
1825	11	20	334.6 (272.7 to 401.5)	694.8 (603.3 to 797.8)	360.3 (242.4 to 475.5)	2.08 (1.65 to 2.65)	1.87 (0.92 to 3.90)
2190	8	21	379.7 (316.0 to 452.0)	815.0 (710.1 to 928.7)	435.3 (301.3 to 579.0)	2.15 (1.70 to 2.73)	2.72 (1.30 to 7.26)

2555	10	19	451.3 (372.4 to 537.1)	952.5 (829.2 to 1089.9)	501.2 (356.8 to 656.6)	2.11 (1.67 to 2.67)	1.98 (0.91 to 4.59)
2920	12	10	560.3 (460.4 to 673.6)	1046.2 (915.7 to 1193.4)	486.0 (309.1 to 662.7)	1.87 (1.49 to 2.37)	0.87 (0.36 to 2.33)
3285	6	10	629.0 (514.6 to 763.7)	1163.8 (1017.8 to 1326.2)	534.9 (308.9 to 724.5)	1.85 (1.42 to 2.29)	***
3650	8	4	761.5 (617.7 to 925.6)	1232.0 (1083.7 to 1409.8)	470.5 (234.7 to 700.3)	1.62 (1.27 to 2.08)	***
4015	7	5	958.8 (745.8 to 1163.9)	1391.0 (1168.2 to 1623.1)	432.1 (132.0 to 745.4)	1.45 (1.12 to 1.97)	***
4380	1	1	997.7 (776.0 to 1244.3)	1450.3 (1220.8 to 1693.2)	452.6 (119.6 to 787.0)	1.45 (1.10 to 1.97)	***
4745	1	1	1162.0 (821.9 to 1596.3)	1565.3 (1260.4 to 1881.5)	403.4 (-161.7 to 891.8)	1.35 (0.89 to 1.99)	***
5110	0	0	1162.0 (821.9 to 1596.3)	1565.3 (1260.4 to 1881.5)	403.4 (-161.7 to 891.8)	1.35 (0.89 to 1.99)	***
**Cause: Cardiovascular**							
30	4	3	9.4 (2.3 to 18.8)	7.0 (0.0 to 14.1)	-2.3 (-14.1 to 9.4)	***	***
90	5	12	21.3 (8.2 to 35.6)	35.7 (19.0 to 54.8)	14.4 (-8.3 to 35.8)	1.68 (0.76 to 4.87)	***
180	17	14	63.0 (41.1 to 87.3)	70.1 (46.3 to 96.6)	7.0 (-25.9 to 40.4)	1.11 (0.65 to 1.93)	0.83 (0.38 to 1.69)
365	11	30	91.3 (64.0 to 120.5)	147.5 (111.2 to 182.1)	56.2 (10.4 to 102.4)	1.62 (1.11 to 2.41)	2.75 (1.48 to 6.50)
730	29	49	173.9 (132.6 to 219.0)	289.9 (237.1 to 344.7)	116.0 (52.1 to 187.5)	1.67 (1.25 to 2.35)	1.71 (1.09 to 2.93)
1095	24	48	253.3 (203.4 to 312.2)	450.6 (377.3 to 517.3)	197.3 (112.7 to 291.8)	1.78 (1.38 to 2.33)	2.04 (1.28 to 3.43)
1460	34	50	388.6 (324.4 to 457.4)	649.3 (561.1 to 739.1)	260.8 (143.6 to 371.5)	1.67 (1.33 to 2.10)	1.51 (1.00 to 2.39)
1825	19	27	479.9 (401.6 to 561.4)	780.5 (677.7 to 882.0)	300.6 (178.2 to 432.3)	1.63 (1.32 to 2.01)	1.46 (0.79 to 2.66)
2190	21	29	602.2 (509.5 to 703.6)	946.3 (843.7 to 1057.9)	344.1 (198.5 to 485.7)	1.57 (1.30 to 1.91)	1.43 (0.82 to 2.74)
2555	15	16	704.9 (592.6 to 811.9)	1061.5 (937.1 to 1184.7)	356.5 (201.6 to 522.3)	1.51 (1.26 to 1.85)	1.11 (0.52 to 2.51)
2920	11	10	806.6 (680.8 to 929.3)	1151.7 (1021.7 to 1284.6)	345.1 (183.1 to 526.0)	1.43 (1.20 to 1.74)	0.95 (0.35 to 2.26)
3285	17	12	1010.0 (859.8 to 1168.1)	1292.3 (1147.3 to 1463.2)	282.3 (85.1 to 508.5)	1.28 (1.08 to 1.57)	0.74 (0.35 to 1.58)
3650	5	10	1096.4 (923.8 to 1279.7)	1467.0 (1285.2 to 1684.9)	370.6 (142.0 to 628.0)	1.34 (1.12 to 1.65)	***
4015	3	8	1183.0 (995.7 to 1385.4)	1694.2 (1451.6 to 1963.9)	511.2 (200.8 to 823.6)	1.43 (1.15 to 1.77)	***
4380	3	4	1328.6 (1087.8 to 1595.5)	1880.2 (1631.6 to 2219.9)	551.7 (144.4 to 939.2)	1.42 (1.09 to 1.81)	***
4745	1	1	1531.0 (1163.1 to 2023.1)	2017.6 (1661.2 to 2452.6)	486.6 (-185.8 to 1078.2)	1.32 (0.91 to 1.88)	***
5110	0	0	1531.0 (1163.1 to 2023.1)	2017.6 (1661.2 to 2452.6)	486.6 (-185.8 to 1078.2)	1.32 (0.91 to 1.88)	***
**Cause: Endocrine**							
30	0	2	0.0 (0.0 to 0.0)	4.7 (0.0 to 11.8)	4.7 (0.0 to 11.8)	***	***
90	0	3	0.0 (0.0 to 0.0)	11.9 (2.4 to 23.7)	11.9 (2.4 to 23.7)	***	***
180	5	10	12.4 (2.5 to 24.7)	36.5 (19.2 to 56.1)	24.1 (4.4 to 46.2)	***	***
365	6	17	28.0 (12.6 to 45.6)	80.3 (52.4 to 110.2)	52.4 (22.1 to 86.8)	2.87 (1.56 to 6.99)	2.85 (1.19 to 10.57)
730	12	21	62.7 (38.8 to 92.3)	141.4 (103.5 to 180.4)	78.7 (33.0 to 124.2)	2.25 (1.39 to 3.88)	1.77 (0.89 to 4.05)
1095	11	22	100.4 (68.7 to 138.0)	215.5 (168.3 to 265.6)	115.1 (54.1 to 173.1)	2.15 (1.41 to 3.31)	2.04 (0.96 to 4.66)
1460	6	14	124.3 (86.6 to 170.2)	271.4 (216.2 to 328.8)	147.1 (77.9 to 217.4)	2.18 (1.50 to 3.29)	2.39 (1.02 to 7.69)
1825	8	14	163.9 (117.1 to 215.9)	339.1 (274.8 to 407.3)	175.2 (95.7 to 263.5)	2.07 (1.48 to 3.12)	1.80 (0.77 to 5.49)

2190	6	13	199.4 (143.8 to 259.3)	413.8 (339.4 to 492.5)	214.5 (115.8 to 310.0)	2.08 (1.49 to 3.04)	***
2555	4	8	227.0 (166.2 to 296.5)	470.7 (379.1 to 561.4)	243.6 (136.8 to 350.8)	2.07 (1.50 to 2.97)	***
2920	6	13	280.9 (206.3 to 361.0)	589.5 (478.2 to 697.3)	308.6 (177.6 to 435.4)	2.10 (1.53 to 2.98)	***
3285	1	10	292.9 (212.1 to 376.0)	703.1 (580.6 to 839.1)	410.2 (265.7 to 564.1)	2.40 (1.76 to 3.47)	***
3650	0	3	292.9 (212.1 to 376.0)	754.2 (618.8 to 898.4)	461.3 (303.2 to 620.7)	2.57 (1.87 to 3.72)	***
4015	0	2	292.9 (212.1 to 376.0)	799.2 (652.6 to 960.5)	506.3 (332.9 to 685.6)	2.73 (1.97 to 3.92)	***
4380	0	3	292.9 (212.1 to 376.0)	945.3 (735.9 to 1219.0)	652.4 (426.9 to 930.1)	3.23 (2.24 to 4.78)	***
4745	1	0	477.6 (237.7 to 943.8)	945.3 (735.9 to 1219.0)	467.8 (-18.7 to 821.4)	1.98 (0.98 to 4.17)	***
5110	0	0	477.6 (237.7 to 943.8)	945.3 (735.9 to 1219.0)	467.8 (-18.7 to 821.4)	1.98 (0.98 to 4.17)	***
**Cause: Respiratory**							
30	3	9	7.1 (0.0 to 15.4)	21.1 (7.0 to 36.4)	14.0 (-0.1 to 30.5)	***	***
90	2	13	11.8 (2.4 to 23.6)	52.2 (30.8 to 74.6)	40.4 (18.9 to 66.3)	4.42 (1.90 to 23.55)	***
180	5	19	24.1 (11.9 to 38.6)	99.0 (72.2 to 130.7)	74.9 (39.2 to 110.8)	4.11 (2.16 to 8.44)	3.81 (1.51 to 17.07)
365	1	28	26.6 (12.2 to 41.5)	171.1 (132.4 to 211.7)	144.5 (100.1 to 186.4)	6.43 (3.92 to 13.81)	***
730	11	54	58.6 (33.7 to 82.8)	326.2 (267.9 to 387.6)	267.6 (204.3 to 329.0)	5.57 (3.65 to 9.67)	4.97 (2.73 to 11.24)
1095	11	38	95.1 (64.0 to 128.0)	454.0 (386.8 to 528.6)	358.9 (283.9 to 431.2)	4.77 (3.51 to 7.13)	3.52 (1.84 to 8.27)
1460	4	29	111.4 (75.5 to 148.6)	570.5 (492.4 to 663.7)	459.1 (373.5 to 541.2)	5.12 (3.70 to 7.53)	7.43 (3.21 to 37.39)
1825	12	37	170.2 (123.8 to 220.0)	748.9 (655.8 to 864.2)	578.7 (476.3 to 702.3)	4.40 (3.31 to 6.13)	3.18 (1.72 to 6.82)
2190	9	16	223.4 (163.6 to 283.4)	840.7 (743.6 to 970.6)	617.3 (496.8 to 751.1)	3.76 (2.82 to 5.29)	1.84 (0.81 to 5.19)
2555	10	21	295.1 (223.5 to 374.2)	989.5 (862.9 to 1133.4)	694.4 (544.2 to 846.1)	3.35 (2.55 to 4.43)	2.19 (1.04 to 5.47)
2920	6	18	346.8 (263.4 to 439.8)	1152.8 (1005.2 to 1310.7)	805.9 (633.6 to 975.3)	3.32 (2.55 to 4.36)	3.14 (1.37 to 10.47)
3285	1	16	357.9 (274.7 to 455.8)	1342.0 (1157.6 to 1526.6)	984.1 (780.5 to 1179.0)	3.75 (2.81 to 5.01)	***
3650	3	2	414.3 (312.4 to 529.8)	1380.9 (1189.9 to 1579.3)	966.7 (742.5 to 1185.2)	3.33 (2.51 to 4.45)	***
4015	2	1	479.0 (357.9 to 638.2)	1408.9 (1214.2 to 1608.4)	929.9 (674.5 to 1165.7)	2.94 (2.10 to 4.09)	***
4380	2	2	565.3 (391.1 to 766.0)	1507.7 (1291.0 to 1771.2)	942.4 (659.2 to 1237.5)	2.67 (1.90 to 3.92)	***
4745	1	1	665.1 (425.8 to 948.9)	1747.9 (1333.9 to 2376.7)	1082.9 (553.5 to 1748.9)	2.63 (1.64 to 4.49)	***
5110	0	0	665.1 (425.8 to 948.9)	1747.9 (1333.9 to 2376.7)	1082.9 (553.5 to 1748.9)	2.63 (1.64 to 4.49)	***
**Cause: External**							
30	1	2	2.3 (0.0 to 7.0)	4.7 (0.0 to 11.7)	2.3 (-4.7 to 11.7)	***	***
90	2	6	7.1 (0.0 to 15.5)	19.0 (7.2 to 35.6)	11.9 (-0.0 to 28.6)	***	***
180	1	4	9.5 (2.3 to 20.4)	28.9 (14.4 to 46.1)	19.3 (2.5 to 38.5)	***	***
365	7	9	27.8 (12.6 to 48.3)	51.9 (32.3 to 73.6)	24.2 (0.6 to 53.4)	1.87 (1.01 to 4.61)	***
730	10	11	56.7 (32.2 to 83.6)	84.1 (58.3 to 117.6)	27.4 (-6.8 to 64.4)	1.48 (0.89 to 2.83)	1.11 (0.47 to 2.84)
1095	9	9	86.9 (55.3 to 120.1)	114.9 (82.7 to 148.7)	28.0 (-16.2 to 74.7)	1.32 (0.85 to 2.20)	***
1460	4	17	103.1 (70.9 to 142.9)	183.3 (140.2 to 229.3)	80.2 (25.3 to 141.7)	1.78 (1.19 to 2.85)	***

1825	9	8	146.5 (104.4 to 197.3)	222.3 (168.2 to 276.2)	75.8 (8.0 to 145.9)	1.52 (1.04 to 2.29)	***
2190	6	9	180.6 (130.2 to 238.0)	274.4 (214.4 to 337.0)	93.8 (17.3 to 173.8)	1.52 (1.09 to 2.23)	***
2555	1	5	187.1 (133.2 to 245.5)	310.9 (244.7 to 376.8)	123.7 (35.6 to 216.1)	1.66 (1.17 to 2.49)	***
2920	5	3	231.2 (167.4 to 304.8)	338.6 (263.2 to 407.9)	107.3 (8.9 to 202.5)	1.46 (1.03 to 2.10)	***
3285	1	3	244.9 (175.4 to 322.4)	372.3 (292.0 to 451.7)	127.4 (16.5 to 224.0)	1.52 (1.05 to 2.17)	***
3650	2	0	280.2 (190.3 to 377.1)	372.3 (292.0 to 451.7)	92.1 (-23.1 to 199.0)	1.33 (0.94 to 1.91)	***
4015	0	3	280.2 (190.3 to 377.1)	463.7 (337.8 to 606.6)	183.5 (30.2 to 349.6)	1.65 (1.09 to 2.56)	***
4380	0	0	280.2 (190.3 to 377.1)	463.7 (337.8 to 606.6)	183.5 (30.2 to 349.6)	1.65 (1.09 to 2.56)	***
4745	0	0	280.2 (190.3 to 377.1)	463.7 (337.8 to 606.6)	183.5 (30.2 to 349.6)	1.65 (1.09 to 2.56)	***
5110	0	0	280.2 (190.3 to 377.1)	463.7 (337.8 to 606.6)	183.5 (30.2 to 349.6)	1.65 (1.09 to 2.56)	***

*** IRR/RR was not estimated when there were fewer than 20 events in the risk period or fewer than 5 events in one group. Confidence intervals obtained through percentile bootstrap.

Supplementary Table 15. Estimated risk of death by cause-specific (defined by ICD-10 chapters) comparing treated Tuberculosis cases to non-exposed controls in Males.

	Number of events		Risk per 100,000				
**Time - Days**	**Unexposed**	**Exposed**	**Unexposed**	**Exposed**	**Risk difference per 100,000 - 95% CI**	**Risk ratio - 95% CI**	**Incidence rate ratio - 95% CI**
				**Cause: All-cause**			
30	35	109	51.0 (37.8 to 68.6)	158.8 (129.6 to 187.9)	107.8 (74.3 to 136.9)	3.11 (2.20 to 4.46)	3.12 (2.20 to 4.46)
90	74	264	160.8 (135.0 to 191.9)	551.2 (492.2 to 609.6)	390.4 (326.6 to 451.5)	3.43 (2.76 to 4.21)	3.58 (2.76 to 4.65)
180	97	342	309.3 (272.7 to 350.8)	1075.6 (1001.9 to 1144.4)	766.4 (677.9 to 856.0)	3.48 (3.00 to 4.01)	3.55 (2.92 to 4.41)
365	189	624	618.0 (555.3 to 675.6)	2093.7 (1986.6 to 2196.7)	1475.7 (1357.3 to 1585.9)	3.39 (3.05 to 3.78)	3.34 (2.85 to 3.94)
730	336	1005	1238.5 (1154.4 to 1318.1)	3949.5 (3810.5 to 4090.4)	2711.0 (2544.8 to 2865.5)	3.19 (2.95 to 3.43)	3.05 (2.71 to 3.46)
1095	327	794	1959.9 (1843.5 to 2086.0)	5698.1 (5515.7 to 5871.2)	3738.1 (3523.1 to 3942.2)	2.91 (2.70 to 3.11)	2.51 (2.21 to 2.87)
1460	284	681	2712.0 (2564.3 to 2861.3)	7513.0 (7296.1 to 7718.9)	4801.0 (4547.2 to 5065.6)	2.77 (2.60 to 2.95)	2.50 (2.22 to 2.86)
1825	230	502	3457.9 (3295.6 to 3621.2)	9142.3 (8902.8 to 9391.6)	5684.4 (5401.1 to 5967.9)	2.64 (2.52 to 2.80)	2.30 (1.99 to 2.67)
2190	160	408	4080.9 (3892.0 to 4288.6)	10739.2 (10448.2 to 11031.8)	6658.3 (6294.8 to 6990.0)	2.63 (2.49 to 2.78)	2.71 (2.23 to 3.30)
2555	133	284	4717.8 (4509.4 to 4947.6)	12097.7 (11774.5 to 12398.2)	7379.9 (7019.1 to 7771.7)	2.56 (2.43 to 2.70)	2.29 (1.89 to 2.82)
2920	108	249	5387.5 (5136.2 to 5627.7)	13632.6 (13274.6 to 14028.1)	8245.0 (7837.3 to 8700.2)	2.53 (2.40 to 2.67)	2.50 (2.00 to 3.15)
3285	92	176	6156.9 (5860.2 to 6442.7)	15082.3 (14696.2 to 15509.6)	8925.4 (8417.3 to 9429.5)	2.45 (2.32 to 2.60)	2.09 (1.64 to 2.78)
3650	51	107	6762.3 (6423.5 to 7094.2)	16354.9 (15909.7 to 16847.1)	9592.6 (9045.0 to 10170.4)	2.42 (2.28 to 2.55)	2.31 (1.72 to 3.33)

4015	36	74	7549.3 (7115.3 to 7995.0)	17839.0 (17305.6 to 18407.8)	10289.7 (9587.1 to 10999.0)	2.36 (2.22 to 2.52)	2.29 (1.60 to 3.65)
4380	13	32	8151.7 (7567.6 to 8661.2)	19258.9 (18517.6 to 20049.1)	11107.2 (10181.4 to 12042.6)	2.36 (2.19 to 2.54)	2.76 (1.56 to 5.55)
4745	7	1	9239.3 (8230.2 to 10192.3)	19361.0 (18623.6 to 20173.7)	10121.6 (8813.3 to 11336.8)	2.10 (1.87 to 2.35)	***
5110	2	1	10353.8 (8812.3 to 12245.0)	19928.8 (18804.2 to 21665.0)	9575.0 (7283.1 to 11904.2)	1.92 (1.61 to 2.30)	***
**Cause: Natural - excluding (HIV/TB and external causes)**							
30	26	72	37.9 (24.8 to 52.5)	104.9 (82.3 to 126.8)	67.0 (40.8 to 91.8)	2.77 (1.85 to 4.24)	2.77 (1.85 to 4.25)
90	49	179	110.6 (88.4 to 135.7)	370.9 (323.6 to 416.4)	260.3 (210.0 to 313.3)	3.35 (2.65 to 4.25)	3.66 (2.68 to 5.01)
180	67	212	213.2 (182.2 to 244.4)	695.8 (633.8 to 753.5)	482.6 (415.3 to 546.0)	3.26 (2.75 to 3.86)	3.18 (2.49 to 4.09)
365	125	411	417.0 (373.9 to 465.5)	1366.4 (1272.8 to 1446.8)	949.4 (845.7 to 1039.6)	3.28 (2.89 to 3.69)	3.32 (2.75 to 3.99)
730	229	648	840.4 (773.6 to 913.1)	2565.1 (2440.4 to 2690.2)	1724.7 (1573.1 to 1861.4)	3.05 (2.75 to 3.35)	2.89 (2.49 to 3.34)
1095	238	527	1364.3 (1266.0 to 1470.0)	3725.5 (3571.8 to 3872.3)	2361.2 (2176.4 to 2533.5)	2.73 (2.49 to 2.96)	2.29 (1.95 to 2.66)
1460	212	460	1924.9 (1805.6 to 2051.1)	4951.8 (4772.3 to 5135.5)	3026.9 (2814.8 to 3246.5)	2.57 (2.39 to 2.76)	2.26 (1.97 to 2.63)
1825	179	355	2504.7 (2359.5 to 2640.8)	6105.6 (5892.2 to 6351.6)	3600.9 (3353.4 to 3850.8)	2.44 (2.28 to 2.60)	2.09 (1.76 to 2.54)
2190	109	281	2926.9 (2771.8 to 3095.2)	7207.1 (6973.2 to 7490.5)	4280.1 (4010.8 to 4558.8)	2.46 (2.32 to 2.61)	2.74 (2.19 to 3.48)
2555	93	201	3371.3 (3196.0 to 3565.0)	8167.3 (7911.1 to 8462.8)	4796.0 (4507.1 to 5099.0)	2.42 (2.27 to 2.57)	2.32 (1.85 to 2.99)
2920	77	179	3848.4 (3654.8 to 4073.4)	9272.9 (8988.7 to 9606.0)	5424.5 (5082.3 to 5790.4)	2.41 (2.27 to 2.56)	2.52 (1.98 to 3.25)
3285	68	129	4414.6 (4174.7 to 4660.3)	10338.9 (10013.2 to 10738.0)	5924.3 (5512.4 to 6328.5)	2.34 (2.19 to 2.48)	2.07 (1.62 to 2.87)
3650	40	90	4886.4 (4608.0 to 5172.8)	11413.3 (11026.9 to 11849.3)	6527.0 (6055.9 to 7008.2)	2.34 (2.19 to 2.49)	2.48 (1.77 to 3.71)
4015	26	57	5460.2 (5072.0 to 5845.1)	12579.8 (12105.0 to 13118.4)	7119.6 (6500.5 to 7729.6)	2.30 (2.14 to 2.49)	2.44 (1.61 to 4.04)
4380	12	25	6013.0 (5504.6 to 6522.9)	13640.3 (13043.4 to 14308.4)	7627.4 (6852.4 to 8488.6)	2.27 (2.07 to 2.48)	2.33 (1.18 to 5.05)
4745	7	1	7100.6 (6175.3 to 8017.2)	13742.4 (13160.0 to 14432.3)	6641.8 (5425.8 to 7753.9)	1.94 (1.68 to 2.24)	***
5110	2	0	8215.1 (6701.5 to 10128.7)	13742.4 (13160.0 to 14432.3)	5527.3 (3604.5 to 7226.3)	1.67 (1.36 to 2.07)	***
**Cause: Cancer**							
30	5	19	7.3 (1.5 to 14.6)	27.7 (16.0 to 40.7)	20.4 (7.2 to 34.9)	3.79 (1.64 to 17.47)	3.80 (1.65 to 17.53)
90	5	56	14.7 (5.9 to 23.5)	110.9 (85.8 to 134.8)	96.2 (68.0 to 122.7)	7.53 (4.42 to 17.57)	11.23 (5.43 to 49.16)
180	15	54	37.7 (24.1 to 52.5)	193.6 (161.4 to 228.0)	155.9 (121.3 to 191.0)	5.14 (3.41 to 8.30)	3.62 (2.21 to 6.61)
365	27	91	81.8 (60.3 to 105.0)	342.1 (296.7 to 390.3)	260.3 (210.9 to 305.9)	4.18 (3.16 to 5.87)	3.41 (2.22 to 5.53)
730	31	147	139.2 (110.1 to 168.0)	613.3 (557.2 to 678.0)	474.2 (409.7 to 542.1)	4.41 (3.55 to 5.52)	4.84 (3.35 to 7.34)
1095	45	89	237.3 (199.3 to 279.2)	808.4 (731.7 to 879.3)	571.1 (484.9 to 659.4)	3.41 (2.78 to 4.12)	2.04 (1.43 to 3.01)
1460	43	83	351.9 (303.4 to 407.5)	1029.2 (938.5 to 1109.5)	677.3 (577.5 to 777.7)	2.93 (2.46 to 3.49)	2.01 (1.39 to 2.94)
1825	31	73	452.0 (387.0 to 514.0)	1265.6 (1163.5 to 1360.0)	813.6 (706.2 to 933.9)	2.80 (2.41 to 3.30)	2.48 (1.70 to 3.94)
2190	20	49	529.1 (455.6 to 595.9)	1457.2 (1344.3 to 1566.1)	928.1 (803.3 to 1059.8)	2.75 (2.40 to 3.24)	2.61 (1.56 to 4.77)
2555	11	35	581.9 (503.2 to 651.2)	1623.6 (1500.1 to 1740.6)	1041.6 (901.5 to 1191.6)	2.79 (2.44 to 3.25)	3.42 (1.89 to 7.51)
2920	18	33	691.7 (594.7 to 773.9)	1826.0 (1686.3 to 1964.2)	1134.3 (977.7 to 1301.7)	2.64 (2.28 to 3.14)	1.99 (1.14 to 3.53)
3285	14	25	808.1 (692.8 to 917.2)	2031.7 (1881.7 to 2212.0)	1223.6 (1052.2 to 1415.7)	2.51 (2.18 to 2.99)	1.95 (0.99 to 4.10)

3650	9	23	914.5 (776.1 to 1051.8)	2303.8 (2117.5 to 2516.6)	1389.3 (1156.5 to 1607.5)	2.52 (2.13 to 3.00)	2.82 (1.25 to 7.28)
4015	5	5	1014.7 (865.5 to 1183.2)	2392.8 (2182.6 to 2622.2)	1378.1 (1097.9 to 1646.7)	2.36 (1.94 to 2.82)	***
4380	2	8	1098.9 (916.9 to 1323.8)	2695.7 (2398.5 to 3027.4)	1596.8 (1204.2 to 1973.1)	2.45 (1.93 to 3.04)	***
4745	2	0	1435.9 (1008.2 to 1957.0)	2695.7 (2398.5 to 3027.4)	1259.7 (660.9 to 1788.6)	1.88 (1.35 to 2.70)	***
5110	0	0	1435.9 (1008.2 to 1957.0)	2695.7 (2398.5 to 3027.4)	1259.7 (660.9 to 1788.6)	1.88 (1.35 to 2.70)	***
				**Cause: Cardiovascular**			
30	7	16	10.2 (4.4 to 18.3)	23.3 (12.4 to 35.7)	13.1 (-0.1 to 26.2)	2.28 (1.00 to 7.17)	2.29 (1.00 to 7.18)
90	23	32	44.3 (29.6 to 62.0)	70.8 (50.1 to 90.7)	26.5 (0.8 to 51.0)	1.60 (1.02 to 2.54)	1.39 (0.81 to 2.45)
180	24	40	81.1 (61.5 to 103.0)	132.1 (104.5 to 157.2)	51.0 (18.7 to 84.4)	1.63 (1.20 to 2.27)	1.68 (1.04 to 2.96)
365	34	85	136.6 (108.6 to 165.8)	270.9 (231.3 to 312.9)	134.2 (85.9 to 182.7)	1.98 (1.54 to 2.63)	2.53 (1.59 to 4.08)
730	86	142	295.7 (255.1 to 338.1)	533.7 (475.2 to 592.7)	238.1 (161.5 to 307.4)	1.81 (1.49 to 2.15)	1.69 (1.30 to 2.16)
1095	84	114	483.1 (427.8 to 550.5)	786.7 (714.0 to 862.0)	303.5 (212.1 to 395.9)	1.63 (1.40 to 1.90)	1.40 (1.09 to 1.87)
1460	61	96	644.9 (576.1 to 715.5)	1043.4 (955.6 to 1133.1)	398.5 (298.6 to 501.5)	1.62 (1.43 to 1.82)	1.64 (1.20 to 2.26)
1825	55	71	822.2 (740.5 to 900.7)	1274.6 (1168.4 to 1382.9)	452.4 (330.5 to 590.5)	1.55 (1.38 to 1.75)	1.36 (0.96 to 1.92)
2190	40	69	976.7 (886.5 to 1071.3)	1544.9 (1420.8 to 1666.6)	568.3 (421.1 to 704.7)	1.58 (1.41 to 1.77)	1.84 (1.28 to 2.80)
2555	40	55	1168.8 (1057.8 to 1282.5)	1805.3 (1659.9 to 1942.3)	636.5 (474.4 to 792.4)	1.54 (1.38 to 1.72)	1.48 (1.00 to 2.30)
2920	21	37	1299.5 (1182.8 to 1423.8)	2032.0 (1880.5 to 2188.5)	732.5 (562.0 to 900.3)	1.56 (1.40 to 1.74)	1.91 (1.16 to 3.36)
3285	27	33	1528.2 (1374.3 to 1670.3)	2306.3 (2121.3 to 2502.4)	778.1 (553.0 to 1010.9)	1.51 (1.34 to 1.71)	1.34 (0.81 to 2.32)
3650	14	14	1690.9 (1522.7 to 1863.3)	2470.9 (2275.2 to 2686.6)	780.1 (537.6 to 1063.3)	1.46 (1.29 to 1.67)	1.10 (0.48 to 2.51)
4015	14	15	2016.6 (1777.2 to 2250.6)	2791.0 (2533.3 to 3036.4)	774.4 (466.8 to 1139.4)	1.38 (1.22 to 1.62)	1.19 (0.60 to 2.46)
4380	4	5	2229.5 (1921.9 to 2596.4)	3007.4 (2724.7 to 3350.8)	777.9 (334.4 to 1217.5)	1.35 (1.13 to 1.61)	***
4745	3	0	2672.7 (2128.3 to 3288.6)	3007.4 (2724.7 to 3350.8)	334.8 (-309.8 to 967.1)	1.13 (0.90 to 1.42)	***
5110	0	0	2672.7 (2128.3 to 3288.6)	3007.4 (2724.7 to 3350.8)	334.8 (-309.8 to 967.1)	1.13 (0.90 to 1.42)	***
				**Cause: Endocrine**			
30	3	6	4.4 (0.0 to 10.2)	8.7 (2.9 to 16.0)	4.3 (-3.0 to 13.1)	***	***
90	5	8	11.8 (4.4 to 19.3)	20.6 (10.3 to 32.4)	8.8 (-4.4 to 22.1)	1.75 (0.77 to 4.94)	***
180	5	14	19.5 (9.1 to 29.9)	42.1 (28.4 to 58.8)	22.6 (6.0 to 41.9)	2.16 (1.23 to 4.54)	***
365	10	20	35.3 (21.5 to 49.1)	74.8 (54.6 to 97.0)	39.5 (15.1 to 66.1)	2.12 (1.34 to 3.57)	2.02 (0.93 to 5.07)
730	16	47	64.9 (45.9 to 86.6)	162.1 (128.9 to 196.2)	97.3 (59.6 to 136.3)	2.50 (1.73 to 3.69)	3.00 (1.76 to 5.55)
1095	13	41	92.8 (69.1 to 116.4)	252.2 (214.5 to 294.3)	159.4 (113.0 to 205.2)	2.72 (2.04 to 3.77)	3.26 (1.87 to 6.85)
1460	16	35	134.5 (102.1 to 165.7)	345.4 (294.7 to 399.6)	211.0 (153.0 to 273.6)	2.57 (1.99 to 3.48)	2.28 (1.32 to 4.64)
1825	12	32	173.6 (132.9 to 210.4)	451.6 (389.6 to 519.2)	278.1 (207.0 to 354.3)	2.60 (2.02 to 3.47)	2.81 (1.43 to 6.75)
2190	6	25	196.1 (151.2 to 238.3)	551.7 (479.6 to 626.5)	355.7 (270.3 to 442.8)	2.81 (2.19 to 3.71)	4.44 (1.95 to 15.42)
2555	7	15	228.4 (180.7 to 279.5)	624.7 (546.6 to 709.4)	396.3 (303.1 to 492.8)	2.74 (2.15 to 3.56)	2.30 (0.97 to 7.21)
2920	2	7	240.8 (189.4 to 294.3)	668.5 (587.0 to 758.9)	427.8 (328.6 to 532.8)	2.78 (2.20 to 3.62)	***

3285	4	11	273.4 (213.2 to 340.4)	758.9 (661.0 to 865.2)	485.6 (370.5 to 611.6)	2.78 (2.14 to 3.66)	***
3650	3	6	308.0 (237.5 to 388.8)	826.8 (718.0 to 939.7)	518.8 (382.2 to 654.3)	2.68 (2.02 to 3.51)	***
4015	1	4	329.9 (252.4 to 423.6)	903.8 (775.1 to 1041.8)	573.9 (418.3 to 737.9)	2.74 (2.06 to 3.75)	***
4380	1	2	387.7 (265.8 to 545.7)	987.6 (817.8 to 1175.2)	599.9 (370.3 to 828.8)	2.55 (1.72 to 3.92)	***
4745	0	0	387.7 (265.8 to 545.7)	987.6 (817.8 to 1175.2)	599.9 (370.3 to 828.8)	2.55 (1.72 to 3.92)	***
5110	0	0	387.7 (265.8 to 545.7)	987.6 (817.8 to 1175.2)	599.9 (370.3 to 828.8)	2.55 (1.72 to 3.92)	***
**Cause: Respiratory**							
30	1	14	1.4 (0.0 to 4.4)	20.4 (11.6 to 30.7)	19.0 (9.5 to 30.6)	***	***
90	3	31	5.9 (1.4 to 11.9)	66.5 (48.9 to 87.9)	60.7 (41.5 to 80.7)	***	***
180	9	47	19.7 (10.5 to 31.7)	138.6 (111.6 to 164.7)	118.9 (90.9 to 148.2)	7.03 (4.18 to 14.00)	5.25 (2.77 to 12.46)
365	16	85	45.9 (31.1 to 63.1)	277.2 (233.8 to 318.6)	231.3 (187.3 to 273.1)	6.04 (4.25 to 8.82)	5.37 (3.20 to 10.22)
730	28	127	98.5 (73.0 to 127.5)	513.2 (453.8 to 570.5)	414.7 (346.0 to 478.1)	5.21 (3.83 to 7.21)	4.63 (3.01 to 7.39)
1095	26	110	155.5 (122.2 to 192.1)	756.2 (690.2 to 830.7)	600.7 (524.3 to 682.6)	4.86 (3.83 to 6.42)	4.37 (2.90 to 6.92)
1460	21	105	211.0 (169.8 to 256.6)	1036.1 (942.1 to 1123.4)	825.1 (723.5 to 929.7)	4.91 (3.96 to 6.23)	5.21 (3.48 to 8.83)
1825	23	49	284.6 (227.1 to 341.8)	1196.7 (1092.9 to 1293.6)	912.1 (809.5 to 1031.2)	4.20 (3.46 to 5.24)	2.24 (1.50 to 3.91)
2190	12	49	331.9 (274.3 to 396.8)	1388.2 (1274.1 to 1498.5)	1056.2 (927.4 to 1180.5)	4.18 (3.47 to 5.19)	4.35 (2.50 to 9.18)
2555	11	34	384.9 (322.2 to 460.2)	1551.5 (1410.4 to 1674.8)	1166.6 (1016.0 to 1308.8)	4.03 (3.35 to 4.91)	3.32 (1.76 to 7.05)
2920	10	35	448.0 (371.5 to 534.9)	1768.6 (1620.7 to 1916.1)	1320.6 (1152.5 to 1491.4)	3.95 (3.29 to 4.84)	3.79 (2.00 to 9.41)
3285	5	19	490.6 (409.9 to 580.5)	1925.9 (1758.7 to 2103.0)	1435.3 (1257.0 to 1631.2)	3.93 (3.25 to 4.80)	4.16 (1.83 to 18.59)
3650	1	23	505.4 (422.8 to 600.2)	2210.8 (2019.6 to 2402.8)	1705.4 (1494.6 to 1929.4)	4.37 (3.63 to 5.44)	***
4015	2	13	546.7 (435.9 to 658.2)	2471.9 (2234.2 to 2717.5)	1925.2 (1662.8 to 2207.3)	4.52 (3.68 to 5.71)	***
4380	0	5	546.7 (435.9 to 658.2)	2698.3 (2424.6 to 3030.4)	2151.6 (1847.7 to 2493.6)	4.94 (3.98 to 6.22)	***
4745	0	1	546.7 (435.9 to 658.2)	2800.4 (2485.4 to 3208.7)	2253.7 (1916.2 to 2651.0)	5.12 (4.16 to 6.51)	***
5110	0	0	546.7 (435.9 to 658.2)	2800.4 (2485.4 to 3208.7)	2253.7 (1916.2 to 2651.0)	5.12 (4.16 to 6.51)	***
**Cause: External**							
30	9	19	13.1 (5.8 to 21.2)	27.6 (16.0 to 39.3)	14.5 (-0.0 to 29.1)	2.11 (1.00 to 5.35)	2.11 (1.00 to 5.36)
90	23	43	47.2 (31.1 to 62.8)	91.6 (70.9 to 115.4)	44.4 (19.2 to 72.4)	1.94 (1.31 to 3.06)	1.87 (1.12 to 3.17)
180	29	70	91.6 (69.0 to 114.1)	199.1 (167.3 to 232.6)	107.5 (66.9 to 151.8)	2.17 (1.64 to 3.03)	2.43 (1.62 to 3.88)
365	61	119	191.5 (156.4 to 225.7)	393.7 (343.2 to 441.9)	202.2 (136.5 to 261.3)	2.06 (1.63 to 2.55)	1.97 (1.44 to 2.79)
730	105	229	384.9 (332.0 to 436.2)	815.4 (745.6 to 888.0)	430.4 (342.1 to 517.3)	2.12 (1.83 to 2.50)	2.23 (1.79 to 2.83)
1095	86	159	575.7 (507.5 to 640.2)	1165.6 (1087.8 to 1257.2)	589.9 (489.5 to 704.9)	2.02 (1.79 to 2.33)	1.91 (1.49 to 2.51)
1460	63	135	743.4 (662.9 to 821.2)	1525.6 (1419.2 to 1633.0)	782.2 (664.5 to 924.6)	2.05 (1.83 to 2.34)	2.23 (1.72 to 2.99)
1825	51	102	909.4 (818.7 to 996.6)	1856.6 (1738.7 to 1985.2)	947.1 (806.4 to 1104.3)	2.04 (1.83 to 2.31)	2.11 (1.59 to 2.91)
2190	42	84	1074.6 (976.5 to 1174.6)	2184.8 (2045.8 to 2329.5)	1110.2 (946.5 to 1307.4)	2.03 (1.80 to 2.28)	2.13 (1.47 to 3.19)
2555	39	57	1262.6 (1140.7 to 1379.2)	2458.6 (2305.8 to 2616.6)	1196.0 (1009.7 to 1393.6)	1.95 (1.74 to 2.19)	1.57 (1.02 to 2.35)

2920	31	46	1455.3 (1312.4 to 1599.3)	2739.4 (2580.9 to 2912.2)	1284.1 (1063.2 to 1506.3)	1.88 (1.68 to 2.12)	1.61 (0.97 to 2.66)
3285	21	30	1631.9 (1466.4 to 1799.6)	2988.2 (2805.3 to 3181.4)	1356.3 (1109.5 to 1622.4)	1.83 (1.64 to 2.07)	1.56 (0.87 to 2.90)
3650	11	13	1765.5 (1597.3 to 1940.4)	3140.3 (2940.8 to 3354.4)	1374.7 (1093.9 to 1682.7)	1.78 (1.56 to 2.02)	1.30 (0.53 to 3.01)
4015	8	12	1938.7 (1726.8 to 2156.7)	3360.4 (3118.5 to 3596.0)	1421.7 (1099.0 to 1747.5)	1.73 (1.52 to 1.99)	1.67 (0.66 to 5.56)
4380	1	6	1988.3 (1750.3 to 2230.9)	3681.1 (3330.2 to 4083.1)	1692.7 (1264.0 to 2120.9)	1.85 (1.59 to 2.16)	***
4745	0	0	1988.3 (1750.3 to 2230.9)	3681.1 (3330.2 to 4083.1)	1692.7 (1264.0 to 2120.9)	1.85 (1.59 to 2.16)	***
5110	0	1	1988.3 (1750.3 to 2230.9)	4248.9 (3403.8 to 5785.5)	2260.6 (1361.0 to 3807.0)	2.14 (1.65 to 2.97)	***

*** IRR/RR was not estimated when there were fewer than 20 events in the risk period or fewer than 5 events in one group. Confidence intervals obtained through percentile bootstrap.

Supplementary Table 16. Estimated risk of death by cause-specific comparing diagnosed Tuberculosis cases to household contacts.

	Number of events		Risk per 100,000				
**Time - Days**	**Unexposed**	**Exposed**	**Unexposed**	**Exposed**	**Risk difference per 100,000 - 95% CI**	**Risk ratio - 95% CI**	**Incidence rate ratio - 95% CI**
				**Cause: All-cause**			
30	3	201	23.3 (0.0 to 46.6)	1559.8 (1357.7 to 1787.5)	1536.5 (1335.5 to 1776.2)	***	***
90	9	96	94.6 (47.0 to 149.7)	2321.8 (2075.6 to 2599.8)	2227.2 (1989.3 to 2524.0)	24.54 (15.43 to 53.29)	10.88 (6.16 to 29.24)
180	11	63	186.0 (119.8 to 266.6)	2842.6 (2560.8 to 3135.9)	2656.6 (2379.3 to 2957.1)	15.28 (10.56 to 24.37)	5.88 (3.40 to 12.14)
365	16	91	328.8 (235.6 to 425.3)	3645.8 (3326.1 to 3979.8)	3317.0 (2989.8 to 3640.0)	11.09 (8.44 to 15.83)	5.87 (3.58 to 10.86)
730	47	125	805.6 (643.9 to 975.7)	4894.8 (4507.6 to 5308.8)	4089.1 (3680.1 to 4477.2)	6.08 (4.95 to 7.60)	2.77 (1.98 to 3.77)
1095	25	63	1107.4 (909.1 to 1332.4)	5658.5 (5234.9 to 6091.2)	4551.1 (4088.0 to 4981.4)	5.11 (4.21 to 6.24)	2.64 (1.76 to 4.67)
1460	23	49	1441.9 (1191.2 to 1687.0)	6374.7 (5973.2 to 6840.5)	4932.8 (4433.0 to 5438.7)	4.42 (3.69 to 5.36)	2.24 (1.41 to 3.94)
1825	16	44	1725.7 (1451.2 to 1993.1)	7150.2 (6685.7 to 7655.2)	5424.5 (4884.0 to 6003.5)	4.14 (3.53 to 4.95)	2.91 (1.75 to 5.59)
2190	16	41	2086.2 (1773.6 to 2407.3)	8060.6 (7508.9 to 8610.2)	5974.5 (5332.3 to 6593.2)	3.86 (3.30 to 4.62)	2.72 (1.61 to 5.56)

2555	9	21	2335.7 (1965.7 to 2699.7)	8633.7 (8076.8 to 9204.4)	6298.0 (5632.3 to 6966.4)	3.70 (3.15 to 4.45)	2.49 (1.20 to 7.34)
2920	8	17	2622.4 (2215.2 to 3051.6)	9242.1 (8596.0 to 9952.4)	6619.8 (5903.2 to 7431.9)	3.52 (3.03 to 4.30)	2.28 (0.97 to 7.38)
3285	6	15	2921.1 (2474.7 to 3373.6)	9945.5 (9234.3 to 10800.1)	7024.4 (6217.0 to 7905.1)	3.40 (2.93 to 4.13)	2.69 (1.08 to 10.24)
3650	10	6	3583.9 (3010.6 to 4258.7)	10331.0 (9496.0 to 11249.4)	6747.1 (5809.2 to 7727.7)	2.88 (2.44 to 3.44)	***
4015	4	8	4065.8 (3335.8 to 4917.4)	11265.0 (10312.0 to 12373.4)	7199.3 (6024.3 to 8481.1)	2.77 (2.28 to 3.44)	***
4380	3	0	5050.5 (3832.2 to 6627.1)	11265.0 (10312.0 to 12373.4)	6214.5 (4444.7 to 7821.7)	2.23 (1.70 to 2.90)	***
4745	0	0	5050.5 (3832.2 to 6627.1)	11265.0 (10312.0 to 12373.4)	6214.5 (4444.7 to 7821.7)	2.23 (1.70 to 2.90)	***
5110	0	0	5050.5 (3832.2 to 6627.1)	11265.0 (10312.0 to 12373.4)	6214.5 (4444.7 to 7821.7)	2.23 (1.70 to 2.90)	***
				**Cause: Natural-No HIV/TB**			
30	1	54	7.8 (0.0 to 23.4)	419.7 (310.8 to 528.1)	411.9 (303.0 to 520.4)	***	***
90	4	27	39.4 (11.6 to 78.8)	633.7 (504.3 to 759.1)	594.3 (461.5 to 728.2)	16.09 (7.59 to 61.17)	***
180	2	18	56.0 (19.8 to 97.2)	782.1 (636.3 to 922.8)	726.1 (574.0 to 876.0)	13.96 (7.57 to 39.73)	***
365	4	28	92.2 (48.8 to 153.2)	1028.8 (856.1 to 1201.4)	936.5 (761.8 to 1117.0)	11.15 (6.49 to 23.11)	7.00 (2.83 to 35.00)
730	11	33	203.1 (123.4 to 291.5)	1358.8 (1132.5 to 1553.9)	1155.7 (921.7 to 1379.5)	6.69 (4.51 to 11.29)	3.00 (1.59 to 6.80)
1095	7	18	289.9 (190.2 to 401.3)	1574.6 (1343.5 to 1784.6)	1284.7 (1032.7 to 1516.5)	5.43 (3.81 to 8.26)	2.57 (1.19 to 7.33)
1460	6	28	374.5 (259.1 to 502.0)	1982.7 (1738.4 to 2243.9)	1608.1 (1323.9 to 1910.1)	5.29 (3.80 to 7.85)	4.67 (2.33 to 16.76)
1825	10	12	551.2 (385.5 to 718.8)	2186.4 (1912.8 to 2465.3)	1635.2 (1316.5 to 1968.2)	3.97 (2.94 to 5.70)	1.20 (0.53 to 3.07)
2190	8	13	733.7 (519.4 to 954.3)	2476.8 (2123.4 to 2799.7)	1743.1 (1314.1 to 2136.3)	3.38 (2.52 to 4.85)	1.63 (0.62 to 4.84)
2555	2	10	790.5 (575.2 to 1023.2)	2748.6 (2373.4 to 3113.4)	1958.2 (1496.4 to 2430.7)	3.48 (2.59 to 4.90)	***
2920	5	6	973.7 (692.1 to 1271.3)	2957.5 (2531.3 to 3356.3)	1983.8 (1479.8 to 2504.8)	3.04 (2.24 to 4.41)	***
3285	3	7	1116.7 (793.1 to 1479.6)	3285.1 (2836.6 to 3772.7)	2168.3 (1569.3 to 2731.6)	2.94 (2.12 to 4.25)	***
3650	4	3	1381.2 (937.6 to 1816.6)	3487.8 (3028.9 to 4025.2)	2106.6 (1431.4 to 2766.6)	2.53 (1.83 to 3.66)	***
4015	3	4	1762.2 (1198.0 to 2430.3)	3996.0 (3324.4 to 4747.9)	2233.8 (1260.8 to 3228.4)	2.27 (1.51 to 3.50)	***
4380	2	0	2453.5 (1451.5 to 3683.1)	3996.0 (3324.4 to 4747.9)	1542.5 (-14.2 to 2811.3)	1.63 (1.00 to 2.83)	***
4745	0	0	2453.5 (1451.5 to 3683.1)	3996.0 (3324.4 to 4747.9)	1542.5 (-14.2 to 2811.3)	1.63 (1.00 to 2.83)	***
5110	0	0	2453.5 (1451.5 to 3683.1)	3996.0 (3324.4 to 4747.9)	1542.5 (-14.2 to 2811.3)	1.63 (1.00 to 2.83)	***

**Cause: External**							
30	0	5	0.0 (0.0 to 0.0)	39.0 (7.8 to 74.3)	39.0 (7.8 to 74.3)	***	***
90	4	8	31.9 (7.8 to 63.9)	102.9 (47.4 to 158.5)	71.0 (7.6 to 138.4)	***	***
180	8	16	98.5 (48.8 to 155.8)	235.5 (161.8 to 316.5)	137.0 (34.4 to 242.8)	2.39 (1.26 to 5.50)	2.00 (0.90 to 5.67)
365	10	22	187.8 (110.9 to 260.8)	430.1 (320.6 to 558.1)	242.3 (120.0 to 389.2)	2.29 (1.48 to 4.10)	2.20 (1.03 to 6.00)
730	36	55	553.8 (420.1 to 693.0)	985.7 (811.2 to 1181.0)	431.9 (208.0 to 648.6)	1.78 (1.32 to 2.44)	1.53 (1.05 to 2.24)
1095	17	31	755.3 (577.7 to 925.1)	1363.4 (1155.6 to 1599.9)	608.1 (338.8 to 881.9)	1.81 (1.39 to 2.39)	1.82 (1.05 to 3.44)
1460	17	14	1005.2 (801.4 to 1204.7)	1568.0 (1330.0 to 1827.0)	562.8 (242.1 to 876.0)	1.56 (1.21 to 2.04)	0.82 (0.37 to 1.73)
1825	6	16	1112.3 (902.0 to 1327.0)	1850.5 (1583.5 to 2124.2)	738.1 (380.4 to 1083.6)	1.66 (1.30 to 2.12)	2.67 (1.10 to 10.76)
2190	6	20	1242.4 (1011.9 to 1492.3)	2293.3 (1950.8 to 2634.2)	1050.9 (650.5 to 1438.8)	1.85 (1.46 to 2.34)	3.33 (1.55 to 10.50)
2555	7	8	1435.1 (1160.6 to 1726.2)	2509.3 (2129.3 to 2880.9)	1074.2 (586.8 to 1486.6)	1.75 (1.38 to 2.23)	***
2920	3	6	1538.5 (1253.1 to 1849.4)	2725.4 (2313.0 to 3141.7)	1186.9 (683.6 to 1685.6)	1.77 (1.40 to 2.28)	***
3285	3	2	1694.2 (1333.2 to 2042.5)	2810.2 (2386.0 to 3234.5)	1116.0 (600.8 to 1650.8)	1.66 (1.32 to 2.13)	***
3650	6	2	2092.5 (1644.8 to 2596.7)	2934.6 (2449.8 to 3390.7)	842.1 (153.8 to 1470.5)	1.40 (1.06 to 1.84)	***
4015	1	3	2193.4 (1710.5 to 2809.7)	3237.1 (2650.1 to 3843.0)	1043.7 (246.4 to 1777.3)	1.48 (1.10 to 1.97)	***
4380	1	0	2486.8 (1798.4 to 3324.5)	3237.1 (2650.1 to 3843.0)	750.3 (-190.2 to 1709.8)	1.30 (0.94 to 1.91)	***
4745	0	0	2486.8 (1798.4 to 3324.5)	3237.1 (2650.1 to 3843.0)	750.3 (-190.2 to 1709.8)	1.30 (0.94 to 1.91)	***
5110	0	0	2486.8 (1798.4 to 3324.5)	3237.1 (2650.1 to 3843.0)	750.3 (-190.2 to 1709.8)	1.30 (0.94 to 1.91)	***

*** IRR/RR was not estimated when there were fewer than 20 events in the risk period or fewer than 5 events in one group. Confidence intervals obtained through percentile bootstrap.

Supplementary Figures:

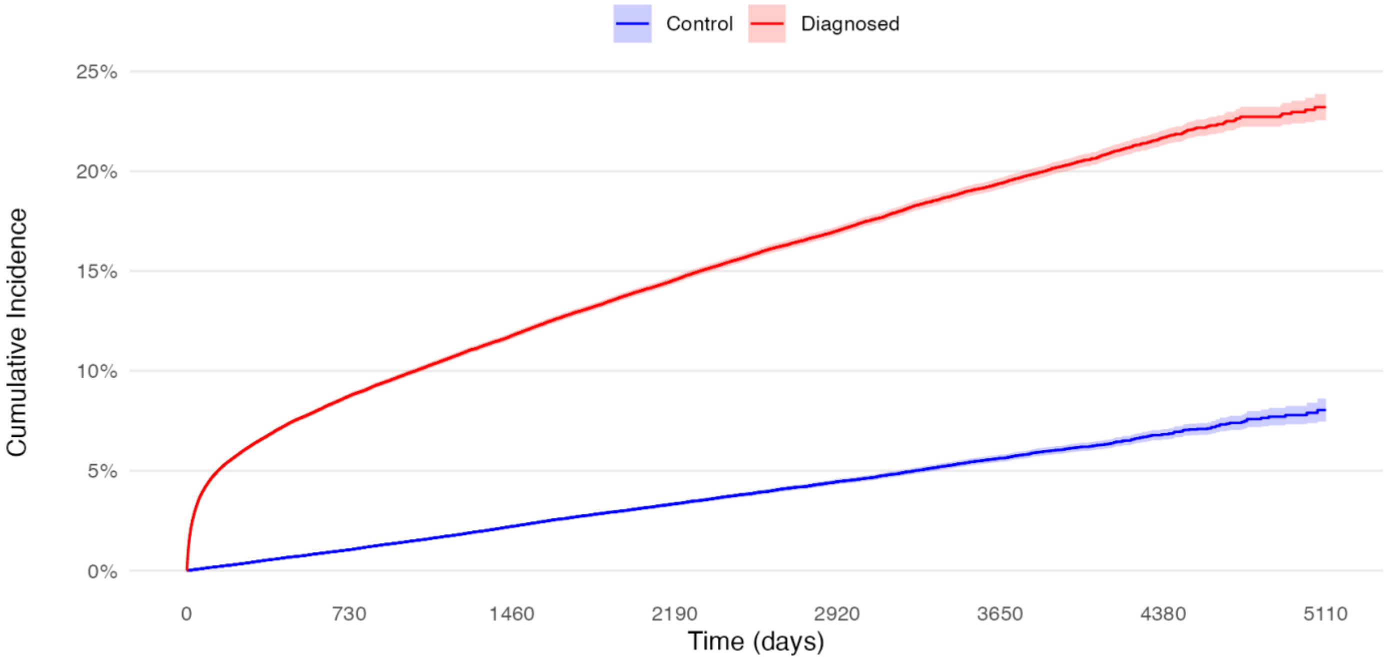

	0	730	1460	2190	2920	3650	4380	5110
**Control**								
No. risk	185,921	137,987	100,655	69,149	42,750	21,873	6,046	566
Events	0	1,694	3,103	4,092	4,730	5,124	5,292	5,325
**Diagnosed**								
No. risk	185,921	127,320	91,079	61,428	37,382	18,843	5,121	468
Events	0	15,155	18,822	21,267	22,708	23,525	23,855	23,900

Supplementary Figure 1. Cumulative incidence curve of all-cause mortality in the diagnosed tuberculosis cases, and the unexposed control group. Shaded areas indicate the 95% confidence intervals.

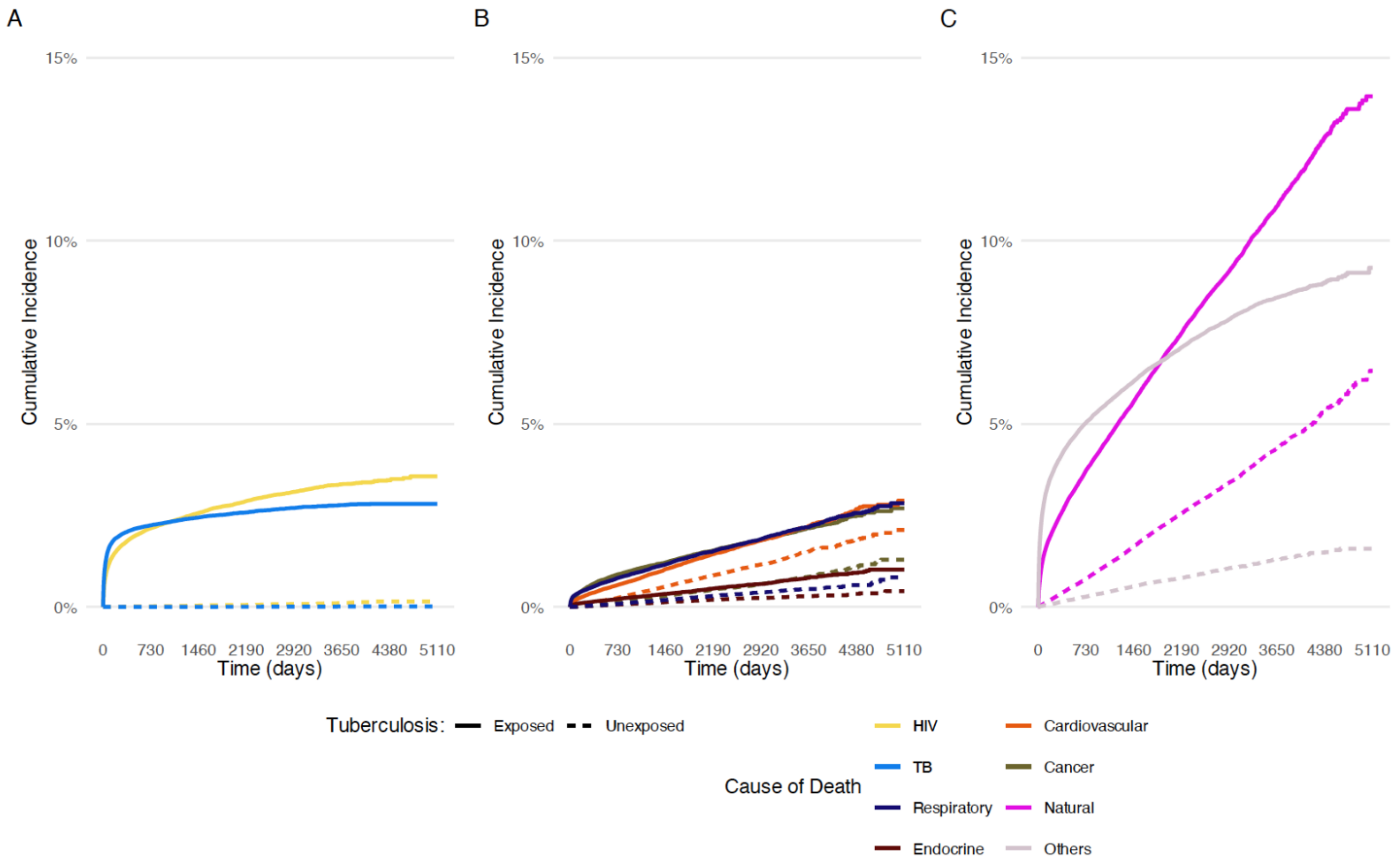

Supplementary Figure 2. Cumulative incidence curve of cause-specific mortality in the diagnosed tuberculosis cases and the unexposed control group. a) HIV and TB deaths, b) Cancer, endocrine, respiratory and cardiovascular deaths, c) Natural deaths (i.e., deaths excluding HIV/TB and external causes)

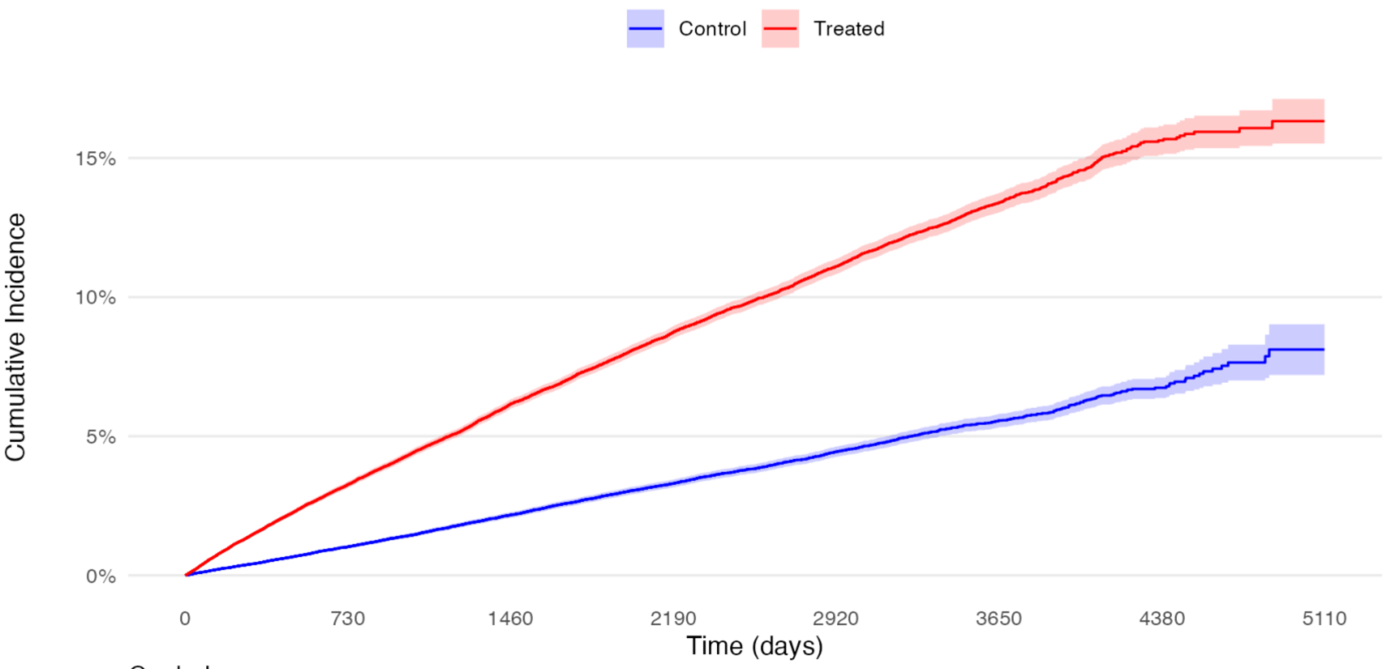

**Control**								
No. risk	111,871	81,104	55,588	37,514	22,698	10,637	1,932	39
Events	0	988	1,776	2,314	2,667	2,864	2,940	2,954
**Treated**								
No. risk	111,871	79,309	53,442	35,485	21,194	9,811	1,769	39
Events	0	3,174	5,170	6,387	7,120	7,525	7,670	7,677

Supplementary Figure 3. Cumulative incidence curve of all-cause mortality in the treated tuberculosis cases and the unexposed control group. Shaded areas indicate the 95% confidence intervals.

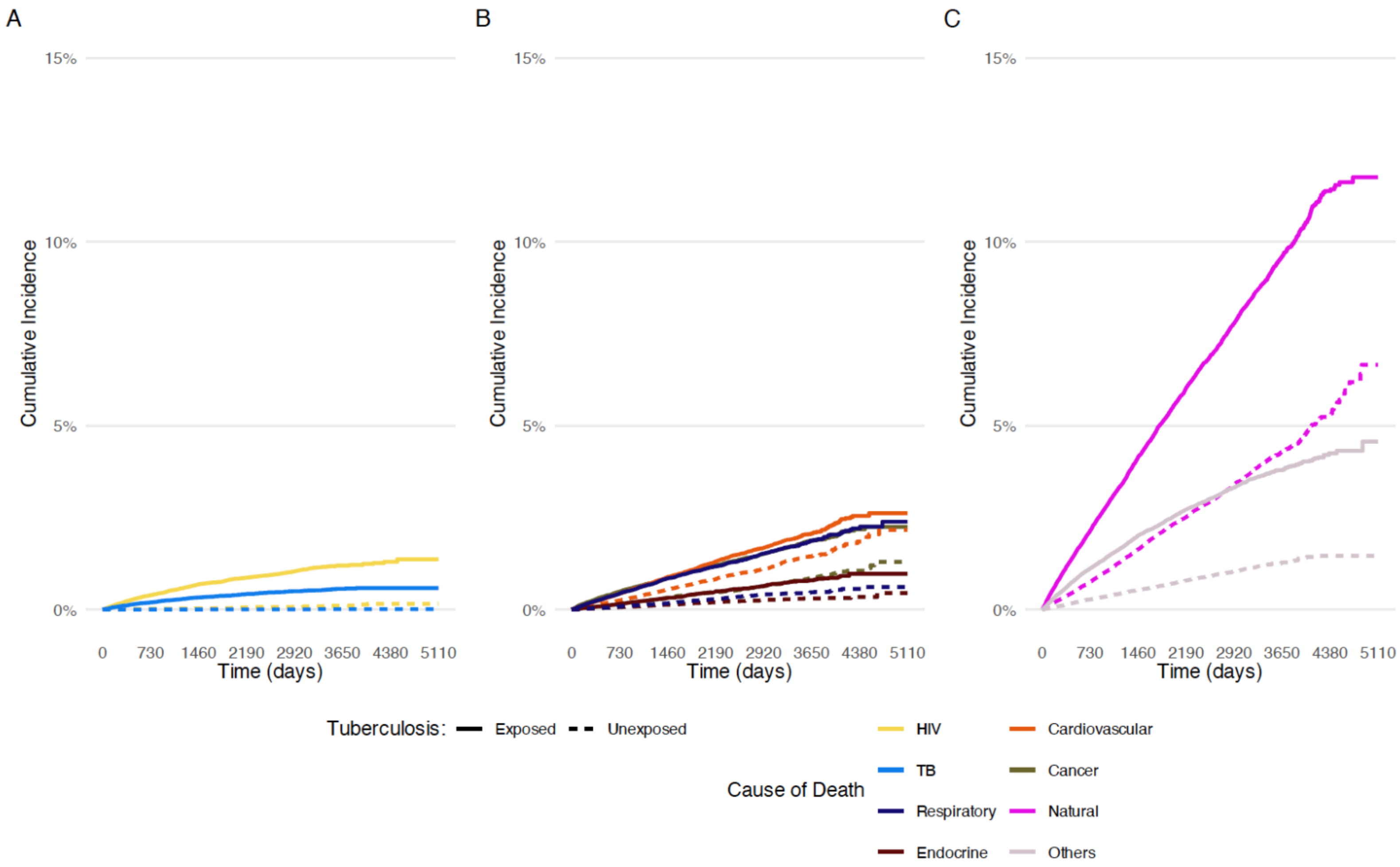

Supplementary Figure 4. Cumulative incidence curve of cause-specific mortality in the treated tuberculosis cases and the unexposed control group. a) HIV and TB deaths, b) Cancer, endocrine, respiratory and cardiovascular deaths, c) Natural deaths (i.e., deaths excluding HIV/TB and external causes)

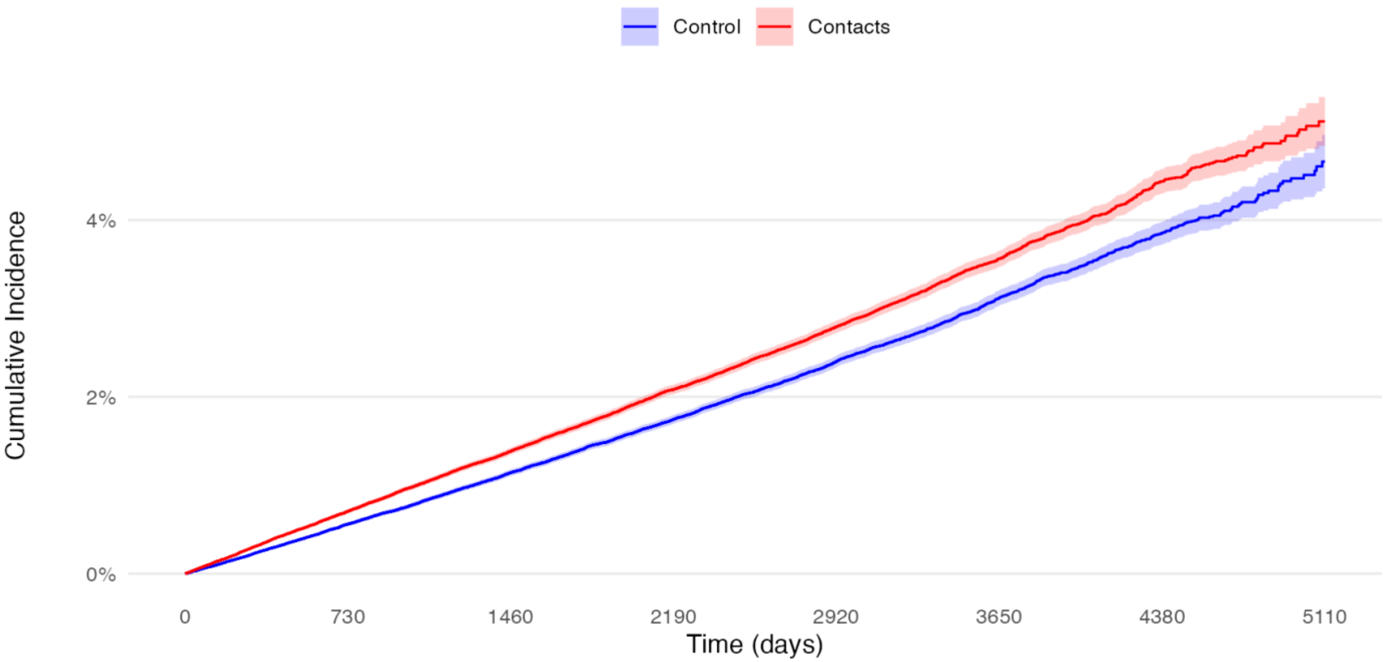

	0	730	1460	2190	2920	3650	4380	5110
**Control**								
No. risk	448,825	336,402	247,898	172,903	108,337	55,946	15,899	1,646
Events	0	2,188	3,889	5,170	6,098	6,689	6,954	7,004
**Contacts**								
No. risk	448,825	335,924	247,252	172,255	107,840	55,612	15,776	1,616
Events	0	2,766	4,765	6,248	7,254	7,897	8,205	8,250

Supplementary Figure 5. Cumulative incidence curve of all-cause mortality in the household tuberculosis case contacts and the unexposed control group. Shaded areas indicate the 95% confidence intervals.
