# Supplementary material for: Long-term risk of death after tuberculosis diagnosis and treatment
Source: Nat Med. 2026 Mar 19;32(5):1927–34. doi: 10.1038/s41591-026-04294-w (PMC13190314; doi:10.1038/s41591-026-04294-w)
Supplement: Supplementary file 2 — Reporting Summary [file 41591_2026_4294_MOESM2_ESM.pdf]

Corresponding author(s): Thiago Cerqueira Silva

Last updated by author(s): Feb 10, 2026

Reporting Summary

Nature Portfolio wishes to improve the reproducibility of the work that we publish. This form provides structure for consistency and transparency in reporting. For further information on Nature Portfolio policies, see our Editorial Policies and the Editorial Policy Checklist.

Statistics

For all statistical analyses, confirm that the following items are present in the figure legend, table legend, main text, or Methods section.

n/a	Confirmed	
☐	☒	The exact sample size (*n*) for each experimental group/condition, given as a discrete number and unit of measurement
☒	☐	A statement on whether measurements were taken from distinct samples or whether the same sample was measured repeatedly
☒	☐	The statistical test(s) used AND whether they are one- or two-sided<br>*Only common tests should be described solely by name; describe more complex techniques in the Methods section.*
☐	☒	A description of all covariates tested
☐	☒	A description of any assumptions or corrections, such as tests of normality and adjustment for multiple comparisons
☐	☒	A full description of the statistical parameters including central tendency (e.g. means) or other basic estimates (e.g. regression coefficient) AND variation (e.g. standard deviation) or associated estimates of uncertainty (e.g. confidence intervals)
☒	☐	For null hypothesis testing, the test statistic (e.g. *F*, *t*, *r*) with confidence intervals, effect sizes, degrees of freedom and *P* value noted<br>*Give P values as exact values whenever suitable.*
☒	☐	For Bayesian analysis, information on the choice of priors and Markov chain Monte Carlo settings
☒	☐	For hierarchical and complex designs, identification of the appropriate level for tests and full reporting of outcomes
☒	☐	Estimates of effect sizes (e.g. Cohen's *d*, Pearson's *r*), indicating how they were calculated

Our web collection on statistics for biologists contains articles on many of the points above.

Software and code

Policy information about availability of computer code

Data collection	R version 4.1.1
Data analysis	R version 4.1.1; packages: survival; tidyverse; EValue

For manuscripts utilizing custom algorithms or software that are central to the research but not yet described in published literature, software must be made available to editors and reviewers. We strongly encourage code deposition in a community repository (e.g. GitHub). See the Nature Portfolio guidelines for submitting code & software for further information.

Data

Policy information about availability of data

All manuscripts must include a data availability statement. This statement should provide the following information, where applicable:

- Accession codes, unique identifiers, or web links for publicly available datasets
- A description of any restrictions on data availability
- For clinical datasets or third party data, please ensure that the statement adheres to our policy

The relevant data are available in the manuscript and the appendix. Raw data are available upon reasonable request to the Centro de Integração de Dados e Conhecimentos para a Saúde (CIDACS). Any person who wishes to receive authorisation must: (1) be affiliated to CIDACS or be accepted as collaborators; (2) present a detailed research project together with approval by an appropriate Brazilian institutional research ethics committee; (3) provide a clear data plan restricted to the objectives of the proposed study and a summary of the analyses plan intended to guide the linkage and data extraction of the relevant set of

records and variables; (4) sign terms of responsibility regarding the access and use of data; and (5) perform the analyses of datasets provided using the CIDACS data environment, a safe and secure infrastructure that provides remote access to de-identified or anonymised datasets and analysis tools. For more information: https://cidacs.bahia.fiocruz.br/

Research involving human participants, their data, or biological material

Policy information about studies with human participants or human data. See also policy information about sex, gender (identity/presentation), and sexual orientation and race, ethnicity and racism.

Reporting on sex and gender	We used sex (biological attribute) when describing the sex of study population. We also conducted stratified analysis by sex
Reporting on race, ethnicity, or other socially relevant groupings	We used race (classified in accordance to the IBGE - Instituto Brasileiro de Geografia e Estatística), white/black/mixed/asian-yellow/indigenous. This variable is self-reported. Subgroup analysis by race/ethnicity was conducted to assess structural racism.
Population characteristics	All the information avaliable in the database is presented in the Table 1, Supplementary Table 1,2,3 of the manuscrirpt
Recruitment	This is a observational study using data from national administrative databases
Ethics oversight	The study was approved by the ethics committees of the Instituto Gonçalo Muniz–Oswaldo Cruz Foundation (1.612.302), Salvador, Brazil.

Note that full information on the approval of the study protocol must also be provided in the manuscript.

Field-specific reporting

Please select the one below that is the best fit for your research. If you are not sure, read the appropriate sections before making your selection.

☒ Life sciences ☐ Behavioural & social sciences ☐ Ecological, evolutionary & environmental sciences

For a reference copy of the document with all sections, see nature.com/documents/nr-reporting-summary-flat.pdf

Life sciences study design

All studies must disclose on these points even when the disclosure is negative.

Sample size	Given that the sample sizes were based on national linked datasets; we only excluded individuals that do not fit the eligibility criteria. Resulting in 371,842 participants.
Data exclusions	We excluded individuals (i) aged 100 years or older at CadÚnico registration, (ii) diagnosed with tuberculosis before the CadÚnico registration, (iii) with missing data in one of the variables used in the matching (Supplementary Table 1), (iv) data inconsistencies in date of death before diagnosis date or date of death before date of CadÚnico registration, (v) people experiencing homelessness due to the impossibility of assessing variables related to the household; (vi) participants with a TB diagnosis recorded as relapse or retreatment. For the treated cohort, we only included TB cases that were classified as “cured” in SINAN-TB. To remove records with inconsistent treatment length, we also excluded individuals whose treatment completion date was missing, less than 138 days after the notification date or more than 2 years after the notification date. We used this cut-off considering the treatment duration of 6 - 12 months, as recommended by the Ministry of Health in Brazil, depending on the type of Tuberculosis, the maximum of two years was chosen to allow for delays in the start of treatment after diagnosis.
Replication	Not applicable
Randomization	Not applicable (observational study)
Blinding	Not applicable (observational study)

Reporting for specific materials, systems and methods

We require information from authors about some types of materials, experimental systems and methods used in many studies. Here, indicate whether each material, system or method listed is relevant to your study. If you are not sure if a list item applies to your research, read the appropriate section before selecting a response.

Materials & experimental systems

n/a	Involved in the study
☒	☐ Antibodies
☒	☐ Eukaryotic cell lines
☒	☐ Palaeontology and archaeology
☒	☐ Animals and other organisms
☒	☐ Clinical data
☒	☐ Dual use research of concern
☒	☐ Plants

Methods

n/a	Involved in the study
☒	☐ ChIP-seq
☒	☐ Flow cytometry
☒	☐ MRI-based neuroimaging

Plants

Seed stocks	NA
Novel plant genotypes	NA
Authentication	NA
